# Genome-wide analysis of the Glycerol-3-Phosphate Acyltransferase (GPAT) gene family reveals the evolution and diversification of plant GPATs

**DOI:** 10.1590/1678-4685-GMB-2017-0076

**Published:** 2018-03-19

**Authors:** Edgar Waschburger, Franceli Rodrigues Kulcheski, Nicole Moreira Veto, Rogerio Margis, Marcia Margis-Pinheiro, Andreia Carina Turchetto-Zolet

**Affiliations:** 1 Programa de Pós-Graduação em Genética e Biologia Molecular, Departamento de Genética, Universidade Federal do Rio Grande do Sul (UFRGS), Porto Alegre, RS, Brazil; 2 Centro de Biotecnologia e Programa de Pós-Graduação em Biologia Celular e Molecular, Universidade Federal do Rio Grande do Sul (UFRGS), Porto Alegre, RS, Brazil; 3 Departamento de Biofísica, Universidade Federal do Rio Grande do Sul (UFRGS), Porto Alegre, RS, Brazil; 4 Departamento de Biologia Celular, Embriologia e Genética, Universidade Federal de Santa Catarina (UFSC), Florianópolis, SC, Brazil; 5 Graduação em Biotecnologia, Departamento de Biologia Molecular e Biotecnologia, Universidade Federal do Rio Grande do Sul (UFRGS), Porto Alegre, RS, Brazil

**Keywords:** Plant lipids, GPAT enzymes, phylogeny, evolution, gene expression

## Abstract

*sn*-Glycerol-3-phosphate 1-O-acyltransferase (GPAT) is an important enzyme that catalyzes the transfer of an acyl group from acyl-CoA or acyl-ACP to the sn-1 or sn-2 position of *sn-*glycerol-3-phosphate (G3P) to generate lysophosphatidic acids (LPAs). The functional studies of GPAT in plants demonstrated its importance in controlling storage and membrane lipid. Identifying genes encoding GPAT in a variety of plant species is crucial to understand their involvement in different metabolic pathways and physiological functions. Here, we performed genome-wide and evolutionary analyses of GPATs in plants. GPAT genes were identified in all algae and plants studied. The phylogenetic analysis showed that these genes group into three main clades. While clades I (GPAT9) and II (soluble GPAT) include GPATs from algae and plants, clade III (GPAT1-8) includes GPATs specific from plants that are involved in the biosynthesis of cutin or suberin. Gene organization and the expression pattern of GPATs in plants corroborate with clade formation in the phylogeny, suggesting that the evolutionary patterns is reflected in their functionality. Overall, our results provide important insights into the evolution of the plant GPATs and allowed us to explore the evolutionary mechanism underlying the functional diversification among these genes.

## Introduction

Lipids from plants are composed of several types of fatty acids and their derivatives, such as lipid polyesters, glycerolipids and sterols. They are involved in a wide range of metabolic reactions, playing important physiological roles in plant development, as major components of cellular membranes, storage, extracellular protective layers and signaling molecules (Chen *et al.*, 2011a). A complex network of genes and proteins is involved and controls the biosynthesis of different lipids. *sn*-Glycerol-3-phosphate 1-O-acyltransferase (GPAT; Enzyme Commission [EC] 2.3.1.15) is an important enzyme in glycerolipid biosynthesis, which is involved in different metabolic pathways and physiological functions. GPAT catalyzes the first step in the synthesis of almost all membrane phospholipids. GPAT transfers an acyl group from acyl-CoA or acyl-ACP at the sn-1 or -2 position of a glycerol 3-phosphate generating lysophosphatidic acids (LPAs) (Zheng *et al.*, 2003; Takeuchi and Reue, 2009). LPA is a substrate for the production of several important glycerolipid intermediates, such as storage lipids, extracellular lipid polyesters and membrane lipids (Li-Beisson *et al.*, 2013).

Other enzymes involved in triacylglycerol (TAG) biosynthesis have also been studied. Diacylglycerol acyltransferase (DGAT; EC 3.2.1.20) was demonstrated to be crucial for enhancing the control of seed oil content through bioengineering (Liu *et al.*, 2012). These enzymes have also been widely studied in relation to their evolutionary history (Turchetto-Zolet *et al.*, 2011, 2016). Evolutionary studies were also performed for lysophosphatidic acid acyltransferase (LPAAT, EC 2.3.1.51) (Körbes *et al.*, 2016), that uses lysophosphatidic acid (LPA) to yield phosphatidic acid (PA), and for phospholipid:diacylglycerol acyltransferase (PDAT; EC 2.3.1.158) (Pan *et al.*, 2015). Identifying all acyltransferases genes involved in plant glycerolipid biosynthesis, such as GPAT, is crucial for the understanding the involvement of these genes in different metabolic pathways and physiological functions. Besides, this knowledge can contribute to the development of engineered plant oils containing desired nutritional or industrial properties.

GPATs were first characterized biochemically over 60 years ago from animal and plant tissues (Weiss *et al.*, 1939; Kornberg and Pricer, 1987). The reaction involving GPAT activity has already been characterized in bacteria (Zhang and Rock, 2008), fungi (Zheng and Zou, 2001), animals (Gimeno and Cao, 2008; Wendel *et al.*, 2009), and plants (Murata and Tasaka, 1997; Chen *et al.*, 2011a; Yang *et al.*, 2012). Different GPATs were characterized in plants and their activity was observed in three distinct plant subcellular compartments, i.e., plastid, endoplasmic reticulum (ER) and mitochondria (Gidda *et al.*, 2009). The mitochondrial and ER GPATs are membrane-bound forms with acyl-CoA and acyl-ACP as natural acyl donors, while the plastidial GPAT is a soluble form and uses acyl-ACP as its natural acyl substrate (Zheng *et al.*, 2003). Comparative analysis of GPATs from evolutionarily diverse organisms has revealed that these enzymes contain at least four highly conserved amino acid sequence motifs that are essential for both acyltransferase activity and the glycerol-3-phosphate substrate binding (Lewin *et al.*, 1999).

Studies demonstrated the presence of 10 GPAT genes in the model plant *Arabidopsis thaliana* genome, named GPAT1-9 and Soluble GPAT (plastidial form). GPAT9 plays an essential role in plant membrane and storage lipid biosynthesis (Gidda *et al.*, 2009; Chen *et al.*, 2011a; Shockey *et al.*, 2015). The plastidial form of GPATs (also known as ATS1 in Arabidopsis) is involved in the *de novo* biosynthesis of glycerolipids within chloroplasts (Ohlrogge and Browse, 1995). Its fatty acid substrates are synthesized within the chloroplast, yielding 16:0-ACP, 18:0-ACP and 18:1-ACP, which can be used by either the soluble GPATs or hydrolyzed by acyl-ACP thioesterases (Sánchez-García *et al.*, 2010). Certain evidence suggests that acyl substrate preference (i.e., saturated vs. unsaturated acyl-ACPs) of the plastidial soluble GPAT may partially control the chilling tolerance in plants, by mediating the fatty acid composition at the sn-1 position of phosphatidylglycerol (PG), and, thus, affecting membrane fluidity of the plant aerial tissue (Nishida *et al.*, 1987, 1993). Finally, the remaining eight GPATs (GPAT1-8) (Zheng *et al.*, 2003; Beisson *et al.*, 2007; Gidda *et al.*, 2009; Yang *et al.*, 2012) are not required for membrane or storage lipid biosynthesis, but may affect the composition and quantity of cutin or suberin in *Arabidopsis thaliana* (Beisson *et al.*, 2012; Yang *et al.*, 2012); *Brassica napus* (Chen *et al.*, 2011b, 2014) and *Oryza sativa* (Men *et al.*, 2017).

Previous studies revealed that: (i) there are multiple copies of GPAT genes in plant genomes, (ii) different GPAT gene paralogs can encode enzymes with different glycerolipid synthesizing ability, and (3) GPATs may be involved in many different metabolic and physiologic pathways. All these findings shed new light on glycerolipid biosynthetic pathway in plants and emphasize the need for a deeper understanding of the complexity of plant GPATs. In this study, we performed a genome-wide comparative analysis, including a phylogenetic approach, gene structure comparison and gene expression analyses to provide further insights into the present-day diversity and ortholog/paralog relationship of plant GPATs.

## Materials and Methods

### Identification of GPAT genes and their homologs in plants

To identify GPAT genes and their homologs, we first performed a literature survey to find GPAT genes that have already been characterized in the model plant *A. thaliana*. Then, we retrieved the *A. thaliana* GPAT sequences using BLAST and keyword searches in the Phytozome database (http://www.phytozome.net/). The *A. thaliana* GPATs sequences (soluble GPAT [AT1G32200], GPAT1 [AT1G06520], GPAT2 [AT1G02390], GPAT3 [AT2G38110], GPAT4 [AT4G01950], GPAT5 [AT3G11430], GPAT6 [AT1G01610], GPAT7 [AT5G06090], GPAT8 [AT4G00400] and GPAT9 [AT5G60620]) were used as queries to perform BLASTp and TBLASTx searches in the Phytozome database. BLAST searches were conducted against 39 plant species genomes and proteomes available in Phytozome, including algae (*Chlamydomonas reinhardtii*, *Volvox carteri*, *Coccomyxa subellipsoidea* C-169, *Micromonas pusilla* CCMP1545, *Micromonas pusilla sp* and *Ostreococcus lucimarinus*); the lycophyte *Selaginella moellendorffii*, the mosses *Physcomitrella patens* and *Sphagnum fallax;* the single living representative of the sister lineage to all other extant flowering plants *Amborella trichopoda*; monocots (*Brachypodium distachyon, Oryza sativa*, *Panicum hallii, Setaria italica, Setaria viridis*, *Sorghum bicolor* and *Zea mays*); and eudicots (*Aquilegia coerulea*, *Mimulus guttatus*, *Manihot esculenta*, *Ricinus communis*, *Populus trichocarpa*, *Medicago truncatula*, *Phaseolus vulgaris*, *Glycine max*, *Cucumis sativus*, *Arabidopsis lyrata*, *Arabidopsis thaliana*, *Eutrema salsugineum*, *Capsella rubella*, *Capsella grandiflora*, *Brassica rapa*, *Gossypium raimondii*, *Theobroma cacao*, *Citrus sinensis, Citrus clementina*, *Eucalyptus grandis*, *Solanum tuberosum*, *Solanum lycopersicum*). The cDNA, genomic DNA, and amino acid sequences corresponding to each GPAT or putative GPAT were downloaded from the Phytozome database. All taxa were indicated by three-letter acronyms in which the first letter is the first letter of the genus and the next two letters are the first two letters of the species name (e.g. Osa corresponds to *O. sativa*). The sequences were identified in all analyses using the acronym followed by the protein accession number (e.g., Osa_LOC_Os01g44069 corresponds to *Oryza sativa*). The names for the previously reported *A. thaliana* GPATs were added before the acronym, and the accession number (e.g., GPAT1-Ath_AT1G06520 corresponds to *A. thaliana* GPAT1). A detailed description of the sequences used in this study, including their corresponding accession numbers, protein length, presence of protein domain and intron numbers is provided in Supplementary Table S1.

### Sequence alignment and phylogenetic analyses

The nucleotide and protein sequences were aligned using MUSCLE (Edgar, 2004) implemented in Molecular Evolutionary Genetics Analysis - MEGA version 7.0 (Kumar *et al.*, 2016). The multiple alignments were manually inspected and edited and only unambiguously aligned positions were included in the final analysis. The phylogenetic relationships were reconstructed following nucleotide and protein sequence alignments using a Bayesian method carried out in BEAST1.8.4 (Drummond *et al.*, 2012). ProTest 2.4 (Abascal *et al.*, 2005) was used to select the best model of protein evolution. The JTT+I+G model was the best model indicated by ProtTest for the protein sequences dataset. The best model for nucleotide evolution was selected in jModelTest (Posada, 2008), and the best fit model was GTR+I+G. The Birth-death processes was selected as a tree prior to Bayesian analysis, and was run for 60,000,000 generations with Markov chain Monte Carlo (MCMC) algorithms for both amino acid and nucleotide sequences. Tracer 1.6 (Rambaut *et al.*, 2014; http://beast.bio.ed.ac.uk/Tracer) was used to verify the convergence of the Markov chains and the adequate effective sample sizes (> 200). The trees were visualized and edited using FigTree v1.4.3 (http://tree.bio.ed.ac.uk/software/figtree).

### Gene structure analysis

In order to determine the intron/exon distribution in the GPAT genes of plants and understand the rules and possible consequences of gene structure and organization on protein functionality and evolutionary changes among species (Wang *et al.*, 2013), a comparative analysis of exon/intron organization was performed from genomic DNA sequences deposited in the Piece2.0 databases (Wang *et al.*, 2016). Basically, we submitted a query sequence set (in multi-FASTA format) consisting of genomic and CDS for GPATs and putative GPATs from seven representative species (*A. thaliana*, *G. max*, *O. sativa*, *B. distachyon, S. moellendorffii*, *P. patens* and *V. carteri*) to GSDraw and retrieved the gene structures with conserved protein motifs and phylogenetic trees. We also performed searches for each GPAT in all species deposited in the Piece database and retrieved gene structure organization and intron phase for all these species. The online Gene Structure Display Server (Guo *et al.*, 2007; http://gsds.cbi.pku.edu.cn) was also used to analyze the intron/exon distribution and intron phase patterns along with the phylogenetic tree for the seven species cited above.

### Detection of transmembrane domains and conserved motifs

Potential transmembrane domains in GPAT protein sequences were predicted using the TMHMM-2.0 program (Krogh *et al.*, 2001) provided by the CBS Prediction Servers (http://www.cbs.dtu.dk/services/TMHMM-2.0/) and in [Bibr B62] PROTTER (Omasits *et al.*, 2014) in representative species (*A. thaliana*, *O. sativa*, *S. moellendorffii*, *P. patens* and *V. carteri*). Potential functional motifs of GPAT proteins were identified using the multiple expectation maximization for motif elicitation (MEME) utility program (http://meme.sdsc.edu) (Bailey *et al.*, 2006). The sequence logo was constructed with WebLogo (http://weblogo.berkeley.edu/logo.cgi) (Crooks *et al.*, 2004).

### Gene expression prediction

Microarray data available at the GENEVESTIGATOR web site (https://www.genevestigator.com) (Hruz *et al.*, 2008) were used to determine tissue specificity and intensity of expression of GPAT and putative GPAT genes of *A. thaliana*, *G. max*, *O. sativa* and *Z. mays*. The Hierarchical Clustering tool implemented in GENEVESTIGATOR was used to perform this analysis. The highest expression values were considered for genes with more than one probe set. The expression data were gene-wise normalized and hierarchically clustered based on Pearson coefficients. The percent expression potential of GPAT and putative GPAT genes in different anatomical regions and developmental stages was represented in heat maps.

## Results

### Genome-wide identification of GPAT homologs sequences in plants

Currently, there are 10 genes annotated as GPATs in the *A. thaliana* genome. These 10 genes are named as: GPAT9, which is localized to the endoplasmic reticulum (ER); soluble GPAT, located in the plastid; and GPAT1-8, of which GPAT1-3 are localized in mitochondria and GPAT4-8 in ER. Using nucleotide and amino acid sequences from these 10 *A. thaliana* GPATs as queries, we conducted a broad survey of fully sequenced genomes for the presence of GPAT homologs genes in 39 species (six algae and 33 land plants) (Table 1, Tables S1 and S2). Candidate GPAT genes were found in all examined plant genomes. Interestingly, the BLAST searches against *A. thaliana* returned 11 putative GPAT sequences, among them 10 are known GPATs (soluble GPAT, GPAT9 and GPAT1-8). One of these, AT3G11325, is annotated as a member of the phospholipid/glycerol acyltransferase protein family in the Phytozome database and is more similar to Arabidopsis GPAT5 and GPAT7 (80% and 76.2%, respectively).

**Table 1:** Taxonomy data, number of GPATs per species, and clade distribution based on the phylogeny.

				Clade		
Family	Species	Acronymon	Number of genes	I (GPAT9)	II (Soluble GPAT)	III (GPAT1-8)
Funariaceae	*Physcomitrella patens*	Ppa	9	1	1	7
Sphagnaceae	*Sphagnum fallax*	Sfa	12	2	1	9
Selaginellaceae	*Selaginella moellendorffii*	Smo	12	1	1	10
Amborellaceae	*Amborella trichopoda*	Atr	7	0	1	6
Poaceae	*Brachypodium distachyon*	Bdi	18	1	1	16
Poaceae	*Oryza sativa*	Osa	18	1	1	16
Poaceae	*Panicum hallii*	Pha	18	1	1	16
Poaceae	*Setaria italica*	Sit	20	1	1	18
Poaceae	*Setaria viridis*	Svi	19	1	1	17
Poaceae	*Sorghum bicolor*	Sbi	16	1	1	14
Poaceae	*Zea mays*	Zma	17	2	1	14
Ranunculaceae	*Aquilegia coerulea*	Aco	15	2	1	12
Phrymaceae	*Mimulus guttatus*	Mgu	13	1	1	11
Solanaceae	*Solanum lycopersicum*	Sly	10	1	1	8
Solanaceae	*Solanum tuberosum*	Stu	13	1	0	12
Myrtaceae	*Eucalyptus grandis*	Egr	12	1	2	10
Euphorbiaceae	*Manihot esculenta*	Mês	11	1	0	10
Salicaceae	*Populus trichocarpa*	Ptr	10	0	1	9
Euphorbiaceae	*Ricinus communis*	Rco	10	1	1	8
Rutaceae	*Citrus sinensis*	Csi	9	1	0	8
Rutaceae	*Citrus clementina*	Ccl	10	1	1	8
Malvaceae	*Gossypium raimondii*	Gra	17	2	3	12
Malvaceae	*Theobroma cacao*	Tca	12	1	1	10
Brassicaceae	*Arabidopsis lyrata*	Aly	10	1	1	8
Brassicaceae	*Arabidopsis thaliana*	Ath	11	1	1	9
Brassicaceae	*Brassica rapa*	Bra	17	2	3	12
Brassicaceae	*Capsella grandiflora*	Cgr	11	1	1	9
Brassicaceae	*Capsella rubella*	Cru	11	1	1	9
Brassicaceae	*Eutrema salsugineum*	Esa	10	1	1	8
Cucurbitaceae	*Cucumis sativus*	Csa	8	1	1	6
Fabaceae	*Glycine max*	Gma	28	3	2	25
Fabaceae	*Medicago truncatula*	Mtr	12	2	1	9
Fabaceae	*Phaseolus vulgaris*	Pvu	12	2	1	9
Chlamydomonadaceae	*Chlamydomonas reinhardtii*	Cre	2	1	1	0
Volvocaceae	*Volvox carteri*	Vca	2	1	1	0
Coccomyxaceae	*Coccomyxa subellipsoidea*	Csu	2	1	1	0
Mamiellaceae	*Micromonas pusilla*	Mpu	2	1	1	0
Mamiellaceae	*Micromonas sp.*	Msp	2	1	1	0
Bathycoccaceae	*Ostreococcus lucimarinus*	Olu	3	1	2	0

GPAT genes were ubiquitously found in all algae and land plants studied. In total, we retrieved 450 sequences from 39 species (Table 1). The algae species have two putative GPATs genes, except for *O. lucimarinus* that presented three. The mosses *P. patens* and *S. fallax* presented nine and 12 putative GPAT genes, respectively. The lycophyte *S. moellendorffii* presented 12, while *A. trichopoda* has seven putative GPAT genes. Among the monocot species, *B. distachyon*, *O. sativa* and *P. hallii* presented 18 putative GPAT genes, while *S. italica*, *S. viridis*, *S. bicolor* and *Z. mays* presented 20, 19, 16 and 17 putative GPAT genes, respectively. In the eudicot species, the number of genes ranged from eight (*C. sativus*) to 28 (*G. max*). *C. sinensis* presented nine and *C. clementina*, *A. lyrata*, *E. salsugineum*, *S. lycopersicum*, *P. trichocarpa*, *R. comunis*, presented 10 putative GPAT genes. *M. esculenta*, *A. thaliana*, *C. grandiflora* and *C. rubella* presented 11 putative GPAT genes. *E. grandis*, *T. cacao*, *M. truncatula* and *P. vulgaris* presented 12, while *M. guttatus* and *S. tuberosum* presented 13 putative GPAT genes. *A. coerulea* presented 15 putative GPAT genes. *G. raimondii* and *B. rapa* presented 17 putative GPAT genes. To verify the reliability of the BLAST results, the 450 protein sequences retrieved were subjected to InterPro and Pfam analyses (Table S1), and most of them were classified into the acyltransferase family (Pfam: PF01553). This family contains acyltransferases involved in phospholipid biosynthesis and proteins of unknown function.

### Phylogenetic relationships of the GPATs in plants

**Figure 1:**
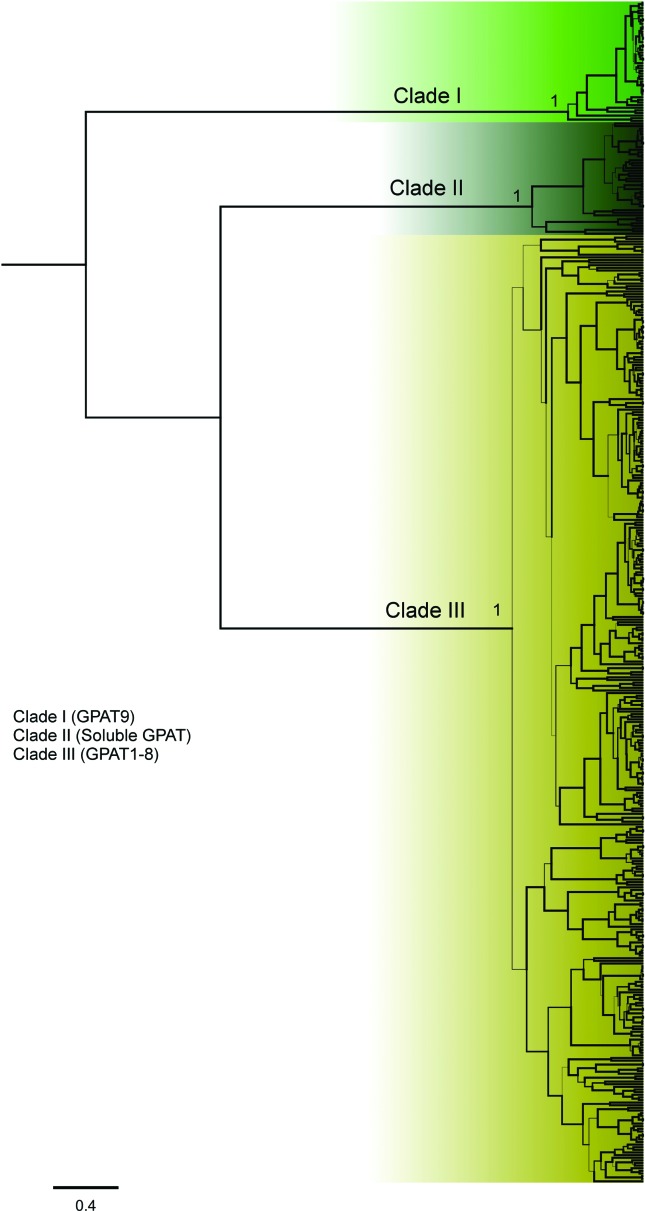
Phylogenetic relationship among plant and algae GPAT protein sequences. A total of total 450 protein sequences from six algae and 33 plant species were included in the analyses. The posteriori probabilities > 0.9 are labeled as thicker lines. Only values higher than 0.5 are presented. Three well-supported main clades were formed and were indicated by different colors in the phylogenetic tree.

**Figure 2:**
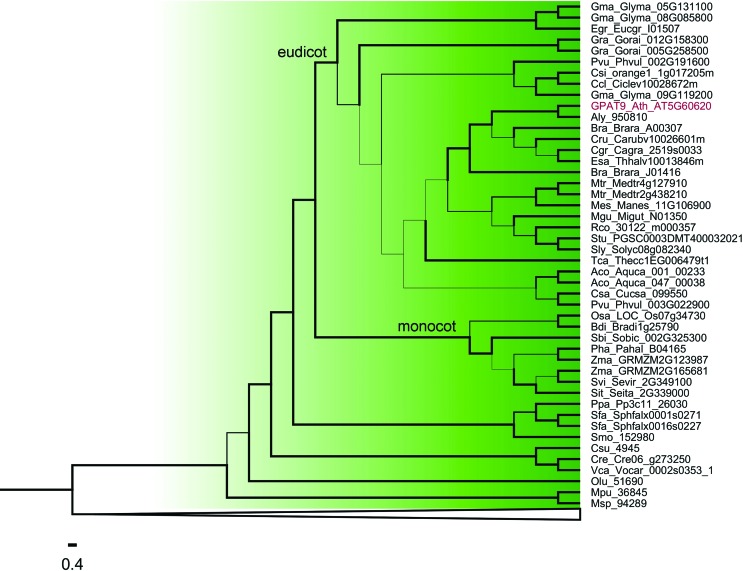
Phylogenetic relationships among GPAT genes belonging to Clade I from Figure 1. Thicker lines present posterior probability > 0.9. The complete list of species is presented in Table S1.

To investigate the evolutionary relationships among the plant GPATs, we reconstructed phylogenetic trees using the protein sequences of putative GPATs identified by homology searches in 39 species. In Figure 1, a compact view of the tree based on protein sequences is shown (the entire, expanded view, including species names and accession numbers, can be found in Figures 2 [Fig f3] [Fig f4]–5). The phylogenetic analysis of GPAT amino acid sequences resulted in a well-resolved tree, revealing the formation of three main clades (Figure 1). The first one (named clade I) includes GPAT9 sequences, the second (clade II) includes the soluble GPAT sequences, and the third (clade III) includes GPAT1-8 and GPAT-like proteins. The algal GPAT sequences are placed in the GPAT9 and soluble GPAT clades, suggesting that these GPATs are the most ancient forms. No algae GPATs were placed within the GPAT1-8 clade (clade III), indicating that these GPATs are plant specific and evolved in land plants to provide pathways for functions not present in other organisms.

**Figure 3 f3:**
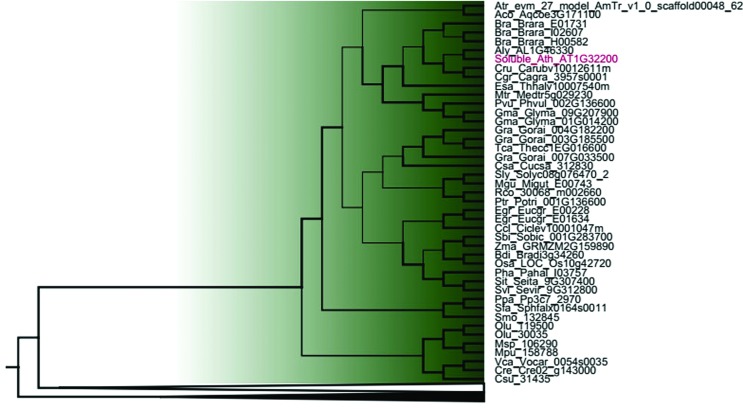
Phylogenetic relationships among GPAT genes belonging to Clade II from Figure 1. Thicker lines present posterior probability > 0.9. The complete list of species is presented in Table S1.

**Figure 4 f4:**
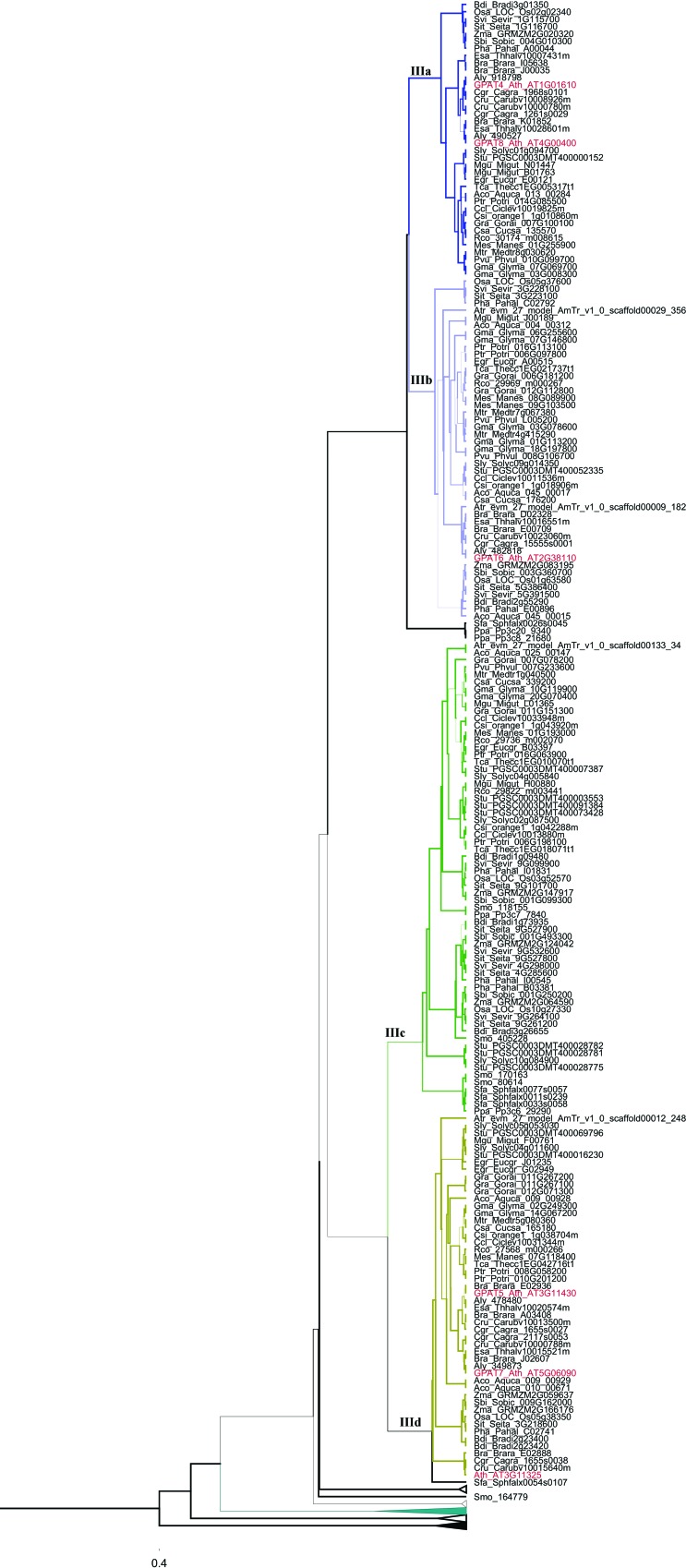
Phylogenetic relationships among GPAT genes belonging to Clade III (subclades IIIa, IIIb, IIIc and IIId) from Figure 1. Thicker lines present posterior probability > 0.9.

**Figure 5:**
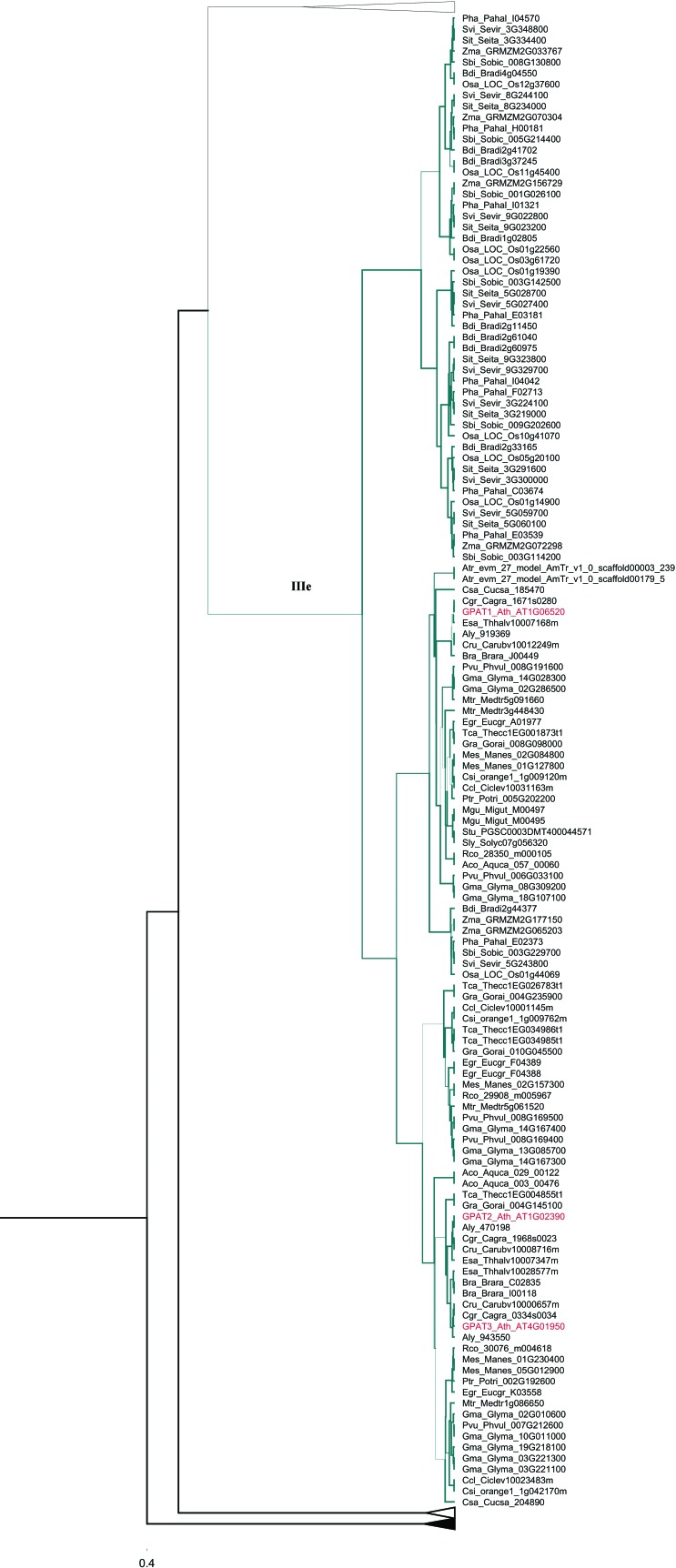
Phylogenetic relationships among GPAT genes belonging to Clade III (subclades IIIe) from Figure 1. Thicker lines present posterior probability > 0.9.

Within clade I (GPAT9) (Figure 2) and clade II (soluble GPAT) (Figure 3), the algal GPAT9 and algal soluble GPAT are phylogenetically divergent from the land plant GPAT9 and land plant soluble GPAT. Among the land plants, GPATs from basal plants (moss and lycophyte), monocots and eudicots species diverged from each other and formed distinct clusters. Most of the species studied present only one sequence of GPAT9 and soluble GPAT, except for *G. max*, *G. raimondii*, *B. rapa*, *M. truncatula*, *E. grandis* and *Z. mays*, these possibly presenting gene duplication events.

Clade III (GPAT1-8) (Figures 4 and 5) is subdivided into five subclades: IIIa groups GPAT4 and GPAT8; IIIb groups GPAT6; IIIc includes a group of sequences that we named GPAT-like with no representative from the Brassicales order; IIId includes GPAT5 and GPAT7; IIIe includes GPAT1-3. Within subclade IIIe we observed a separate group of sequences that included only monocot species related to the well characterized GPAT3 from *O. sativa*. The GPATs from *P. patens* and *S. fallax* (moss) and *S. moellendorffii* (lycophyte), two basal lineages of land plants, are phylogenetically more related with GPAT4-8 (subclade IIIa) and GPAT6 (subclade IIIb), implying that GPAT4-6-8 are the most ancient forms of GPATs exclusive of land plants (GPAT1-8). Within subclade IIIa, most of the species presented only one sequence. The species that presented more than one sequence are *G. max*, *M. gutattus*, *B. rapa*, *A. lyrata*, *A. thaliana* (well characterized GPAT 4 and GPAT8) and *E. salsugineum.* This indicates that duplication events that originated GPAT 4 and GPAT8 were independent, lineage specific events. Subclade IIIb (GPAT6) is closely related with subclade IIIa suggesting that GPAT4, GPAT8 and GPAT6 have a common ancestral gene and diverged from duplication events. GPAT5 and GPAT7 within subclade IIId are also likely resulted from independent and lineage-specific duplication events. GPAT1, GPAT2 and GPAT3 (subclade IIIe) are closely related and may have originated by duplication events in vascular plants. The *A. thaliana* AT3G11325 gene retrieved in BLAST searches and annotated as Phospholipid/glycerol acyltransferase family protein in the Phytozome database is placed in subclade IIId, close to GPAT5 and GPAT7. This sequence also presents an acyltransferase domain.

### Comparative analysis of gene structure and organization of GPATs

**Figure 6:**
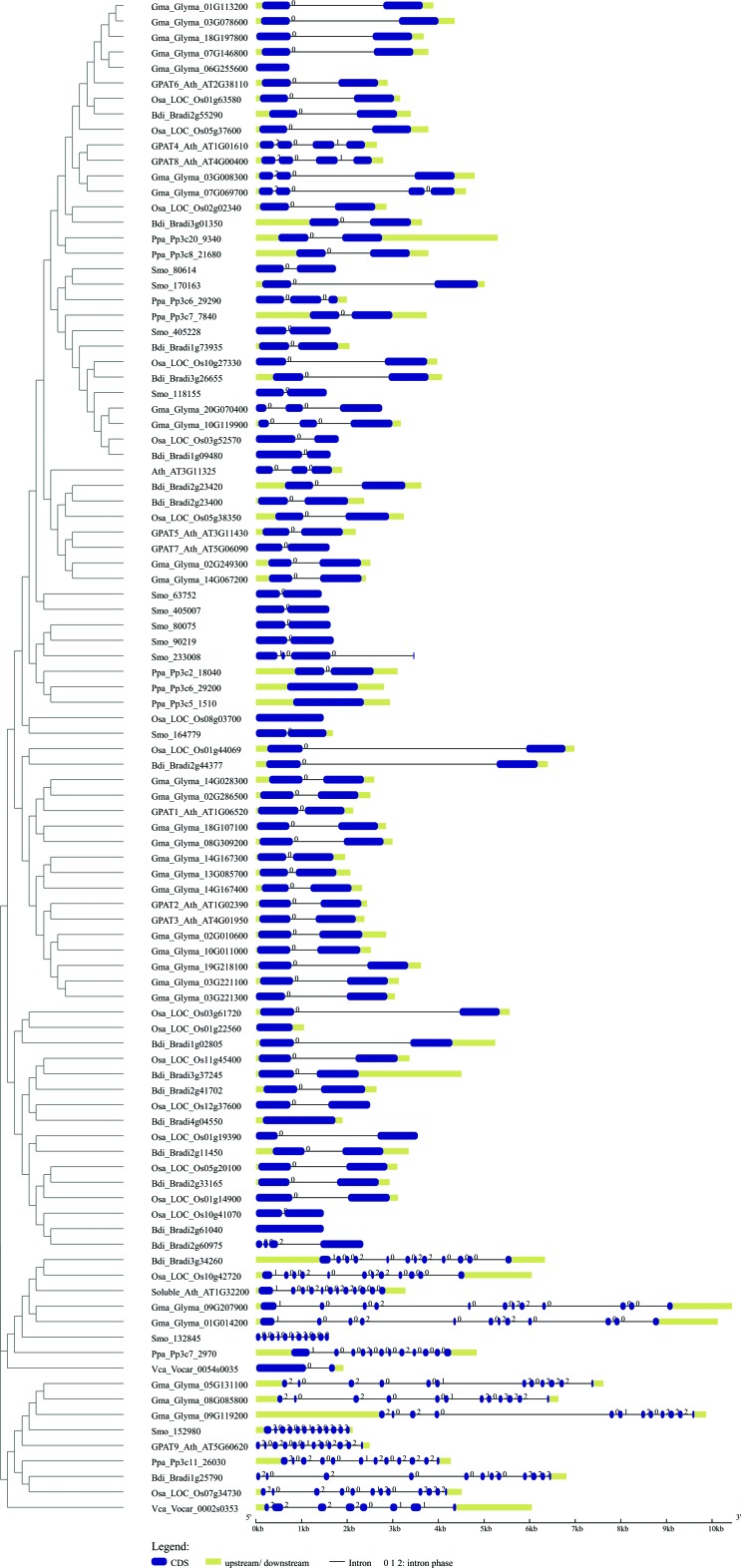
Exon-intron structure of plant and algal GPAT genes. Representative sequences of eudicots (*A. thaliana, G. max)*, monocots *(O. sativa, Z. mays)*, basal plants (*S. moellendorffii and P. patens)* and algal (*V. carteri)* are presented*.* The gene features are displayed on a phylogenetic tree reconstructed with the Neighbor Joining method. The clades I, II and II found in Figure 1 are indicated.

To explore possible mechanisms underlying gene structure and organization of GPAT genes during evolution, we compared the exon–intron organization pattern of GPAT genes from plant and algae species (Table S1 and Figure 6). The length (in base pairs) of exons and introns were counted manually by aligning the cDNA sequences to their corresponding genomic DNA sequences. These analyses revealed that the number of introns per gene ranged from zero to 14. Most of the putative GPAT sequences retrieved by BLAST searches (280) have only one intron. The number of introns and the gene organization were fairly conserved within the GPAT clades. The number of introns in algal GPAT9 genes ranged from zero to seven, while most of the GPAT9 genes from land plants have 11 introns, suggesting a possible gain of introns in land plant GPAT9 genes. The same pattern was observed for soluble GPAT. The gene structure analysis for GPAT1-8 showed that most of the species have one intron, with some exceptions, such as *A. thaliana* GPAT 4 and 8 that have three introns (Figure 6). Although several plant genes carry introns, a significant portion of plant genes lack introns. Genes that are not interrupted by introns are called intronless genes or single-exon genes. Since intronless genes are very important in understanding evolutionary patterns of related genes and genomes, we verified the intronless for GPAT genes. Twenty nine out of 450 sequences included in this study are intronless genes. Most of them belong to clade III (GPAT1-8). For GPAT9 genes, only the algal *O. lucimarinus* and *M. pusilla* GPAT9 genes are intronless.

In addition, intron phases across all GPATs of representative species (Figure 6) were investigated. The analysis showed that the intron phase pattern is quite variable across plant GPATs. Phase 0 was majority across genes with only one intron, that is the case of most GPAT1-8s. The intron phase 2,0,2,0,0,1,2,0,2,2,2 is strikingly conserved across putative GPAT9 genes that grouped into the clade I together with GPAT9 from *A. thaliana*. For the soluble GPAT the intron phase pattern is 1,0,0,2,0,0,2,2,0,0,0.

### Evaluation of GPAT protein properties

**Figure 7:**
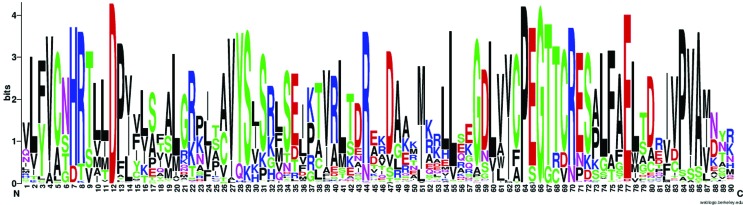
Amino acid sequence logo of the acyltransferase domain. The logo was generated from an alignment of GPAT sequences from plant and algal species. The sequences include the highly conserved motifs NHX4D (putative catalytic domain) and EGTR (putative binding domain).

After the examination of gene structure, we continued our analysis with a focus on the protein properties of 450 putative GPATs, including protein length, presence of putative transmembrane domains, and conserved motifs. Overall, the length of the GPAT amino acid sequences ranged from 237 to 621 residues (see Table S1 for details). Conserved motifs in the representative proteins from plant and algae species are depicted in Figure 7. Analysis of the amino acid sequences of the 10 members of GPATs in plants revealed that all have a plsC acyltransferase domain in the C-terminal region. A second domain in the N-terminal region that is homologs to conserved motifs of the HAD-like hydrolase superfamily is found in some GPATs (GPAT4-8). The C-terminal acyltransferase domain of the GPAT family possesses the classic H(X)4D motif of PlsC class acyltransferases (Figures 7). Predictions of transmembrane (TrM) structures showed that at least one region of GPAT1-9 proteins contained a highly probable TrM sequence (Table S3, Figure S1), while no TrM was identified for plastid GAPT, indicating that GPAT1-9 proteins are associated with membrane systems and that plastid GPAT is a soluble form.

### Expression profiling of GPAT genes in model monocot and eudicot plants

The available plant expression data from GENEVESTIGATOR was used to obtain information about potential functional roles of each GPAT. We analyzed the temporal and spatial expression patterns of the GPAT genes in plant tissues, using public microarray expression data of the eudicots *A. thaliana* (Table 2, Figures S2 and S3) and *G. max* (Table 2, Figures S4 and S5) and the monocots *O. sativa* (Table 2, Figures S6 and S7) and *Z. mays* (Table 2, Figures S8 and S9). We found probes for nine GPATs in *A. thaliana* (GPAT1-6, GPAT8, GPAT9 and soluble GPATs). For *G. max*, eight out of 28 genes identified in our BLAST searches presented available probes (Glyma.01G014200, Glyma.09G207900, Glyma.02G249300, Glyma.14G028300, Glyma.07G069700, Glyma.03G078600, Glyma.01G113200, Glyma.02G010600). For the monocots, we found 17 available probes for 17 putative GPATs for *Z. mays* (GRMZM2G165681, GRMZM2G123987, GRMZM2G065203, GRMZM2G177150, GRMZM2G147917, GRMZM2G064590, GRMZM2G124042, GRMZM2G166176, GRMZM2G083195, GRMZM2G059637, GRMZM2G072298, GRMZM2G156729, GRMZM2G070304, GRMZM2G033767, GRMZM2G020320, GRMZM2G131378, GRMZM2G159890) and 17 probes for *O. sativa* (LOC_Os01g44069, LOC_Os10g27330, LOC_Os03g52570, LOC_Os01g63580, LOC_Os05g38350, LOC_Os11g45400, LOC_Os02g02340, LOC_Os05g20100, LOC_Os08g03700, LOC_Os01g19390, LOC_Os12g37600, LOC_Os03g61720, LOC_Os01g14900, LOC_Os05g37600, LOC_Os10g41070, LOC_Os01g22560, LOC_Os07g34730). We analyzed 105 anatomical parts and 10 developmental stages from *A. thaliana*, 68 anatomical parts and five developmental stages from *G. max*, 85 anatomical parts and 7 developmental stages from *Z. may, and* 38 anatomical parts and 9 developmental stages from *O. sativa*.

**Table 2:** Microarray data analysis from Genevestigator showing expression pattern of GPATs in anatomical parts and developmental stages of *Arabidopsis thaliana*, *Glycine max, Oryza sativa* and *Zea mays.*

Species	Gene (Clade)	Anatomical parts	Development stages
*Arabidopsis thaliana*	GPAT1 - AT1G06520 (IIIe)	inflorescence, flower, stame, stigma, ovary, petal, suspensor, replum	
	GPAT2 - AT1G02390 (IIIe)	Lateral root cap protoplast, root epidermis and lateral root cap protoplast, senescent leaf	mature siliques
	GPAT3 - AT2G38110 (IIIe)	lateral root cap protoplast, root epidermis and lateral root cap protoplast, root hair cell protoplast, guard cell protoplast, guard cell	-
	GPAT4 - AT4G01950 (IIIa)	guard cell protoplast, root endodermis and quiescent center cell, root culture, seedling culture, cotyledon and leaf pavement cell, guard cell, seedling, cotyledon, pedicel	germinated seed, seedling, young rosette, developed rosette, bolting, developed flower, flowers and siliques
	GPAT5 - AT3G11430 (IIId)	root endodermis and quiescent center cell, root stele cell	-
	GPAT6 - AT1G01610 (IIIb)	flower stamen, stigma, pela, sepal	-
	GPAT7 - AT5G06090 (IIId)		-
	GPAT8 - AT4G00400 (IIIa)	guard cell protoplast, root endodermis and quiescent center cell, root culture, seedling culture, cotyledon and leaf pavement cell, trichome and leaf petiole epidermis cell, cotyledon and leaf guard cell, shoot vascular tissue and bundle sheath cell, guard cell, seedling, cotyledon	germinated seed, seedling, young rosette, developed rosette, bolting, developed flower, flowers and siliques
	GPAT9 - AT5G60620 (I)	embryo, suspensor, endosperm, micropylar endosperm peripheral endosperm chalazal endosperm, cotyledon and leaf pavement cell	senescence
	Soluble GPAT - AT1G32200 (II)	cotyledon, shoot apex, pedicel, shoot, leaf primordia, axillary shoot	germinated seed, seedling, young rosette, developed rosette, bolting, developed flower
*Glycine max*	Glyma.01G014200 (II)	shoot, trifoliolate leaf, inner integument, shoot apical meristem	lowers and siliques
	Glyma.09G207900 (II)	syncytium, paraveinal mesophyll cell palisade parenchyma cell, seedling, shoot apical meristem, axillary meristem inflorescence, embryo, suspensor, inner integument	fruit formation
	Glyma.02G249300 (IIId)	-	flowering
	Glyma.14G028300 (IIIe)	leaf	flowering
	Glyma.07G069700 (IIIa)	seedling, shoot apical meristem, axillary meristem, inflorescence, suspensor, pod, testa, shoot	-
	Glyma.03G078600 (IIIb)	pod	-
	Glyma.01G113200 (IIIb)	root hair	-
	Glyma.02G010600 (IIIe)	seedling, pod	-
*Oryza sativa*	LOC_Os01g44069/OS01G0631400 (IIIe)	pistil, stigma, ovary	-
	LOC_Os10g27330/OS10G0413400 (IIIc)	inflorescence	-
	LOC_Os03g52570/OS03G0735900 (IIIc)	inflorescence	germination
	LOC_Os01g63580/OS01G0855000 (IIIb)	inflorescence, panicle, spikelet, coleoptile, crown	germination, milk stage
	LOC_Os05g38350/OS05G0457800 (IIId)	-	-
	LOC_Os11g45400/OS11G0679700 (IIIe)	seedling, leaf, inflorescence, anther, pistil	-
	LOC_Os02g02340/OS02G0114400 (IIIa)	root	seedling, tillering stage
	LOC_Os05g20100/OS05G0280500 (IIIe)	root	-
	LOC_Os08g03700/OS08G0131300	coleoptile	-
	LOC_Os01g19390/OS01G0299300 (IIIe)	-	-
	LOC_Os12g37600/OS12G0563000 (IIIe)	coleoptile	-
	LOC_Os03g61720/OS03G0832800 (IIIe)	seedling, leaf	germination
	LOC_Os01g14900 (IIIe)	-	-
	LOC_Os05g37600/OS05G0448300 (IIIb)	-	-
	LOC_Os10g41070 (IIIe)	pollen	-
	LOC_Os01g22560/OS01G0329000 (IIIe)	sperm cell, leaf	-
	LOC_Os07g34730/OS07G0531600 (I)	sperm cell, flag leaf, collar	-
*Zea mays*	GRMZM2G165681 (I)	elongation zone, placento-chalazal region, brace root, spikelet, ovary, central starchy endosperm, conducting zone	-
	GRMZM2G123987 (I)	spikelet, central starchy endosperm, pericarp, ovary	-
	GRMZM2G065203 (IIIe)	style(silk), adult leaf, sheath, husk leaf primordium, foliar leaf primordium	-
	GRMZM2G177150 (IIIe)	husk leaf primordium	-
	GRMZM2G147917 (IIIc)	meyocite	-
	GRMZM2G064590 (IIIc)	tassel, shoot, husk leaf primordium *not high enough quantities	-
	GRMZM2G124042 (IIIc)	shoot	-
	GRMZM2G166176 (IIId)	embryo sac, adult leaf, maturation zone	-
	GRMZM2G083195 (IIIb)	husk leaf primordium, foliar leaf blade	inflorescence formation
	GRMZM2G059637 (IIId)	root, cortex, adult leaf, root tip, maturation zone	-
	GRMZM2G072298 (IIIe)	shoot	-
	GRMZM2G156729 (IIIe)	-	-
	GRMZM2G070304 (IIIe)	meyocite, pistil	-
	GRMZM2G033767 (IIIe)	sheath	-
	GRMZM2G020320 (IIIa)	adult leaf, root tip	-
	GRMZM2G131378	root tip	-
	GRMZM2G159890 (II)	foliar leaf	seedling stage, stem elongation

*In silico* analyses of the expression profiles showed that all plant GPAT genes present some expression level in developmental stages and anatomical parts. However, different expression patterns across different tissues and plant developmental stages were found across different GPATs within each species (Table 2). For example, the *A. thaliana* GPAT1 and GPAT6 genes are more expressed in inflorescence parts, while GPAT2 and GPAT3 are more expressed in root parts. The *A. thaliana* GPAT4 and GPAT8 genes presented high expression in guard cell protoplast, root endodermis and quiescent center cell, root culture, seedling culture, cotyledon and leaf pavement cell, guard cell, seedling, cotyledon, pedicel. The GPAT9 gene from *A. thaliana* is more expressed in embryo, suspensor, endosperm, micropylar endosperm, peripheral endosperm, chalazal endosperm, cotyledon and leaf pavement cell; while soluble GPAT is more expressed in cotyledon, shoot apex, pedicel, shoot, leaf primordia and axillary shoot. The *G. max* Glyma.01G113200 gene included in subclade IIIb (GPAT6) is more expressed in radicle, maturation zone and root hair, while Glyma.14G028300 (included in subclade IIIe and closely related with GPAT1 from *A. thaliana*) and Glyma.02G249300 (included in subclade IIId – GPAT5 and 7) are more expressed in flower cluster (raceme), flower, androecium, stamen, anther and pollen. *O. sativa* LOC_Os01g63580 included in the GPAT6 group (subclade IIIb) is more expressed in inflorescence, panicle, spikelet, coleoptile, anther and pistil. *O. sativa* LOC_Os01g44069 included in the GPAT1 group (subclade IIIe) is highly expressed in stigma. *O. sativa* LOC_Os11g45400 grouped into subclade IIId is more expressed in seedling, leaf, inflorescence, anther and pistil. *Z. mays* GRMZM2G070304 included in subclade IIIe (GPAT1-3) is highly expressed in spikelet cell, floret cell, stamen cell, anther cell and meyocite.

## Discussion

Glycerolipids play crucial role in plant biology, since they serve as major components of cellular membranes, storage lipids in developing seeds, and the protective hydrophobic barrier on the cuticular surface of plant organs (Ohlrogge and Browse, 1995). In addition, glycerolipids are also associated with plant growth, development and resistance to both biotic and abiotic stresses. Despite the fact that many studies have revealed key role for GPATs in glycerolipids biosynthesis, knowledge on GPATs is still limited. To advance the understanding of GPAT functions in glycerolipid biosynthesis and different physiological processes, it is essential to comprehend their evolutionary history and diversity. In this study we provide an overall picture on plant GPATs, including their gene family members, evolutionary history and gene expression profiles. On a substantial number of fully sequenced plant genomes we performed a genome-wide search and a comparative genomic analysis of the GPATs. In these analyses we included previous experimentally characterized GPATs, as well as predicted GPATs from different species. A full repertoire of genes encoding the enzymes catalyzing the first step of glycerolipid biosynthesis (450 sequences) was identified in 39 species, including algae and plants. GPAT candidate genes were found in all analyzed plants, including algae, basal plants (two mosses and one lycophyte), monocots, and eudicots. Three main clades were identified in the phylogenetic tree (Figure 1) and named clade I, clade II and clade III. Clade III is the most diversified clade and were further subdivided into five subclades (IIIa, IIIb, IIIc, IIId and IIIe).

Clade I includes GPAT9 homologs from six algae species and 31 plant species. Most species studied possess only one GPAT9 gene, and we were not able to find GPAT9 homologs in *A. trichopoda* and *P. trichocarpa*. Our phylogenetic analysis showed that GPAT9 is a very divergent clade. It has already been reported that *A. thaliana* GPAT9 is more closely related to the mammalian ER-localized GPAT3 and GPAT4 compared to other members of the *A. thaliana* GPAT family (GPAT1–8), suggesting that the divergence of the GPAT9 gene from the GPAT1–8 of this species occurred prior to the evolutionary split between plants and mammals (Gidda *et al.*, 2009) and that they have experienced different patterns of evolution. GPAT9 has been demonstrated to be involved in TAG biosynthesis and to be present in several algal species that also produce an abundance of TAGs (Khozin-Goldberg and Cohen, 2011; Iskandarov *et al.*, 2016). Heterologous expression of a GPAT9 homolog from the oleaginous green microalga *Lobosphaera incisa* in *C. reinhardtii* increased TAG content by up to 50% (Iskandarov *et al.*, 2016). In the oilseed plant *R. comunis*, GPAT9 (30122.m000357) presents higher expression compared to other GPATs in endosperm tissue, suggesting that it is likely important in castor oil synthesis (Brown *et al.*, 2012). Our expression analysis showed that *A. thaliana* GPAT9 presents higher levels of expression in embryo, suspensor, endosperm, micropylar endosperm, peripheral endosperm, chalazal endosperm, cotyledon and leaf pavement cell. Studies with *A. thaliana* showed that reduced GPAT9 expression impacts the amount and composition of TAGs in seeds (Shockey *et al.*, 2015). Another study demonstrated that GPAT9 exhibits sn-1 acyltransferase activity with high specificity for acyl-CoA, thus confirming its role in seed TAG biosynthesis, and provides comprehensive evidence in support of its role in the production of both polar and non-polar lipids in leaves, as well as lipid droplets in pollen (Singer *et al.*, 2016). The exon/intron structures (11 introns and 12 exons) and intron phase patterns (2,0,2,0,0,1,2,0,2,2,2) are conserved in almost all GPAT9 genes of land plants, which diverge form algae GPAT9 that present seven introns/six exons and intron phase patterns (2,2,2,2,0,1,1). These differences suggest that the structure of the land plant GPAT9 gene was established and retained after the divergence of land plants from algae. It also indicates an intron gain throughout Embryophyta evolution. In addition, all protein sequences grouped into clade I and classified as GPAT9 present at least one putative transmembrane domain (TMD), indicating that they are membrane proteins.

The soluble, plastid-localized GPAT homologs from six algae species and 30 plant species are grouped into clade II. Most species studied possess only one gene and we were not able to find homologs in *S. tuberosum*, *M. esculenta* and *C. sinensis*. Soluble plastid GPAT was the first GPAT to be identified in plants (Murata and Tasaka, 1997). This enzyme is essential for chloroplasts glycerolipid synthesis that are primarily converted into galactolipids, which serve as major structural and functional components of photosynthetic membranes (Dörmann and Benning, 2002). Analyses of gene expression in *A. thaliana*, *G. max* and *Z. mays* showed that soluble GPAT is predominantly expressed in green tissues, this being corroborated by the fact that this protein is involved in chloroplast lipid biosynthesis. A similar pattern was also observed for soluble GPAT of *Helianthus annuus* (HaPLSB) (Payá-Milans *et al.*, 2015). These authors demonstrated that HaPLSB expression increased during cotyledon development, which was consistent with the elevated rate of *de novo* chloroplast membrane lipid biosynthesis during the early stages of plant growth, and was maintained at high levels in mature leaves. The transmembrane domain prediction demonstrated that all sequences grouped into clade II have no TMD, confirming their soluble form for all species studied, as already was shown for *A. thaliana* (Nishida *et al.*, 1993; Kim, 2004; Chandra-Shekara *et al.*, 2007) and *H. annuus* (Payá-Milans *et al.*, 2015). The exon/intron structures (11 introns and 12 exons) and intron phase patterns (1,0,0,2,0,0,2,2,0,0,0) are conserved among land plants within clade I. The soluble GPAT from algae presents only one intron, this indicating an intron gain throughout Embryophyta evolution also for this gene.

The remaining GPATs (GPAT1-8) were included in the clade III and are present only in Embryophyta lineages. It was shown for *A. thaliana* that members of GPAT1-8 clearly affect the composition and quantity of cutin or suberin (Beisson *et al.*, 2007, 2012; Yang *et al.*, 2012). None of these GPATs seem to be required for the synthesis of membrane or storage lipids. These results demonstrated *in vivo* that a GPAT enzyme can catalyze the transfer of acyl chains to a glycerol-based acceptor and that the products can be exported to the cuticle. Suberin and cutin are extracellular lipid barriers deposited by some types of plant cells. They are essential to control gas, water, and ion fluxes, serving as physical barriers to protect plants against pathogen invasion (Kolattukudy, 2001; Schreiber, 2010; Ranathunge *et al.*, 2011). This lipid barrier is suggested be involved in the adaptation of plants to a terrestrial environment (Rensing *et al.*, 2008). Five distinct subclades were observed in GPAT phylogeny within clade III (Figure 4). The expansion and divergence of these GPATs into distinct conserved subclades (Figure 4) is associated with key stages in the morphological and functional evolution of land plants (Yang *et al.*, 2012). We observed that GPAT4 and GPAT8 in subclade IIIa resulted from an independent and lineage-specific duplication event in eudicot species. Subclade IIIa is closely related with the GPAT6 subclade. GPAT4 and GPAT8 were demonstrated to be essential for cutin formation in leaves (Li *et al.*, 2007), and GPAT6 required for cutin synthesis in flowers (Yang *et al.*, 2012). GPAT5 and GPAT7 also resulted from an independent and lineage-specific duplication event that appears to have occurred after monocot/eudicot divergence. GPAT5 has been shown to be involved in suberin synthesis in roots and seeds (Beisson *et al.*, 2007). GPAT1 is closely related with GPAT2 and GPAT3. These GPATs were shown to be located in the mitochondria. Most of the GPAT1-8 members present a conservation of intron/exon structure (one intron/2 exons). However, these GPATs presented a variable gene expression pattern, indicating that they can be differentially regulated depending on plant tissue.

In conclusion, our study provides a comprehensive genomic analysis of GPAT genes in plants, covering phylogenetic, gene structure, protein properties, and gene expression analysis. These results can improve our understanding of the evolutionary history of GPAT genes in plants and shed light on their function. Phylogenetic analysis indicates that plant GPATs can be grouped into three distinct clades, which is further supported by their conservation and variation in gene structure, protein properties, motif occurrences and gene expression patterns. Our study, together with previous studies, suggests that the presence of several genes encoding GPATs in land plants may be related to their adaptation to a terrestrial environment. Current knowledge regarding the functions of plant GPATs is limited to few species. To obtain a more thorough understanding of the function of GPATs in plants, the functional characterization of GPATs in more species will be necessary.

## Supplementary material

The following online material is available for this article:

Table S1 - Information on the GPAT sequences retrieved in this study.

Table S2 - Information about similarity with the respective Arabidopsis gene

Table S3 - Predictions of transmembrane domains of plant GPAT proteins.

Figure S1 - Properties of GPAT protein sequences of representative species.

Figure S2 - Microarray data analysis of GPATs in anatomical parts of *Arabidopsis thaliana*.

Figure S3 - Microarray data analysis of GPATs in developmental stages of *Arabidopsis thaliana*.

Figure S4 - Microarray data analysis GPATs in anatomical parts of *Glycine max*.

Figure S5 - Microarray data analysis from of GPATs in developmental stages of *Glycine max*.

Figure S6 - Microarray data analysis of GPATs in anatomical parts of *Oryza sativa*.

Figure S7 - Microarray data analysis of GPATs in developmental stages of *Oryza sativa*.

Figure S8 - Microarray data analysis of GPATs in anatomical parts of *Zea mays*.

Figure S9 - Microarray data analysis of GPATs in developmental stages of *Zea mays*.
