# Supplementary material for: Genome-wide analysis of the Glycerol-3-Phosphate Acyltransferase (GPAT) gene family reveals the evolution and diversification of plant GPATs
Source: Genet Mol Biol. 2018 Mar 19;41(1 Suppl 1):355–70. doi: 10.1590/1678-4685-GMB-2017-0076 (PMC5913721; doi:10.1590/1678-4685-GMB-2017-0076)
Supplement: Supplementary file 1 [file 1415-4757-GMB-41-01-2017-0076-s001.pdf]

Supplementary Material to "Genome-wide analysis of the Glycerol-3-Phosphate Acyltransferase (GPAT) gene family reveals the evolution and diversification of plant GPATs"

Table S1 - Species, gene name, accession numbers, protein length, protein domain information and number of introns of GPAT sequences retrieved in this study.

Species	Acronymon	Protein Acession Number	Protein Lenght	Protein Domain	nº Introns
Physcomitrella patens	Ppa	Pp3c6_29200	517	Acyltransferase (PF01553) and HAD (PF12710)	0
Physcomitrella patens	Ppa	Pp3c2_18040	529	Acyltransferase (PF01553) and HAD (PF12710)	1
Physcomitrella patens	Ppa	Pp3c7_7840	513	Acyltransferase (PF01553)	1
Physcomitrella patens	Ppa	Pp3c5_1510	516	Acyltransferase (PF01553) and HAD (PF12710)	0
Physcomitrella patens	Ppa	Pp3c20_9340	510	Acyltransferase (PF01553)	4
Physcomitrella patens	Ppa	Pp3c6_29290	501	Acyltransferase (PF01553)	2
Physcomitrella patens	Ppa	Pp3c8_21680	504	Acyltransferase (PF01553) and HAD (PF12710)	1
Physcomitrella patens	Ppa	Pp3c11_26030	389	Acyltransferase (PF01553)	11
Physcomitrella patens	Ppa	Pp3c7_2970	488	Acyltransferase (PF01553) and Glycerol-3-phosphate acyltransferase N-terminal (PF14829)	12
Sphagnum fallax	Sfa	Sphfalx0076s0073	559	Acyltransferase (PF01553) and HAD (PF12710)	2
Sphagnum fallax	Sfa	Sphfalx0011s0239	617	Acyltransferase (PF01553)	1
Sphagnum fallax	Sfa	Sphfalx0028s0065	621	Acyltransferase (PF01553) and HAD (PF12710)	1
Sphagnum fallax	Sfa	Sphfalx0077s0057	522	Acyltransferase (PF01553)	1
Sphagnum fallax	Sfa	Sphfalx0149s0036	480	Acyltransferase (PF01553)	1
Sphagnum fallax	Sfa	Sphfalx0147s0003	616	Acyltransferase (PF01553) and HAD (PF12710)	1
Sphagnum fallax	Sfa	Sphfalx0054s0107	539	Acyltransferase (PF01553)	3
Sphagnum fallax	Sfa	Sphfalx0026s0045	530	Acyltransferase (PF01553)	1
Sphagnum fallax	Sfa	Sphfalx0033s0058	276	None	1
Sphagnum fallax	Sfa	Sphfalx0016s0227	401	Acyltransferase (PF01553)	11
Sphagnum fallax	Sfa	Sphfalx0001s0271	405	Acyltransferase (PF01553)	11
Sphagnum fallax	Sfa	Sphfalx0164s0011	488	Acyltransferase (PF01553) and Glycerol-3-phosphate acyltransferase N-terminal (PF14829)	12
Selaginella moellendorffii	Smo	80075	522	Acyltransferase (PF01553)	1
Selaginella moellendorffii	Smo	118155	494	Acyltransferase (PF01553) and HAD (PF12710)	1
Selaginella moellendorffii	Smo	90219	545	Acyltransferase (PF01553)	1
Selaginella moellendorffii	Smo	405228	524	Acyltransferase (PF01553)	1
Selaginella moellendorffii	Smo	80614	490	Acyltransferase (PF01553)	1
Selaginella moellendorffii	Smo	170163	529	HAD (PF12710)	2
Selaginella moellendorffii	Smo	164779	507	Acyltransferase (PF01553)	1
Selaginella moellendorffii	Smo	63752	461	HAD (PF12710)	1
Selaginella moellendorffii	Smo	233008	480	Acyltransferase (PF01553)	3
Selaginella moellendorffii	Smo	405007	516	None	1
Selaginella moellendorffii	Smo	152980	402	Acyltransferase (PF01553)	12
Selaginella moellendorffii	Smo	132845	355	Acyltransferase (PF01553) and Glycerol-3-phosphate acyltransferase N-terminal (PF14829)	10

Species	Acronymon	Protein Acession Number	Protein Lenght	Protein Domain	nº Introns
Amborella trichopoda	Atr	evm_27.TU.AmTr_v1.0_scaffold00003.239	541	Acyltransferase (PF01553)	1
Amborella trichopoda	Atr	evm_27.TU.AmTr_v1.0_scaffold00179.5	521	Acyltransferase (PF01553)	1
Amborella trichopoda	Atr	evm_27.TU.AmTr_v1.0_scaffold00133.34	505	Acyltransferase (PF01553) and HAD (PF12710)	1
Amborella trichopoda	Atr	evm_27.TU.AmTr_v1.0_scaffold00009.182	498	Acyltransferase (PF01553) and HAD (PF12710)	1
Amborella trichopoda	Atr	evm_27.TU.AmTr_v1.0_scaffold00012.248	483	Acyltransferase (PF01553)	1
Amborella trichopoda	Atr	evm_27.TU.AmTr_v1.0_scaffold00029.356	324	Acyltransferase (PF01553)	1
Amborella trichopoda	Atr	evm_27.TU.AmTr_v1.0_scaffold00048.62	472	Acyltransferase (PF01553) and Glycerol-3-phosphate acyltransferase N-terminal (PF14829)	11
Brachypodium distachyon	Bdi	Bradi2g44377	552	Acyltransferase (PF01553)	1
Brachypodium distachyon	Bdi	Bradi1g09480	510	Acyltransferase (PF01553) and HAD (PF12710)	1
Brachypodium distachyon	Bdi	Bradi3g26655	513	Acyltransferase (PF01553)	1
Brachypodium distachyon	Bdi	Bradi2g55290	499	Acyltransferase (PF01553) and HAD (PF12710)	1
Brachypodium distachyon	Bdi	Bradi2g11450	531	Acyltransferase (PF01553)	1
Brachypodium distachyon	Bdi	Bradi1g73935	512	Acyltransferase (PF01553)	1
Brachypodium distachyon	Bdi	Bradi2g33165	532	HAD (PF12710)	1
Brachypodium distachyon	Bdi	Bradi2g23420	524	None	1
Brachypodium distachyon	Bdi	Bradi3g37245	565	Acyltransferase (PF01553)	2
Brachypodium distachyon	Bdi	Bradi3g01350	513	Acyltransferase (PF01553)	1
Brachypodium distachyon	Bdi	Bradi2g61040	495	Acyltransferase (PF01553)	0
Brachypodium distachyon	Bdi	Bradi2g41702	568	Acyltransferase (PF01553)	1
Brachypodium distachyon	Bdi	Bradi2g23400	535	None	1
Brachypodium distachyon	Bdi	Bradi4g04550	530	Acyltransferase (PF01553)	0
Brachypodium distachyon	Bdi	Bradi2g60975	454	Acyltransferase (PF01553)	3
Brachypodium distachyon	Bdi	Bradi1g02805	557	Acyltransferase (PF01553)	1
Brachypodium distachyon	Bdi	Bradi1g25790	364	Acyltransferase (PF01553)	11
Brachypodium distachyon	Bdi	Bradi3g34260	435	Acyltransferase (PF01553) and Glycerol-3-phosphate acyltransferase N-terminal (PF14829)	12
Oryza sativa	Osa	LOC_Os01g44069	544	Acyltransferase (PF01553)	1
Oryza sativa	Osa	LOC_Os10g27330	530	Acyltransferase (PF01553)	1
Oryza sativa	Osa	LOC_Os03g52570	467	Acyltransferase (PF01553)	1
Oryza sativa	Osa	LOC_Os01g63580	497	Acyltransferase (PF01553) and HAD (PF12710)	1
Oryza sativa	Osa	LOC_Os05g38350	522	HAD (PF12710)	1
Oryza sativa	Osa	LOC_Os11g45400	543	Acyltransferase (PF01553)	1
Oryza sativa	Osa	LOC_Os02g02340	506	Acyltransferase (PF01553)	1
Oryza sativa	Osa	LOC_Os05g20100	537	Acyltransferase (PF01553)	1
Oryza sativa	Osa	LOC_Os08g03700	495	Acyltransferase (PF01553)	0
Oryza sativa	Osa	LOC_Os01g19390	453	Acyltransferase (PF01553)	1
Oryza sativa	Osa	LOC_Os12g37600	558	Acyltransferase (PF01553)	1
Oryza sativa	Osa	LOC_Os03g61720	541	Acyltransferase (PF01553)	1
Oryza sativa	Osa	LOC_Os01g14900	570	Acyltransferase (PF01553)	1
Oryza sativa	Osa	LOC_Os05g37600	486	Acyltransferase (PF01553) and HAD (PF12710)	1

Species	Acronymon	Protein Acession Number	Protein Lenght	Protein Domain	nº Introns
Oryza sativa	Osa	LOC_Os10g41070	479	Acyltransferase (PF01553)	1
Oryza sativa	Osa	LOC_Os01g22560	264	Acyltransferase (PF01553)	0
Oryza sativa	Osa	LOC_Os07g34730	370	Acyltransferase (PF01553)	11
Oryza sativa	Osa	LOC_Os10g42720	427	Acyltransferase (PF01553) and Glycerol-3-phosphate acyltransferase N-terminal (PF14829)	13
Panicum hallii	Pha	Pahal.E02373	543	Acyltransferase (PF01553)	1
Panicum hallii	Pha	Pahal.I01831	547	Acyltransferase (PF01553) and HAD (PF12710)	1
Panicum hallii	Pha	Pahal.B03381	506	Acyltransferase (PF01553)	1
Panicum hallii	Pha	Pahal.E00896.	499	Acyltransferase (PF01553)	1
Panicum hallii	Pha	Pahal.I00545	506	Acyltransferase (PF01553)	1
Panicum hallii	Pha	Pahal.E03181	539	Acyltransferase (PF01553)	1
Panicum hallii	Pha	Pahal.C02741	514	HAD (PF12710)	1
Panicum hallii	Pha	Pahal.I04570	547	Acyltransferase (PF01553)	1
Panicum hallii	Pha	Pahal.H00181	566	Acyltransferase (PF01553)	1
Panicum hallii	Pha	Pahal.E03539	533	None	1
Panicum hallii	Pha	Pahal.C03674	537	None	1
Panicum hallii	Pha	Pahal.I01321	536	None	1
Panicum hallii	Pha	Pahal.A00044	520	Acyltransferase (PF01553)	1
Panicum hallii	Pha	Pahal.F02713	493	Acyltransferase (PF01553)	0
Panicum hallii	Pha	Pahal.I04042	487	Acyltransferase (PF01553)	0
Panicum hallii	Pha	Pahal.C02792	494	HAD (PF12710)	1
Panicum hallii	Pha	Pahal.B04165	423	Acyltransferase (PF01553)	11
Panicum hallii	Pha	Pahal.I03757	455	Acyltransferase (PF01553) and Glycerol-3-phosphate acyltransferase N-terminal (PF14829)	13
Setaria italica	Sit	Seita.9G101700	512	Acyltransferase (PF01553) and HAD (PF12710)	1
Setaria italica	Sit	Seita.9G261200	509	Acyltransferase (PF01553) and HAD (PF12710)	1
Setaria italica	Sit	Seita.5G386400	498	Acyltransferase (PF01553)	1
Setaria italica	Sit	Seita.9G527800	507	Acyltransferase (PF01553)	2
Setaria italica	Sit	Seita.4G285600	502	Acyltransferase (PF01553)	1
Setaria italica	Sit	Seita.3G291600	536	Acyltransferase (PF01553)	1
Setaria italica	Sit	Seita.3G218600	517	HAD (PF12710)	1
Setaria italica	Sit	Seita.5G028700	538	Acyltransferase (PF01553)	1
Setaria italica	Sit	Seita.3G223100	495	Acyltransferase (PF01553)	1
Setaria italica	Sit	Seita.5G060100	541	Acyltransferase (PF01553)	1
Setaria italica	Sit	Seita.8G234000	560	Acyltransferase (PF01553)	1
Setaria italica	Sit	Seita.9G023200	537	None	1
Setaria italica	Sit	Seita.3G334400	580	Acyltransferase (PF01553)	1
Setaria italica	Sit	Seita.1G116700	528	Acyltransferase (PF01553)	1
Setaria italica	Sit	Seita.6G074300	498	Acyltransferase (PF01553)	0
Setaria italica	Sit	Seita.3G219000	498	Acyltransferase (PF01553)	0
Setaria italica	Sit	Seita.9G527900	438	HAD (PF12710)	3

Species	Acronymon	Protein Acession Number	Protein Lenght	Protein Domain	nº Introns
Setaria italica	Sit	Seita.9G323800	493	Acyltransferase (PF01553)	0
Setaria italica	Sit	Seita.2G339000	371	Acyltransferase (PF01553)	11
Setaria italica	Sit	Seita.9G307400	449	Acyltransferase (PF01553) and Glycerol-3-phosphate acyltransferase N-terminal (PF14829)	13
Setaria viridis	Svi	Sevir.5G243800	551	Acyltransferase (PF01553)	1
Setaria viridis	Svi	Sevir.9G099900	512	Acyltransferase (PF01553) and HAD (PF12710)	1
Setaria viridis	Svi	Sevir.9G264100	509	Acyltransferase (PF01553) and HAD (PF12710)	1
Setaria viridis	Svi	Sevir.5G391500	498	Acyltransferase (PF01553)	1
Setaria viridis	Svi	Sevir.9G532600	507	Acyltransferase (PF01553)	2
Setaria viridis	Svi	Sevir.3G300000	536	Acyltransferase (PF01553)	1
Setaria viridis	Svi	Sevir.4G298000	502	Acyltransferase (PF01553)	1
Setaria viridis	Svi	Sevir.5G027400	538	Acyltransferase (PF01553)	1
Setaria viridis	Svi	Sevir.9G022800	531	HAD (PF12710)	1
Setaria viridis	Svi	Sevir.3G228100	495	Acyltransferase (PF01553)	1
Setaria viridis	Svi	Sevir.5G059700	541	Acyltransferase (PF01553)	1
Setaria viridis	Svi	Sevir.8G244100	560	Acyltransferase (PF01553)	1
Setaria viridis	Svi	Sevir.3G348800	580	Acyltransferase (PF01553)	1
Setaria viridis	Svi	Sevir.1G115700	528	Acyltransferase (PF01553)	1
Setaria viridis	Svi	Sevir.6G073400	498	Acyltransferase (PF01553)	0
Setaria viridis	Svi	Sevir.3G224100	498	Acyltransferase (PF01553)	0
Setaria viridis	Svi	Sevir.9G329700	493	Acyltransferase (PF01553)	0
Setaria viridis	Svi	Sevir.2G349100	371	Acyltransferase (PF01553)	11
Setaria viridis	Svi	Sevir.9G312800	449	Acyltransferase (PF01553) and Glycerol-3-phosphate acyltransferase N-terminal (PF14829)	13
Sorghum bicolor	Sbi	Sobic.003G229700	572	Acyltransferase (PF01553)	1
Sorghum bicolor	Sbi	Sobic.001G099300	505	Acyltransferase (PF01553) and HAD (PF12710)	2
Sorghum bicolor	Sbi	Sobic.001G250200	518	Acyltransferase (PF01553)	1
Sorghum bicolor	Sbi	Sobic.001G493300	524	Acyltransferase (PF01553)	1
Sorghum bicolor	Sbi	Sobic.003G360700	503	Acyltransferase (PF01553)	1
Sorghum bicolor	Sbi	Sobic.009G162000	522	HAD (PF12710)	1
Sorghum bicolor	Sbi	Sobic.004G010300	518	Acyltransferase (PF01553)	1
Sorghum bicolor	Sbi	Sobic.003G114200	555	None	1
Sorghum bicolor	Sbi	Sobic.003G142500	537	Acyltransferase (PF01553)	1
Sorghum bicolor	Sbi	Sobic.008G130800	603	Acyltransferase (PF01553)	1
Sorghum bicolor	Sbi	Sobic.001G026100	569	Acyltransferase (PF01553)	1
Sorghum bicolor	Sbi	Sobic.005G214400	581	Acyltransferase (PF01553)	1
Sorghum bicolor	Sbi	Sobic.009G202600	497	Acyltransferase (PF01553)	0
Sorghum bicolor	Sbi	Sobic.002G325300	371	Acyltransferase (PF01553)	11
Sorghum bicolor	Sbi	Sobic.001G283700	448	Acyltransferase (PF01553) and Glycerol-3-phosphate acyltransferase N-terminal (PF14829)	13
Zea mays	Zma	GRMZM2G165681	371	Acyltransferase (PF01553)	11
Zea mays	Zma	GRMZM2G123987	371	Acyltransferase (PF01553)	11

Species	Acronymon	Protein Acession Number	Protein Lenght	Protein Domain	nº Introns
Zea mays	Zma	GRMZM2G065203	545	Acyltransferase (PF01553)	1
Zea mays	Zma	GRMZM2G177150	557	Acyltransferase (PF01553)	1
Zea mays	Zma	GRMZM2G147917	511	Acyltransferase (PF01553) and HAD (PF12710)	1
Zea mays	Zma	GRMZM2G064590	521	Acyltransferase (PF01553)	1
Zea mays	Zma	GRMZM2G124042	506	Acyltransferase (PF01553)	1
Zea mays	Zma	GRMZM2G166176	498	Acyltransferase (PF01553)	1
Zea mays	Zma	GRMZM2G083195	502	Acyltransferase (PF01553)	1
Zea mays	Zma	GRMZM2G059637	512	HAD (PF12710)	1
Zea mays	Zma	GRMZM2G072298	542	None	1
Zea mays	Zma	GRMZM2G156729	537	Acyltransferase (PF01553)	1
Zea mays	Zma	GRMZM2G070304	525	Acyltransferase (PF01553)	1
Zea mays	Zma	GRMZM2G033767	537	Acyltransferase (PF01553)	1
Zea mays	Zma	GRMZM2G020320	515	Acyltransferase (PF01553)	0
Zea mays	Zma	GRMZM2G131378	496	Acyltransferase (PF01553)	0
Zea mays	Zma	GRMZM2G159890	447	Acyltransferase (PF01553) and Glycerol-3-phosphate acyltransferase N-terminal (PF14829)	11
Aquilegia coerulea	Aco	Aquca_057_00060	565	Acyltransferase (PF01553)	1
Aquilegia coerulea	Aco	Aquca_003_00476	524	Acyltransferase (PF01553)	1
Aquilegia coerulea	Aco	Aquca_045_00017	496	Acyltransferase (PF01553)	1
Aquilegia coerulea	Aco	Aquca_029_00122	540	Acyltransferase (PF01553)	1
Aquilegia coerulea	Aco	Aquca_004_00312	495	Acyltransferase (PF01553)	1
Aquilegia coerulea	Aco	Aquca_025_00147	509	Acyltransferase (PF01553) and HAD (PF12710)	1
Aquilegia coerulea	Aco	Aquca_045_00015	482	Acyltransferase (PF01553)	1
Aquilegia coerulea	Aco	Aquca_004_00505	499	Acyltransferase (PF01553)	1
Aquilegia coerulea	Aco	Aquca_010_00671	477	Acyltransferase (PF01553)	1
Aquilegia coerulea	Aco	Aquca_013_00284	499	Acyltransferase (PF01553)	1
Aquilegia coerulea	Aco	Aquca_009_00928	490	Acyltransferase (PF01553)	1
Aquilegia coerulea	Aco	Aquca_009_00929	461	Acyltransferase (PF01553)	1
Aquilegia coerulea	Aco	Aquca_001_00233	361	Acyltransferase (PF01553)	11
Aquilegia coerulea	Aco	Aquca_047_00038	362	Acyltransferase (PF01553)	11
Aquilegia coerulea	Aco	Aqcoe3G171100	376	Acyltransferase (PF01553) and Glycerol-3-phosphate acyltransferase N-terminal (PF14829)	13
Mimulus guttatus	Mgu	Migut.M00497	547	Acyltransferase (PF01553)	1
Mimulus guttatus	Mgu	Migut.M00495	518	Acyltransferase (PF01553)	2
Mimulus guttatus	Mgu	Migut.J00189	492	Acyltransferase (PF01553)	1
Mimulus guttatus	Mgu	Migut.L01365	506	Acyltransferase (PF01553) and HAD (PF12710)	1
Mimulus guttatus	Mgu	Migut.H00880	502	Acyltransferase (PF01553) and HAD (PF12710)	1
Mimulus guttatus	Mgu	Migut.N01447	504	Acyltransferase (PF01553)	2
Mimulus guttatus	Mgu	Migut.F00761	503	Acyltransferase (PF01553)	1
Mimulus guttatus	Mgu	Migut.B01763	507	Acyltransferase (PF01553) and HAD (PF12710)	1
Mimulus guttatus	Mgu	Migut.G00542	504	Acyltransferase (PF01553)	1

**Species**	**Acronymon**	**Protein Acession Number**	**Protein Lenght**	**Protein Domain**	**nº Introns**
Mimulus guttatus	Mgu	Migut.G00541	495	Acyltransferase (PF01553)	1
Mimulus guttatus	Mgu	Migut.O00899	358	Acyltransferase (PF01553)	1
Mimulus guttatus	Mgu	Migut.N01350	370	Acyltransferase (PF01553)	11
Mimulus guttatus	Mgu	Migut.E00743	458	Acyltransferase (PF01553) and Glycerol-3-phosphate acyltransferase N-terminal (PF14829)	12
Solanum lycopersicum	Sly	Solyc07g056320.2	546	Acyltransferase (PF01553)	2
Solanum lycopersicum	Sly	Solyc02g087500.1	508	Acyltransferase (PF01553)	1
Solanum lycopersicum	Sly	Solyc04g005840.1	499	Acyltransferase (PF01553) and HAD (PF12710)	1
Solanum lycopersicum	Sly	Solyc09g014350.2.	504	Acyltransferase (PF01553) and HAD (PF12710)	1
Solanum lycopersicum	Sly	Solyc01g094700.2	501	Acyltransferase (PF01553)	1
Solanum lycopersicum	Sly	Solyc04g011600.2	491	Acyltransferase (PF01553)	2
Solanum lycopersicum	Sly	Solyc05g053030.1	502	None	1
Solanum lycopersicum	Sly	Solyc10g084900.1	486	Acyltransferase (PF01553) and HAD (PF12710)	0
Solanum lycopersicum	Sly	Solyc08g082340.2	371	Acyltransferase (PF01553)	11
Solanum lycopersicum	Sly	Solyc08g076470.2	470	Acyltransferase (PF01553) and Glycerol-3-phosphate acyltransferase N-terminal (PF14829)	13
Solanum tuberosum	Stu	PGSC0003DMG400012284	371	Acyltransferase (PF01553)	11
Solanum tuberosum	Stu	PGSC0003DMG400017301	544	Acyltransferase (PF01553)	2
Solanum tuberosum	Stu	PGSC0003DMG400002838	502	Acyltransferase (PF01553) and HAD (PF12710)	1
Solanum tuberosum	Stu	PGSC0003DMG400040955	508	Acyltransferase (PF01553)	1
Solanum tuberosum	Stu	PGSC0003DMG400001404	480	Acyltransferase (PF01553)	1
Solanum tuberosum	Stu	PGSC0003DMG400000046	501	Acyltransferase (PF01553)	1
Solanum tuberosum	Stu	PGSC0003DMG400020315	506	Acyltransferase (PF01553) and HAD (PF12710)	1
Solanum tuberosum	Stu	PGSC0003DMG400011080	493	Acyltransferase (PF01553) and HAD (PF12710)	0
Solanum tuberosum	Stu	PGSC0003DMG400011079	494	Acyltransferase (PF01553)	0
Solanum tuberosum	Stu	PGSC0003DMG400006342	396	Acyltransferase (PF01553)	2
Solanum tuberosum	Stu	PGSC0003DMG400011077	489	Acyltransferase (PF01553)	0
Solanum tuberosum	Stu	PGSC0003DMG400028523	302	Acyltransferase (PF01553)	0
Solanum tuberosum	Stu	PGSC0003DMT400032021	371	Acyltransferase (PF01553)	10
Eucalyptus grandis	Egr	Eucgr.A01977	549	Acyltransferase (PF01553)	1
Eucalyptus grandis	Egr	Eucgr.F04388	517	Acyltransferase (PF01553)	1
Eucalyptus grandis	Egr	Eucgr.B03397	503	Acyltransferase (PF01553) and HAD (PF12710)	1
Eucalyptus grandis	Egr	Eucgr.K03558	554	Acyltransferase (PF01553)	1
Eucalyptus grandis	Egr	Eucgr.F04389	457	Acyltransferase (PF01553)	1
Eucalyptus grandis	Egr	Eucgr.A00515	509	Acyltransferase (PF01553)	1
Eucalyptus grandis	Egr	Eucgr.E00121	503	Acyltransferase (PF01553)	2
Eucalyptus grandis	Egr	Eucgr.J01235	489	Acyltransferase (PF01553)	1
Eucalyptus grandis	Egr	Eucgr.G02949	519	HAD (PF12710)	1
Eucalyptus grandis	Egr	Eucgr.I01507	375	Acyltransferase (PF01553)	11
Eucalyptus grandis	Egr	Eucgr.E00228	401	Acyltransferase (PF01553) and Glycerol-3-phosphate acyltransferase N-terminal (PF14829)	12
Eucalyptus grandis	Egr	Eucgr.E01634	285	Acyltransferase (PF01553)	8

Species	Acronymon	Protein Acession Number	Protein Lenght	Protein Domain	nº Introns
Manihot esculenta	Mes	Manes.02G084800	532	Acyltransferase (PF01553)	1
Manihot esculenta	Mes	Manes.01G127800	539	Acyltransferase (PF01553)	1
Manihot esculenta	Mes	Manes.02G157300	514	Acyltransferase (PF01553)	1
Manihot esculenta	Mes	Manes.01G230400	546	Acyltransferase (PF01553)	1
Manihot esculenta	Mes	Manes.05G012900	546	Acyltransferase (PF01553)	1
Manihot esculenta	Mes	Manes.01G193000	501	Acyltransferase (PF01553) and HAD (PF12710)	1
Manihot esculenta	Mes	Manes.09G103500	505	Acyltransferase (PF01553) and HAD (PF12710)	1
Manihot esculenta	Mes	Manes.08G089900	503	Acyltransferase (PF01553)	1
Manihot esculenta	Mes	Manes.07G118400	491	Acyltransferase (PF01553)	1
Manihot esculenta	Mes	Manes.01G255900	500	Acyltransferase (PF01553)	2
Manihot esculenta	Mes	Manes.11G106900	375	Acyltransferase (PF01553)	11
Populus trichocarpa	Ptr	Potri.005G202200	542	Acyltransferase (PF01553)	1
Populus trichocarpa	Ptr	Potri.002G192600	552	Acyltransferase (PF01553)	1
Populus trichocarpa	Ptr	Potri.016G063900	501	Acyltransferase (PF01553) and HAD (PF12710)	1
Populus trichocarpa	Ptr	Potri.006G198100	501	Acyltransferase (PF01553) and HAD (PF12710)	1
Populus trichocarpa	Ptr	Potri.016G113100	500	Acyltransferase (PF01553) and HAD (PF12710)	1
Populus trichocarpa	Ptr	Potri.006G097800	497	Acyltransferase (PF01553) and HAD (PF12710)	1
Populus trichocarpa	Ptr	Potri.010G201200	488	Acyltransferase (PF01553)	1
Populus trichocarpa	Ptr	Potri.008G058200	489	Acyltransferase (PF01553)	1
Populus trichocarpa	Ptr	Potri.014G085500	500	Acyltransferase (PF01553)	2
Populus trichocarpa	Ptr	Potri.001G136600	453	Acyltransferase (PF01553) and Glycerol-3-phosphate acyltransferase N-terminal (PF14829)	12
Ricinus communis	Rco	30122.t000017	360	Acyltransferase (PF01553)	10
Ricinus communis	Rco	28350.t000007	537	Acyltransferase (PF01553)	1
Ricinus communis	Rco	29736.t000066	504	Acyltransferase (PF01553) and HAD (PF12710)	1
Ricinus communis	Rco	29822.t000116	512	Acyltransferase (PF01553) and HAD (PF12710)	1
Ricinus communis	Rco	29908.t000018	543	Acyltransferase (PF01553)	1
Ricinus communis	Rco	30076.t000182	509	Acyltransferase (PF01553)	3
Ricinus communis	Rco	30174.t000012	501	Acyltransferase (PF01553) and HAD (PF12710)	2
Ricinus communis	Rco	27568.t000015	490	Acyltransferase (PF01553)	1
Ricinus communis	Rco	29969.t000007	293	Acyltransferase (PF01553)	1
Ricinus communis	Rco	30068.t000150	455	Acyltransferase (PF01553) and Glycerol-3-phosphate acyltransferase N-terminal (PF14829)	11
Citrus sinensis	Csi	orange1.1g009120m.g	543	Acyltransferase (PF01553)	1
Citrus sinensis	Csi	orange1.1g009762m.g	526	Acyltransferase (PF01553)	1
Citrus sinensis	Csi	orange1.1g042288m.g	515	Acyltransferase (PF01553) and HAD (PF12710)	1
Citrus sinensis	Csi	orange1.1g042170m.g	543	Acyltransferase (PF01553)	1
Citrus sinensis	Csi	orange1.1g043920m.g	504	Acyltransferase (PF01553)	1
Citrus sinensis	Csi	orange1.1g010860m.g	499	Acyltransferase (PF01553)	2
Citrus sinensis	Csi	orange1.1g018906m.g	349	Acyltransferase (PF01553)	2
Citrus sinensis	Csi	orange1.1g038704m.g	237	Acyltransferase (PF01553)	0

Species	Acronymon	Protein Acession Number	Protein Lenght	Protein Domain	nº Introns
Citrus sinensis	Csi	orange1.1g017205m	375	Acyltransferase (PF01553)	11
Citrus clementina	Ccl	Ciclev10028672m.g	375	Acyltransferase (PF01553)	11
Citrus clementina	Ccl	Ciclev10031163m.g	543	Acyltransferase (PF01553)	1
Citrus clementina	Ccl	Ciclev10001145m.g	447	Acyltransferase (PF01553)	1
Citrus clementina	Ccl	Ciclev10013880m.g	515	Acyltransferase (PF01553) and HAD (PF12710)	1
Citrus clementina	Ccl	Ciclev10023483m.g	543	Acyltransferase (PF01553)	1
Citrus clementina	Ccl	Ciclev10033948m.g	504	Acyltransferase (PF01553)	1
Citrus clementina	Ccl	Ciclev10011536m.g	503	Acyltransferase (PF01553) and HAD (PF12710)	1
Citrus clementina	Ccl	Ciclev10031344m.g	490	Acyltransferase (PF01553)	1
Citrus clementina	Ccl	Ciclev10019825m.g	499	Acyltransferase (PF01553)	2
Citrus clementia	Ccl	Ciclev10001047m.g	469	Acyltransferase (PF01553) and Glycerol-3-phosphate acyltransferase N-terminal (PF14829)	12
Gossypium raimondii	Gra	Gorai.012G158300	378	Acyltransferase (PF01553)	11
Gossypium raimondii	Gra	Gorai.005G258500	377	Acyltransferase (PF01553)	11
Gossypium raimondii	Gra	Gorai.008G098000	541	Acyltransferase (PF01553)	1
Gossypium raimondii	Gra	Gorai.006G181200	504	Acyltransferase (PF01553) and HAD (PF12710)	1
Gossypium raimondii	Gra	Gorai.010G045500	516	Acyltransferase (PF01553)	1
Gossypium raimondii	Gra	Gorai.007G078200	510	Acyltransferase (PF01553) and HAD (PF12710)	1
Gossypium raimondii	Gra	Gorai.004G145100	543	Acyltransferase (PF01553)	1
Gossypium raimondii	Gra	Gorai.012G112800	515	Acyltransferase (PF01553)	1
Gossypium raimondii	Gra	Gorai.011G151300	504	Acyltransferase (PF01553) and HAD (PF12710)	1
Gossypium raimondii	Gra	Gorai.004G235900	538	Acyltransferase (PF01553)	1
Gossypium raimondii	Gra	Gorai.011G267100	494	Acyltransferase (PF01553)	1
Gossypium raimondii	Gra	Gorai.012G071300	488	Acyltransferase (PF01553)	1
Gossypium raimondii	Gra	Gorai.007G100100	500	Acyltransferase (PF01553)	2
Gossypium raimondii	Gra	Gorai.011G267200	405	Acyltransferase (PF01553)	3
Gossypium raimondii	Gra	Gorai.007G033500	440	Acyltransferase (PF01553) and Glycerol-3-phosphate acyltransferase N-terminal (PF14829)	11
Gossypium raimondii	Gra	Gorai.004G182200	446	Acyltransferase (PF01553) and Glycerol-3-phosphate acyltransferase N-terminal (PF14829)	12
Gossypium raimondii	Gra	Gorai.003G185500	461	Acyltransferase (PF01553) and Glycerol-3-phosphate acyltransferase N-terminal (PF14829)	12
Theobroma cacao	Tca	Thecc1EG006479	416	Acyltransferase (PF01553)	12
Theobroma cacao	Tca	Thecc1EG001873	540	Acyltransferase (PF01553)	1
Theobroma cacao	Tca	Thecc1EG034986	553	Acyltransferase (PF01553)	2
Theobroma cacao	Tca	Thecc1EG034985	522	Acyltransferase (PF01553)	1
Theobroma cacao	Tca	Thecc1EG004855	537	Acyltransferase (PF01553)	1
Theobroma cacao	Tca	Thecc1EG021737	503	Acyltransferase (PF01553) and HAD (PF12710)	1
Theobroma cacao	Tca	Thecc1EG018071	507	Acyltransferase (PF01553) and HAD (PF12710)	1
Theobroma cacao	Tca	Thecc1EG026783	531	Acyltransferase (PF01553)	1
Theobroma cacao	Tca	Thecc1EG010070	593	Acyltransferase (PF01553) and HAD (PF12710)	3
Theobroma cacao	Tca	Thecc1EG042716	488	Acyltransferase (PF01553)	2
Theobroma cacao	Tca	Thecc1EG005317	500	Acyltransferase (PF01553)	3

Species	Acronymon	Protein Acession Number	Protein Lenght	Protein Domain	nº Introns
Theobroma cacao	Tca	Thecc1EG016600	461	Acyltransferase (PF01553) and Glycerol-3-phosphate acyltransferase N-terminal (PF14829)	13
Arabidopsis lyrata	Aly	919369	584	Acyltransferase (PF01553)	1
Arabidopsis lyrata	Aly	470198	525	Acyltransferase (PF01553)	1
Arabidopsis lyrata	Aly	943550	520	Acyltransferase (PF01553)	1
Arabidopsis lyrata	Aly	478480	502	Acyltransferase (PF01553)	4
Arabidopsis lyrata	Aly	482818	501	Acyltransferase (PF01553)	2
Arabidopsis lyrata	Aly	918798	502	Acyltransferase (PF01553)	3
Arabidopsis lyrata	Aly	490527	498	Acyltransferase (PF01553)	4
Arabidopsis lyrata	Aly	349873	499	Acyltransferase (PF01553)	1
Arabidopsis lyrata	Aly	950810	376	Acyltransferase (PF01553)	11
Arabidopsis lyrata	Aly	AL1G46330	459	Acyltransferase (PF01553) and Glycerol-3-phosphate acyltransferase N-terminal (PF14829)	12
Arabidopsis thaliana	Ath	AT1G06520	585	Acyltransferase (PF01553)	1
Arabidopsis thaliana	Ath	AT1G02390	530	Acyltransferase (PF01553)	1
Arabidopsis thaliana	Ath	AT2G38110	501	Acyltransferase (PF01553) and HAD (PF12710)	1
Arabidopsis thaliana	Ath	AT4G01950	520	Acyltransferase (PF01553)	1
Arabidopsis thaliana	Ath	AT3G11430	502	Acyltransferase (PF01553) and HAD (PF12710)	1
Arabidopsis thaliana	Ath	AT1G01610	503	Acyltransferase (PF01553) and HAD (PF12710)	3
Arabidopsis thaliana	Ath	AT5G06090	500	Acyltransferase (PF01553) and HAD (PF12710)	1
Arabidopsis thaliana	Ath	AT4G00400	500	Acyltransferase (PF01553) and HAD (PF12710)	3
Arabidopsis thaliana	Ath	AT5G60620	376	Acyltransferase (PF01553)	11
Arabidopsis thaliana	Ath	AT1G32200	459	Acyltransferase (PF01553) and Glycerol-3-phosphate acyltransferase N-terminal (PF14829)	12
Arabidopsis thaliana	Ath	AT3G11325	376	None	2
Brassica rapa	Bra	Brara.A00307	376	Acyltransferase (PF01553)	11
Brassica rapa	Bra	Brara.J01416	368	Acyltransferase (PF01553)	11
Brassica rapa	Bra	Brara.J00449	571	Acyltransferase (PF01553)	1
Brassica rapa	Bra	Brara.K01852	500	Acyltransferase (PF01553)	3
Brassica rapa	Bra	Brara.A03408	502	Acyltransferase (PF01553)	1
Brassica rapa	Bra	Brara.I00118	531	Acyltransferase (PF01553)	1
Brassica rapa	Bra	Brara.C02835	520	Acyltransferase (PF01553)	1
Brassica rapa	Bra	Brara.I05638	501	Acyltransferase (PF01553)	2
Brassica rapa	Bra	Brara.E02936	500	Acyltransferase (PF01553)	1
Brassica rapa	Bra	Brara.D02328	501	Acyltransferase (PF01553)	1
Brassica rapa	Bra	Brara.J00035	503	Acyltransferase (PF01553)	2
Brassica rapa	Bra	Brara.E00709	501	Acyltransferase (PF01553)	1
Brassica rapa	Bra	Brara.J02607	498	Acyltransferase (PF01553)	1
Brassica rapa	Bra	Brara.E02888	458	Acyltransferase (PF01553)	2
Brassica rapa	Bra	Brara.H00582	454	Acyltransferase (PF01553) and Glycerol-3-phosphate acyltransferase N-terminal (PF14829)	12
Brassica rapa	Bra	Brara.E01731	440	Acyltransferase (PF01553) and Glycerol-3-phosphate acyltransferase N-terminal (PF14829)	12
Brassica rapa	Bra	Brara.I02607	443	Acyltransferase (PF01553) and Glycerol-3-phosphate acyltransferase N-terminal (PF14829)	12

Species	Acronymon	Protein Acession Number	Protein Lenght	Protein Domain	nº Introns
Capsella grandiflora	Cgr	Cagra.1671s0280	577	Acyltransferase (PF01553)	1
Capsella grandiflora	Cgr	Cagra.1968s0023	529	Acyltransferase (PF01553)	1
Capsella grandiflora	Cgr	Cagra.0334s0034	521	Acyltransferase (PF01553)	1
Capsella grandiflora	Cgr	Cagra.1655s0027	503	Acyltransferase (PF01553)	1
Capsella grandiflora	Cgr	Cagra.1968s0101	502	Acyltransferase (PF01553)	3
Capsella grandiflora	Cgr	Cagra.2117s0053	500	Acyltransferase (PF01553)	1
Capsella grandiflora	Cgr	Cagra.1261s0029	502	Acyltransferase (PF01553)	3
Capsella grandiflora	Cgr	Cagra.1655s0038	499	Acyltransferase (PF01553)	1
Capsella grandiflora	Cgr	Cagra.15555s0001	289	Acyltransferase (PF01553)	0
Capsella grandiflora	Cgr	Cagra.2519s0033	377	Acyltransferase (PF01553)	11
Capsella grandiflora	Cgr	Cagra.3957s0001	460	Acyltransferase (PF01553) and Glycerol-3-phosphate acyltransferase N-terminal (PF14829)	11
Capsella rubella	Cru	Carubv10026601m.g	377	Acyltransferase (PF01553)	11
Capsella rubella	Cru	Carubv10012249m	577	Acyltransferase (PF01553)	1
Capsella rubella	Cru	Carubv10008716m	568	Acyltransferase (PF01553)	1
Capsella rubella	Cru	Carubv10000657m	540	Acyltransferase (PF01553)	1
Capsella rubella	Cru	Carubv10013500m	503	Acyltransferase (PF01553)	1
Capsella rubella	Cru	Carubv10023060m	502	Acyltransferase (PF01553)	1
Capsella rubella	Cru	Carubv10008926m	502	Acyltransferase (PF01553)	3
Capsella rubella	Cru	Carubv10000788m	500	Acyltransferase (PF01553)	1
Capsella rubella	Cru	Carubv10000780m	502	Acyltransferase (PF01553)	3
Capsella rubella	Cru	Carubv10015640m	499	Acyltransferase (PF01553)	1
Capsella rubella	Cru	Carubv10012611m.g	459	Acyltransferase (PF01553) and Glycerol-3-phosphate acyltransferase N-terminal (PF14829)	11
Eutrema salsugineum	Esa	Thhalv10007168m	586	Acyltransferase (PF01553)	1
Eutrema salsugineum	Esa	Thhalv10020574m	500	Acyltransferase (PF01553)	1
Eutrema salsugineum	Esa	Thhalv10007347m	529	Acyltransferase (PF01553)	1
Eutrema salsugineum	Esa	Thhalv10028577m	521	Acyltransferase (PF01553)	1
Eutrema salsugineum	Esa	Thhalv10007431m	502	Acyltransferase (PF01553)	3
Eutrema salsugineum	Esa	Thhalv10016551m	501	Acyltransferase (PF01553)	1
Eutrema salsugineum	Esa	Thhalv10028601m	499	Acyltransferase (PF01553)	3
Eutrema salsugineum	Esa	Thhalv10015521m	500	Acyltransferase (PF01553)	1
Eutrema salsugineum	Esa	Thhalv10013846m.g	376	Acyltransferase (PF01553)	11
Eutrema salsugineum	Esa	Thhalv10007540m.g	471	Acyltransferase (PF01553) and Glycerol-3-phosphate acyltransferase N-terminal (PF14829)	12
Cucumis sativus	Csa	Cucsa.099550	375	Acyltransferase (PF01553)	10
Cucumis sativus	Csa	Cucsa.185470	496	Acyltransferase (PF01553)	3
Cucumis sativus	Csa	Cucsa.339200	511	Acyltransferase (PF01553) and HAD (PF12710)	1
Cucumis sativus	Csa	Cucsa.176200	505	Acyltransferase (PF01553)	1
Cucumis sativus	Csa	Cucsa.165180	447	Acyltransferase (PF01553)	1
Cucumis sativus	Csa	Cucsa.135570	500	Acyltransferase (PF01553)	2
Cucumis sativus	Csa	Cucsa.204890	542	Acyltransferase (PF01553)	1

**Species**	**Acronymon**	**Protein Acession Number**	**Protein Lenght**	**Protein Domain**	**nº Introns**
Cucumis sativus	Csa	Cucsa.312830	470	Acyltransferase (PF01553) and Glycerol-3-phosphate acyltransferase N-terminal (PF14829)	12
Glycine max	Gma	Glyma.14G028300	540	Acyltransferase (PF01553)	1
Glycine max	Gma	Glyma.02G286500	539	Acyltransferase (PF01553)	1
Glycine max	Gma	Glyma.18G107100	527	Acyltransferase (PF01553)	1
Glycine max	Gma	Glyma.08G309200	533	Acyltransferase (PF01553)	1
Glycine max	Gma	Glyma.14G167300	505	Acyltransferase (PF01553)	1
Glycine max	Gma	Glyma.13G085700	503	Acyltransferase (PF01553)	1
Glycine max	Gma	Glyma.03G221100	539	Acyltransferase (PF01553)	1
Glycine max	Gma	Glyma.19G218100	540	Acyltransferase (PF01553)	1
Glycine max	Gma	Glyma.02G010600	555	Acyltransferase (PF01553)	1
Glycine max	Gma	Glyma.07G146800	496	Acyltransferase (PF01553) and HAD (PF12710)	1
Glycine max	Gma	Glyma.14G167400	497	Acyltransferase (PF01553)	1
Glycine max	Gma	Glyma.03G221300	509	Acyltransferase (PF01553)	1
Glycine max	Gma	Glyma.10G011000.	556	Acyltransferase (PF01553)	1
Glycine max	Gma	Glyma.18G197800	539	Acyltransferase (PF01553) and HAD (PF12710)	1
Glycine max	Gma	Glyma.01G113200	492	Acyltransferase (PF01553)	1
Glycine max	Gma	Glyma.03G078600	498	Acyltransferase (PF01553) and HAD (PF12710)	1
Glycine max	Gma	Glyma.20G070400	512	Acyltransferase (PF01553)	2
Glycine max	Gma	Glyma.10G119900	509	Acyltransferase (PF01553)	2
Glycine max	Gma	Glyma.02G249300	472	Acyltransferase (PF01553)	1
Glycine max	Gma	Glyma.03G008300	500	Acyltransferase (PF01553)	2
Glycine max	Gma	Glyma.07G069700	499	Acyltransferase (PF01553)	3
Glycine max	Gma	Glyma.14G067200	472	Acyltransferase (PF01553)	1
Glycine max	Gma	Glyma.06G255600	278	Acyltransferase (PF01553)	0
Glycine max	Gma	Glyma.09G119200	376	Acyltransferase (PF01553)	13
Glycine max	Gma	Glyma.08G085800	373	Acyltransferase (PF01553)	12
Glycine max	Gma	Glyma.05G131100	373	Acyltransferase (PF01553)	12
Glycine max	Gma	Glyma.01G014200	458	Acyltransferase (PF01553) and Glycerol-3-phosphate acyltransferase N-terminal (PF14829)	12
Glycine max	Gma	Glyma.09G207900	470	Acyltransferase (PF01553) and Glycerol-3-phosphate acyltransferase N-terminal (PF14829)	12
Medicago truncatula	Mtr	Medtr4g127910	376	Acyltransferase (PF01553)	11
Medicago truncatula	Mtr	Medtr2g438210	364	Acyltransferase (PF01553)	11
Medicago truncatula	Mtr	Medtr5g091660	539	Acyltransferase (PF01553)	1
Medicago truncatula	Mtr	Medtr3g448430	511	Acyltransferase (PF01553)	1
Medicago truncatula	Mtr	Medtr5g061520	489	Acyltransferase (PF01553)	1
Medicago truncatula	Mtr	Medtr4g415290	495	Acyltransferase (PF01553) and HAD (PF12710)	1
Medicago truncatula	Mtr	Medtr7g067380	498	Acyltransferase (PF01553) and HAD (PF12710)	1
Medicago truncatula	Mtr	Medtr1g040500	568	Acyltransferase (PF01553) and HAD (PF12710)	2
Medicago truncatula	Mtr	Medtr8g030620	505	Acyltransferase (PF01553)	2
Medicago truncatula	Mtr	Medtr1g086650	542	Acyltransferase (PF01553)	1

**Species**	**Acronymon**	**Protein Acession Number**	**Protein Lenght**	**Protein Domain**	**nº Introns**
Medicago truncatula	Mtr	Medtr5g080360	493	Acyltransferase (PF01553)	1
Medicago truncatula	Mtr	Medtr5g029230	457	Acyltransferase (PF01553) and Glycerol-3-phosphate acyltransferase N-terminal (PF14829)	12
Phaseolus vulgaris	Pvu	Phvul.006G033100	532	Acyltransferase (PF01553)	1
Phaseolus vulgaris	Pvu	Phvul.008G191600	366	Acyltransferase (PF01553)	1
Phaseolus vulgaris	Pvu	Phvul.008G169500	501	Acyltransferase (PF01553)	1
Phaseolus vulgaris	Pvu	Phvul.008G106700	496	Acyltransferase (PF01553) and HAD (PF12710)	1
Phaseolus vulgaris	Pvu	Phvul.008G169400	505	Acyltransferase (PF01553)	1
Phaseolus vulgaris	Pvu	Phvul.007G233600	512	Acyltransferase (PF01553) and HAD (PF12710)	2
Phaseolus vulgaris	Pvu	Phvul.007G212600	540	Acyltransferase (PF01553)	1
Phaseolus vulgaris	Pvu	Phvul.010G099700	501	Acyltransferase (PF01553) and HAD (PF12710)	2
Phaseolus vulgaris	Pvu	Phvul.L005200	493	Acyltransferase (PF01553)	1
Phaseolus vulgaris	Pvu	Phvul.003G022900	376	Acyltransferase (PF01553)	14
Phaseolus vulgaris	Pvu	Phvul.002G191600	325	Acyltransferase (PF01553)	12
Phaseolus vulgaris	Pvu	Phvul.002G136600	461	Acyltransferase (PF01553) and Glycerol-3-phosphate acyltransferase N-terminal (PF14829)	12
Chlamydomonas reinhardtii	Cre	Cre06.g273250	456	Acyltransferase (PF01553)	8
Chlamydomonas reinhardtii	Cre	Cre02.g143000	410	Acyltransferase (PF01553) and Glycerol-3-phosphate acyltransferase N-terminal (PF14829)	1
Volvox carteri	Vca	Vocar.0002s0353	435	Acyltransferase (PF01553)	7
Volvox carteri	Vca	Vocar.0054s0035	406	Acyltransferase (PF01553) and Glycerol-3-phosphate acyltransferase N-terminal (PF14829)	1
Coccomyxa subellipsoidea	Csu	gw1.3.285.1	313	Acyltransferase (PF01553)	4
Coccomyxa subellipsoidea	Csu	estExt_Genewise1Plus.C_210008	429	Acyltransferase (PF01553) and Glycerol-3-phosphate acyltransferase N-terminal (PF14829)	5
Micromonas pusilla	Mpu	MicpuC2.estExt_Genewise1Plus.C_160166	343	Acyltransferase (PF01553)	0
Micromonas pusilla	Mpu	e_gw1.8.756.1	452	Acyltransferase (PF01553)	2
Micromonas sp.	Msp	estExt_Genewise2Plus.C_Chr_050706	389	Acyltransferase (PF01553)	1
Micromonas sp.	Msp	estExt_fgenesh2_pg.C_Chr_110323	430	Acyltransferase (PF01553)	1
Ostreococcus lucimarinus	Olu	estExt_Genewise_ext.C_Chr_160282	380	Acyltransferase (PF01553)	0
Otreococcus lucimarinus	Olu	eugene.0200010222	429	Acyltransferase (PF01553)	0
Otreococcus lucimarinus	Olu	eugene1.1.0200012220	429	Acyltransferase (PF01553)	0
