# Supplementary material for: Genome-wide analysis of the Glycerol-3-Phosphate Acyltransferase (GPAT) gene family reveals the evolution and diversification of plant GPATs
Source: Genet Mol Biol. 2018 Mar 19;41(1 Suppl 1):355–70. doi: 10.1590/1678-4685-GMB-2017-0076 (PMC5913721; doi:10.1590/1678-4685-GMB-2017-0076)
Supplement: Supplementary file 2 [file 1415-4757-GMB-41-01-2017-0076-s002.pdf]

Supplementary Material to "Genome-wide analysis of the Glycerol-3-Phosphate Acyltransferase (GPAT) gene family reveals the evolution and diversification of plant GPATs"

Table S2 - Information about clade (I, II or III) of the respective protein, the more similar Arabidopsis gene, the percentage of similarity with the Arabidopsis gene, and cellular localization.

**Species**	**Protein Accession Number**	**Clade**	**The more similar Arabidopsis gene**	**Similarity (%)**	**Cellular localization**
Physcomitrella patens	Pp3c6_29200	III	GPAT6	69.4	ER
Physcomitrella patens	Pp3c2_18040	III	GPAT6	64.8	ER
Physcomitrella patens	Pp3c7_7840	III	GPAT6	72.1	ER
Physcomitrella patens	Pp3c5_1510	III	GPAT6	68.8	ER
Physcomitrella patens	Pp3c20_9340	III	GPAT6	72.0	ER
Physcomitrella patens	Pp3c6_29290	III	GPAT6	73.5	ER
Physcomitrella patens	Pp3c8_21680	III	GPAT6	71.0	ER
Physcomitrella patens	Pp3c11_26030	I	GPAT9	80.2	ER
Physcomitrella patens	Pp3c7_2970	II	Soluble GPAT	55.9	ER
Sphagnum fallax	Sphfalx0076s0073	III	GPAT6	62.6	ER
Sphagnum fallax	Sphfalx0011s0239	III	GPAT6	60.0	ER
Sphagnum fallax	Sphfalx0028s0065	III	GPAT4	56.0	ER
Sphagnum fallax	Sphfalx0077s0057	III	GPAT6	69.0	ER
Sphagnum fallax	Sphfalx0149s0036	III	GPAT4	67.3	ER
Sphagnum fallax	Sphfalx0147s0003	III	GPAT4	55.8	ER
Sphagnum fallax	Sphfalx0054s0107	III	GPAT6	65.3	ER
Sphagnum fallax	Sphfalx0026s0045	III	GPAT6	65.7	ER
Sphagnum fallax	Sphfalx0033s0058	III	GPAT6	64.5	ER
Sphagnum fallax	Sphfalx0016s0227	I	GPAT9	77.6	ER
Sphagnum fallax	Sphfalx0001s0271	I	GPAT9	76.3	ER
Sphagnum fallax	Sphfalx0164s0011	II	Soluble GPAT	56.6	Chloroplast
Selaginella moellendorffii	80075	III	GPAT6	61.3	ER
Selaginella moellendorffii	118155	III	GPAT6	66.6	ER
Selaginella moellendorffii	90219	III	GPAT6	56.5	ER
Selaginella moellendorffii	405228	III	GPAT6	61.8	ER
Selaginella moellendorffii	80614	III	GPAT6	72.4	ER
Selaginella moellendorffii	170163	III	GPAT6	69.6	ER
Selaginella moellendorffii	164779	III	GPAT6, GPAT8	54.8	ER
Selaginella moellendorffii	63752	III	GPAT8	62.9	ER
Selaginella moellendorffii	233008	III	GPAT4	60.0	ER
Selaginella moellendorffii	405007	III	GPAT2	56.6	Mitochondria
Selaginella moellendorffii	152980	I	GPAT9	78.9	ER
Selaginella moellendorffii	132845	II	Soluble GPAT	76.3	Chloroplast
Amborella trichopoda	evm_27.TU.AmTr_v1.0_scaffold00003.239	III	GPAT2	65.6	Mitochondria
Amborella trichopoda	evm_27.TU.AmTr_v1.0_scaffold00179.5	III	GPAT1	65.1	Mitochondria
Amborella trichopoda	evm_27.TU.AmTr_v1.0_scaffold00133.34	III	GPAT8	66.7	ER
Amborella trichopoda	evm_27.TU.AmTr_v1.0_scaffold00009.182	III	GPAT6	78.5	ER
Amborella trichopoda	evm_27.TU.AmTr_v1.0_scaffold00012.248	III	GPAT5	72.5	ER
Amborella trichopoda	evm_27.TU.AmTr_v1.0_scaffold00029.356	III	GPAT6	74.1	ER
Amborella trichopoda	evm_27.TU.AmTr_v1.0_scaffold00048.62	II	Soluble GPAT	68.0	Chloroplast
Brachypodium distachyon	Bradi2g44377	III	GPAT1	59.4	Mitochondria
Brachypodium distachyon	Bradi1g09480	III	GPAT4	67.6	ER
Brachypodium distachyon	Bradi3g26655	III	GPAT6	60.8	ER
Brachypodium distachyon	Bradi2g55290	III	GPAT6	82.8	ER

Species	Protein Accession Number	Clade	The more similar Arabidopsis gene	Similarity (%)	Cellular localization
Brachypodium distachyon	Bradi2g11450	III	GPAT2	58.9	Mitochondria
Brachypodium distachyon	Bradi1g73935	III	GPAT8	60.7	ER
Brachypodium distachyon	Bradi2g33165	III	GPAT2	54.9	Mitochondria
Brachypodium distachyon	Bradi2g23420	III	GPAT5	56.1	ER
Brachypodium distachyon	Bradi3g37245	III	GPAT2	52.2	Mitochondria
Brachypodium distachyon	Bradi3g01350	III	GPAT4	63.2	ER
Brachypodium distachyon	Bradi2g61040	III	GPAT1	52.3	Mitochondria
Brachypodium distachyon	Bradi2g41702	III	GPAT2	53.0	ER
Brachypodium distachyon	Bradi2g23400	III	GPAT5	55.0	ER
Brachypodium distachyon	Bradi4g04550	III	GPAT2	50.4	ER
Brachypodium distachyon	Bradi2g60975	III	GPAT2	52.6	ER
Brachypodium distachyon	Bradi1g02805	III	GPAT2	46.3	ER
Brachypodium distachyon	Bradi1g25790	I	GPAT9	87.6	ER
Brachypodium distachyon	Bradi3g34260	II	Soluble GPAT	72.1	Chloroplast
Oryza sativa	LOC_Os01g44069	III	GPAT1	60.1	Mitochondria
Oryza sativa	LOC_Os10g27330	III	GPAT6	56.6	ER
Oryza sativa	LOC_Os03g52570	III	GPAT6	60.6	ER
Oryza sativa	LOC_Os01g63580	III	GPAT6	84.1	ER
Oryza sativa	LOC_Os05g38350	III	GPAT6	84.1	ER
Oryza sativa	LOC_Os11g45400	III	GPAT2	57.6	Mitochondria
Oryza sativa	LOC_Os02g02340	III	GPAT4	66.4	ER
Oryza sativa	LOC_Os05g20100	III	GPAT2	53.8	Mitochondria
Oryza sativa	LOC_Os08g03700	III	GPAT8	54.5	ER
Oryza sativa	LOC_Os01g19390	III	GPAT2	57.6	Mitochondria
Oryza sativa	LOC_Os12g37600	III	GPAT2	46.2	Mitochondria
Oryza sativa	LOC_Os03g61720	III	GPAT2	51.0	Mitochondria
Oryza sativa	LOC_Os01g14900	III	GPAT2	45.8	Mitochondria
Oryza sativa	LOC_Os05g37600	III	GPAT6	73.5	ER
Oryza sativa	LOC_Os10g41070	III	GPAT1	47.6	Mitochondria
Oryza sativa	LOC_Os01g22560	III	GPAT1	58.8	Mitochondria
Oryza sativa	LOC_Os07g34730	I	GPAT9	87.0	ER
Oryza sativa	LOC_Os10g42720	II	Soluble GPAT	71.7	Chloroplast
Panicum hallii	Pahal.E02373	III	GPAT1	57.6	Mitochondria
Panicum hallii	Pahal.I01831	III	GPAT8	62.0	ER
Panicum hallii	Pahal.B03381	III	GPAT6	67.4	ER
Panicum hallii	Pahal.E00896.	III	GPAT6	83.2	ER
Panicum hallii	Pahal.I00545	III	GPAT6	62.3	ER
Panicum hallii	Pahal.E03181	III	GPAT2	59.2	Mitochondria
Panicum hallii	Pahal.C02741	III	GPAT5	62.2	ER
Panicum hallii	Pahal.I04570	III	GPAT1	50.1	Mitochondria
Panicum hallii	Pahal.H00181	III	GPAT2	53.4	Mitochondria
Panicum hallii	Pahal.E03539	III	GPAT1	50.3	Mitochondria
Panicum hallii	Pahal.C03674	III	GPAT1	52.3	Mitochondria
Panicum hallii	Pahal.I01321	III	GPAT1	46.3	Mitochondria
Panicum hallii	Pahal.A00044	III	GPAT4	62.1	ER
Panicum hallii	Pahal.F02713	III	GPAT1	55.5	Mitochondria
Panicum hallii	Pahal.I04042	III	GPAT1	54.8	Mitochondria
Panicum hallii	Pahal.C02792	III	GPAT6	65.6	ER
Panicum hallii	Pahal.B04165	I	GPAT9	76.1	ER
Panicum hallii	Pahal.I03757	II	Soluble GPAT	64.0	Chloroplast
Setaria italica	Seita.9G101700	III	GPAT8	67.0	ER
Setaria italica	Seita.9G261200	III	GPAT6, GPAT4	67.0	ER
Setaria italica	Seita.5G386400	III	GPAT6	84.1	ER
Setaria italica	Seita.9G527800	III	GPAT6, GPAT4	59.8	ER
Setaria italica	Seita.4G285600	III	GPAT6, GPAT4	60.6	ER
Setaria italica	Seita.3G291600	III	GPAT2	54.7	Mitochondria

Species	Protein Accession Number	Clade	The more similar Arabidopsis gene	Similarity (%)	Cellular localization
Setaria italica	Seita.3G218600	III	GPAT5	62.3	ER
Setaria italica	Seita.5G028700	III	GPAT2	65.1	ER
Setaria italica	Seita.3G223100	III	GPAT6	72.5	ER
Setaria italica	Seita.5G060100	III	GPAT1	51.0	ER
Setaria italica	Seita.8G234000	III	GPAT2	51.6	Mitochondria
Setaria italica	Seita.9G023200	III	GPAT1	48.8	Mitochondria
Setaria italica	Seita.3G334400	III	GPAT2	48.6	Mitochondria
Setaria italica	Seita.1G116700	III	GPAT4	62.3	ER
Setaria italica	Seita.6G074300	III	GPAT4	56.0	ER
Setaria italica	Seita.3G219000	III	GPAT1	54.6	Mitochondria
Setaria italica	Seita.9G527900	III	GPAT8	62.1	ER
Setaria italica	Seita.9G323800	III	GPAT2	55.0	Mitochondria
Setaria italica	Seita.2G339000	I	GPAT9	86.5	ER
Setaria italica	Seita.9G307400	II	Soluble GPAT	65.7	Chloroplast
Setaria viridis	Sevir.5G243800	III	GPAT1	59.9	Mitochondria
Setaria viridis	Sevir.9G099900	III	GPAT8	66.8	ER
Setaria viridis	Sevir.9G264100	III	GPAT8	66.2	ER
Setaria viridis	Sevir.5G391500	III	GPAT6	84.1	ER
Setaria viridis	Sevir.9G532600	III	GPAT4, GPAT6	59.4	ER
Setaria viridis	Sevir.3G300000	III	GPAT2	54.7	Mitochondria
Setaria viridis	Sevir.4G298000	III	GPAT6	60.6	ER
Setaria viridis	Sevir.5G027400	III	GPAT2	64.3	Mitochondria
Setaria viridis	Sevir.9G022800	III	GPAT1	49.0	Mitochondria
Setaria viridis	Sevir.3G228100	III	GPAT6	72.5	ER
Setaria viridis	Sevir.5G059700	III	GPAT1	51.0	Mitochondria
Setaria viridis	Sevir.8G244100	III	GPAT2	51.4	Mitochondria
Setaria viridis	Sevir.3G348800	III	GPAT2	48.4	Mitochondria
Setaria viridis	Sevir.1G115700	III	GPAT4	63.6	ER
Setaria viridis	Sevir.6G073400	III	GPAT4	57.8	ER
Setaria viridis	Sevir.3G224100	III	GPAT1	54.6	Mitochondria
Setaria viridis	Sevir.9G329700	III	GPAT2	55.0	Mitochondria
Setaria viridis	Sevir.2G349100	I	GPAT9	86.5	ER
Setaria viridis	Sevir.9G312800	II	Soluble GPAT	65.7	Chloroplast
Sorghum bicolor	Sobic.003G229700	III	GPAT1	60.8	Mitochondria
Sorghum bicolor	Sobic.001G099300	III	GPAT6	71.5	ER
Sorghum bicolor	Sobic.001G250200	III	GPAT6	67.0	ER
Sorghum bicolor	Sobic.001G493300	III	GPAT6	65.3	ER
Sorghum bicolor	Sobic.003G360700	III	GPAT6	85.7	ER
Sorghum bicolor	Sobic.009G162000	III	GPAT5	72.0	ER
Sorghum bicolor	Sobic.004G010300	III	GPAT4	71.2	ER
Sorghum bicolor	Sobic.003G114200	III	GPAT2	60.2	Mitochondria
Sorghum bicolor	Sobic.003G142500	III	GPAT3	65.0	Mitochondria
Sorghum bicolor	Sobic.008G130800	III	GPAT3	52.6	Mitochondria
Sorghum bicolor	Sobic.001G026100	III	GPAT2	56.9	Mitochondria
Sorghum bicolor	Sobic.005G214400	III	GPAT3	57.8	Mitochondria
Sorghum bicolor	Sobic.009G202600	III	GPAT2	56.5	Mitochondria
Sorghum bicolor	Sobic.002G325300	I	GPAT9	87.3	ER
Sorghum bicolor	Sobic.001G283700	II	Soluble GPAT	66.7	Chloroplast
Zea mays	GRMZM2G165681	I	GPAT9	86.8	ER
Zea mays	GRMZM2G123987	I	GPAT9	86.8	ER
Zea mays	GRMZM2G065203	III	GPAT1	65.9	Mitochondria
Zea mays	GRMZM2G177150	III	GPAT1	60.3	Mitochondria
Zea mays	GRMZM2G147917	III	GPAT4, GPAT8	66.3	ER
Zea mays	GRMZM2G064590	III	GPAT6	64.7	ER
Zea mays	GRMZM2G124042	III	GPAT6	65.8	ER
Zea mays	GRMZM2G166176	III	GPAT5	59.0	ER

**Species**	**Protein Accession Number**	**Clade**	**The more similar Arabidopsis gene**	**Similarity (%)**	**Cellular localization**
Zea mays	GRMZM2G083195	III	GPAT6	81.9	ER
Zea mays	GRMZM2G059637	III	GPAT7	61.7	ER
Zea mays	GRMZM2G072298	III	GPAT1	56.1	Mitochondria
Zea mays	GRMZM2G156729	III	GPAT2	47.3	Mitochondria
Zea mays	GRMZM2G070304	III	GPAT2	55.8	Mitochondria
Zea mays	GRMZM2G033767	III	GPAT2	46.2	Mitochondria
Zea mays	GRMZM2G020320	III	GPAT4	60.0	ER
Zea mays	GRMZM2G131378	III	GPAT1	55.4	Mitochondria
Zea mays	GRMZM2G159890	II	Soluble GPAT	66.4	Chloroplast
Aquilegia coerulea	Aquca_057_00060	III	GPAT1	71.3	Mitochondria
Aquilegia coerulea	Aquca_003_00476	III	GPAT2	74.1	Mitochondria
Aquilegia coerulea	Aquca_045_00017	III	GPAT6	86.7	ER
Aquilegia coerulea	Aquca_029_00122	III	GPAT2	66.9	Mitochondria
Aquilegia coerulea	Aquca_004_00312	III	GPAT6	86.7	ER
Aquilegia coerulea	Aquca_025_00147	III	GPAT6	68.8	ER
Aquilegia coerulea	Aquca_045_00015	III	GPAT6	81.3	ER
Aquilegia coerulea	Aquca_004_00505	III	GPAT4	61.7	ER
Aquilegia coerulea	Aquca_010_00671	III	GPAT5	71.3	ER
Aquilegia coerulea	Aquca_013_00284	III	GPAT4	85.2	ER
Aquilegia coerulea	Aquca_009_00928	III	GPAT5	76.4	ER
Aquilegia coerulea	Aquca_009_00929	III	GPAT7	74.5	ER
Aquilegia coerulea	Aquca_001_00233	I	GPAT9	92.5	ER
Aquilegia coerulea	Aquca_047_00038	I	GPAT9	86.7	ER
Aquilegia coerulea	Aqcoe3G171100	II	Soluble GPAT	70.2	Chloroplast
Mimulus guttatus	Migut.M00497	III	GPAT1	70.9	Mitochondria
Mimulus guttatus	Migut.M00495	III	GPAT1	69.1	Mitochondria
Mimulus guttatus	Migut.J00189	III	GPAT6	89.6	ER
Mimulus guttatus	Migut.L01365	III	GPAT8	70.2	ER
Mimulus guttatus	Migut.H00880	III	GPAT4	68.7	ER
Mimulus guttatus	Migut.N01447	III	GPAT8	82.1	ER
Mimulus guttatus	Migut.F00761	III	GPAT5	70.6	ER
Mimulus guttatus	Migut.B01763	III	GPAT4	83.0	ER
Mimulus guttatus	Migut.G00542	III	GPAT6	64.1	ER
Mimulus guttatus	Migut.G00541	III	GPAT4, GPAT8	63.8	ER
Mimulus guttatus	Migut.O00899	III	GPAT6	66.2	ER
Mimulus guttatus	Migut.N01350	I	GPAT9	92.4	ER
Mimulus guttatus	Migut.E00743	II	Soluble GPAT	67.2	Chloroplast
Solanum lycopersicum	Solyc07g056320.2	III	GPAT1	77.3	Mitochondria
Solanum lycopersicum	Solyc02g087500.1	III	GPAT6	72.8	ER
Solanum lycopersicum	Solyc04g005840.1	III	GPAT6	74.3	ER
Solanum lycopersicum	Solyc09g014350.2.	III	GPAT6	87.9	ER
Solanum lycopersicum	Solyc01g094700.2	III	GPAT4	88.6	ER
Solanum lycopersicum	Solyc04g011600.2	III	GPAT5	84.9	ER
Solanum lycopersicum	Solyc05g053030.1	III	GPAT5	77.1	ER
Solanum lycopersicum	Solyc10g084900.1	III	GPAT6	69.3	ER
Solanum lycopersicum	Solyc08g082340.2	I	GPAT9	93.0	ER
Solanum lycopersicum	Solyc08g076470.2	II	Soluble GPAT	71.7	Chloroplast
Solanum tuberosum	PGSC0003DMT400044571	III	GPAT1	77.9	Mitochondria
Solanum tuberosum	PGSC0003DMT400007387	III	GPAT6	73.9	ER
Solanum tuberosum	PGSC0003DMT400091384	III	GPAT6	72.0	ER
Solanum tuberosum	PGSC0003DMT400003553	III	GPAT6	72.7	ER
Solanum tuberosum	PGSC0003DMT400000152	III	GPAT4	88.6	ER
Solanum tuberosum	PGSC0003DMT400052335	III	GPAT6	87.4	ER
Solanum tuberosum	PGSC0003DMT400028782	III	GPAT6	71.8	ER
Solanum tuberosum	PGSC0003DMT400028781	III	GPAT6	71.7	ER
Solanum tuberosum	PGSC0003DMT400016230	III	GPAT5	86.1	ER

**Species**	**Protein Accession Number**	**Clade**	**The more similar Arabidopsis gene**	**Similarity (%)**	**Cellular localization**
Solanum tuberosum	PGSC0003DMT400028775	III	GPAT6	67.7	ER
Solanum tuberosum	PGSC0003DMT400073428	III	GPAT5	74.2	ER
Solanum tuberosum	PGSC0003DMT400069796	III	Soluble GPAT	45.8	Chloroplast
Solanum tuberosum	PGSC0003DMT400032021	I	GPAT9	92.7	ER
Eucalyptus grandis	Eucgr.A01977	III	GPAT1	76.7	Mitochondria
Eucalyptus grandis	Eucgr.F04388	III	GPAT2	67.3	Mitochondria
Eucalyptus grandis	Eucgr.B03397	III	GPAT8	68.6	ER
Eucalyptus grandis	Eucgr.K03558	III	GPAT2	68.1	Mitochondria
Eucalyptus grandis	Eucgr.F04389	III	GPAT2	71.3	Mitochondria
Eucalyptus grandis	Eucgr.A00515	III	GPAT6	83.5	ER
Eucalyptus grandis	Eucgr.E00121	III	GPAT8	88.3	ER
Eucalyptus grandis	Eucgr.J01235	III	GPAT7	75.1	ER
Eucalyptus grandis	Eucgr.G02949	III	GPAT7	69.9	ER
Eucalyptus grandis	Eucgr.I01507	I	GPAT9	88.0	ER
Eucalyptus grandis	Eucgr.E00228	II	Soluble GPAT	80.0	Chloroplast
Eucalyptus grandis	Eucgr.E01634	II	Soluble GPAT	72.6	Chloroplast
Manihot esculenta	Manes.02G084800	III	GPAT1	81.0	Mitochondria
Manihot esculenta	Manes.01G127800	III	GPAT1	76.4	Mitochondria
Manihot esculenta	Manes.02G157300	III	GPAT2	73.9	Mitochondria
Manihot esculenta	Manes.01G230400	III	GPAT2	71.4	Mitochondria
Manihot esculenta	Manes.05G012900	III	GPAT2	70.3	Mitochondria
Manihot esculenta	Manes.01G193000	III	GPAT8	70.5	ER
Manihot esculenta	Manes.09G103500	III	GPAT6	87.3	ER
Manihot esculenta	Manes.08G089900	III	GPAT6	87.1	ER
Manihot esculenta	Manes.07G118400	III	GPAT5	78.6	ER
Manihot esculenta	Manes.01G255900	III	GPAT4	90.0	ER
Manihot esculenta	Manes.11G106900	I	GPAT9	93.1	ER
Populus trichocarpa	Potri.005G202200	III	GPAT1	75.5	Mitochondria
Populus trichocarpa	Potri.002G192600	III	GPAT2	67.4	Mitochondria
Populus trichocarpa	Potri.016G063900	III	GPAT4	70.9	ER
Populus trichocarpa	Potri.006G198100	III	GPAT4	70.9	ER
Populus trichocarpa	Potri.016G113100	III	GPAT6	86.4	ER
Populus trichocarpa	Potri.006G097800	III	GPAT6	89.5	ER
Populus trichocarpa	Potri.010G201200	III	GPAT5	82.0	ER
Populus trichocarpa	Potri.008G058200	III	GPAT5	80.0	ER
Populus trichocarpa	Potri.014G085500	III	GPAT8	84.0	ER
Populus trichocarpa	Potri.001G136600	II	Soluble GPAT	74.2	Chloroplast
Ricinus communis	30122.m000357	I	GPAT9	94.2	ER
Ricinus communis	28350.m000105	III	GPAT1	78.0	Mitochondria
Ricinus communis	29736.m002070	III	GPAT4	70.4	ER
Ricinus communis	29822.m003441	III	GPAT8	65.4	ER
Ricinus communis	29908.m005967	III	GPAT2	65.2	Mitochondria
Ricinus communis	30076.m004618	III	GPAT2	64.4	Mitochondria
Ricinus communis	30174.m008615	III	GPAT8	90.0	ER
Ricinus communis	27568.m000266	III	GPAT5	81.2	ER
Ricinus communis	29969.m000267	III	GPAT6	90.8	ER
Ricinus communis	30068.m002660	II	Soluble GPAT	68.8	Chloroplast
Citrus sinensis	orange1.1g009120m.g	III	GPAT1	77.3	Mitochondria
Citrus sinensis	orange1.1g009762m.g	III	GPAT2	71.7	Mitochondria
Citrus sinensis	orange1.1g042288m.g	III	GPAT8	68.5	ER
Citrus sinensis	orange1.1g042170m.g	III	GPAT2	70.5	Mitochondria
Citrus sinensis	orange1.1g043920m.g	III	GPAT8	68.1	ER
Citrus sinensis	orange1.1g010860m.g	III	GPAT8	83.8	ER
Citrus sinensis	orange1.1g018906m.g	III	GPAT6	86.5	ER
Citrus sinensis	orange1.1g038704m.g	III	GPAT5	91.6	ER
Citrus sinensis	orange1.1g017205m	I	GPAT9	93.6	ER

Species	Protein Accession Number	Clade	The more similar Arabidopsis gene	Similarity (%)	Cellular localization
Citrus clementina	Ciclev10028672m.g	I	GPAT9	93.6	ER
Citrus clementina	Ciclev10031163m.g	III	GPAT1	77.2	Mitochondria
Citrus clementina	Ciclev10001145m.g	III	GPAT2	74.3	Mitochondria
Citrus clementina	Ciclev10013880m.g	III	GPAT8	68.5	ER
Citrus clementina	Ciclev10023483m.g	III	GPAT2	70.5	Mitochondria
Citrus clementina	Ciclev10033948m.g	III	GPAT8	52.5	ER
Citrus clementina	Ciclev10011536m.g	III	GPAT6	85.7	ER
Citrus clementina	Ciclev10031344m.g	III	GPAT7	74.1	ER
Citrus clementina	Ciclev10019825m.g	III	GPAT8	84.0	ER
Citrus clementia	Ciclev10001047m.g	II	Soluble GPAT	74.0	Chloroplast
Gossypium raimondii	Gorai.012G158300	I	GPAT9	88.1	ER
Gossypium raimondii	Gorai.005G258500	I	GPAT9	90.5	ER
Gossypium raimondii	Gorai.008G098000	III	GPAT1	78.4	Mitochondria
Gossypium raimondii	Gorai.006G181200	III	GPAT6	87.7	ER
Gossypium raimondii	Gorai.010G045500	III	GPAT2	69.4	Mitochondria
Gossypium raimondii	Gorai.007G078200	III	GPAT6	67.3	ER
Gossypium raimondii	Gorai.004G145100	III	GPAT2	65.7	Mitochondria
Gossypium raimondii	Gorai.012G112800	III	GPAT6	81.0	ER
Gossypium raimondii	Gorai.011G151300	III	GPAT4	68.7	ER
Gossypium raimondii	Gorai.004G235900	III	GPAT2	69.3	Mitochondria
Gossypium raimondii	Gorai.011G267100	III	GPAT5	78.5	ER
Gossypium raimondii	Gorai.012G071300	III	GPAT5	80.1	ER
Gossypium raimondii	Gorai.007G100100	III	GPAT4	87.6	ER
Gossypium raimondii	Gorai.011G267200	III	GPAT5	74.1	ER
Gossypium raimondii	Gorai.007G033500	II	Soluble GPAT	75.7	Chloroplast
Gossypium raimondii	Gorai.004G182200	II	Soluble GPAT	74.7	Chloroplast
Gossypium raimondii	Gorai.003G185500	II	Soluble GPAT	74.2	Chloroplast
Theobroma cacao	Thecc1EG006479	I	GPAT9	82.7	ER
Theobroma cacao	Thecc1EG001873	III	GPAT1	78.9	Mitochondria
Theobroma cacao	Thecc1EG034986	III	GPAT2	61.1	Mitochondria
Theobroma cacao	Thecc1EG034985	III	GPAT2	64.0	Mitochondria
Theobroma cacao	Thecc1EG004855	III	GPAT2	73.2	Mitochondria
Theobroma cacao	Thecc1EG021737	III	GPAT6	86.5	ER
Theobroma cacao	Thecc1EG018071	III	GPAT6	68.8	ER
Theobroma cacao	Thecc1EG026783	III	GPAT2	70.8	Mitochondria
Theobroma cacao	Thecc1EG010070	III	GPAT4	59.9	ER
Theobroma cacao	Thecc1EG042716	III	GPAT5	76.4	ER
Theobroma cacao	Thecc1EG005317	III	GPAT4	88.6	ER
Theobroma cacao	Thecc1EG016600	II	Soluble GPAT	72.2	Chloroplast
Arabidopsis lyrata	919369	III	GPAT1	98.1	Mitochondria
Arabidopsis lyrata	470198	III	GPAT2	96.8	Mitochondria
Arabidopsis lyrata	943550	III	GPAT3	98.8	Mitochondria
Arabidopsis lyrata	478480	III	GPAT5	98.8	ER
Arabidopsis lyrata	482818	III	GPAT6	99.4	ER
Arabidopsis lyrata	918798	III	GPAT4	99.6	ER
Arabidopsis lyrata	490527	III	GPAT8	98.4	ER
Arabidopsis lyrata	349873	III	GPAT4	63.5	ER
Arabidopsis lyrata	950810	I	GPAT9	99.5	ER
Arabidopsis lyrata	AL1G46330	II	Soluble GPAT	99.6	Chloroplast
Brassica rapa	Brara.A00307	I	GPAT9	97.6	ER
Brassica rapa	Brara.J01416	I	GPAT9	97.0	ER
Brassica rapa	Brara.J00449	III	GPAT1	89.1	Mitochondria
Brassica rapa	Brara.K01852	III	GPAT8	90.4	ER
Brassica rapa	Brara.A03408	III	GPAT5	85.7	ER
Brassica rapa	Brara.I00118	III	GPAT3	83.2	Mitochondria
Brassica rapa	Brara.C02835	III	GPAT3	88.7	Mitochondria

Species	Protein Accession Number	Clade	The more similar Arabidopsis gene	Similarity (%)	Cellular localization
Brassica rapa	Brara.I05638	III	GPAT4	93.8	ER
Brassica rapa	Brara.E02936	III	GPAT5	87.2	ER
Brassica rapa	Brara.D02328	III	GPAT6	94.2	ER
Brassica rapa	Brara.J00035	III	GPAT4	96.2	ER
Brassica rapa	Brara.E00709	III	GPAT6	94.0	ER
Brassica rapa	Brara.J02607	III	GPAT7	90.0	ER
Brassica rapa	Brara.E02888	III	GPAT5	78.4	ER
Brassica rapa	Brara.H00582	II	Soluble GPAT	92.7	Chloroplast
Brassica rapa	Brara.E01731	II	Soluble GPAT	88.0	Chloroplast
Brassica rapa	Brara.I02607	II	Soluble GPAT	86.2	Chloroplast
Capsella grandiflora	Cagra.1671s0280	III	GPAT1	96.2	Mitochondria
Capsella grandiflora	Cagra.1968s0023	III	GPAT2	92.8	Mitochondria
Capsella grandiflora	Cagra.0334s0034	III	GPAT3	93.1	Mitochondria
Capsella grandiflora	Cagra.1655s0027	III	GPAT5	92.6	ER
Capsella grandiflora	Cagra.1968s0101	III	GPAT4	95.8	ER
Capsella grandiflora	Cagra.2117s0053	III	GPAT7	96.8	ER
Capsella grandiflora	Cagra.1261s0029	III	GPAT8	93.8	ER
Capsella grandiflora	Cagra.1655s0038	III	GPAT5	83.6	ER
Capsella grandiflora	Cagra.15555s0001	III	GPAT6	93.1	ER
Capsella grandiflora	Cagra.2519s0033	I	GPAT9	98.4	ER
Capsella grandiflora	Cagra.3957s0001	II	Soluble GPAT	97.4	Chloroplast
Capsella rubella	Carubv10026601m.g	I	GPAT9	98.1	ER
Capsella rubella	Carubv10012249m	III	GPAT1	96.0	Mitochondria
Capsella rubella	Carubv10008716m	III	GPAT2	86.6	Mitochondria
Capsella rubella	Carubv10000657m	III	GPAT3	89.8	Mitochondria
Capsella rubella	Carubv10013500m	III	GPAT5	94.6	ER
Capsella rubella	Carubv10023060m	III	GPAT6	94.6	ER
Capsella rubella	Carubv10008926m	III	GPAT4	96.0	ER
Capsella rubella	Carubv10000788m	III	GPAT7	96.4	ER
Capsella rubella	Carubv10000780m	III	GPAT8	93.8	ER
Capsella rubella	Carubv10015640m	III	GPAT5	84.2	ER
Capsella rubella	Carubv10012611m.g	II	Soluble GPAT	98.0	Chloroplast
Eutrema salsugineum	Thhalv10007168m	III	GPAT1	94.0	Mitochondria
Eutrema salsugineum	Thhalv10020574m	III	GPAT5	91.6	ER
Eutrema salsugineum	Thhalv10007347m	III	GPAT2	91.1	Mitochondria
Eutrema salsugineum	Thhalv10028577m	III	GPAT3	87.5	Mitochondria
Eutrema salsugineum	Thhalv10007431m	III	GPAT4	93.8	ER
Eutrema salsugineum	Thhalv10016551m	III	GPAT6	94.8	ER
Eutrema salsugineum	Thhalv10028601m	III	GPAT4	90.8	ER
Eutrema salsugineum	Thhalv10015521m	III	GPAT7	93.2	ER
Eutrema salsugineum	Thhalv10013846m.g	I	GPAT9	97.9	ER
Eutrema salsugineum	Thhalv10007540m.g	II	Soluble GPAT	91.3	Chloroplast
Cucumis sativus	Cucsa.099550	I	GPAT9	90.7	ER
Cucumis sativus	Cucsa.185470	III	GPAT1	72.6	Mitochondria
Cucumis sativus	Cucsa.339200	III	GPAT6	67.7	ER
Cucumis sativus	Cucsa.176200	III	GPAT6	85.1	ER
Cucumis sativus	Cucsa.165180	III	GPAT5	77.2	ER
Cucumis sativus	Cucsa.135570	III	GPAT4	84.8	ER
Cucumis sativus	Cucsa.204890	III	GPAT2	64.9	Mitochondria
Cucumis sativus	Cucsa.312830	II	Soluble GPAT	72.6	Chloroplast
Glycine max	Glyma.14G028300	III	GPAT1	78.0	Mitochondria
Glycine max	Glyma.02G286500	III	GPAT1	75.5	Mitochondria
Glycine max	Glyma.18G107100	III	GPAT1	73.8	Mitochondria
Glycine max	Glyma.08G309200	III	GPAT1	72.2	Mitochondria
Glycine max	Glyma.14G167300	III	GPAT2	66.3	Mitochondria
Glycine max	Glyma.13G085700	III	GPAT2	66.8	Mitochondria

Species	Protein Accession Number	Clade	The more similar Arabidopsis gene	Similarity (%)	Cellular localization
Glycine max	Glyma.03G221100	III	GPAT2	70.3	Mitochondria
Glycine max	Glyma.19G218100	III	GPAT2	69.8	Mitochondria
Glycine max	Glyma.02G010600	III	GPAT2	67.2	Mitochondria
Glycine max	Glyma.07G146800	III	GPAT6	88.1	ER
Glycine max	Glyma.14G167400	III	GPAT2	68.6	Mitochondria
Glycine max	Glyma.03G221300	III	GPAT2	74.7	Mitochondria
Glycine max	Glyma.10G011000.	III	GPAT2	67.6	Mitochondria
Glycine max	Glyma.18G197800	III	GPAT6	81.1	ER
Glycine max	Glyma.01G113200	III	GPAT6	88.2	ER
Glycine max	Glyma.03G078600	III	GPAT6	84.7	ER
Glycine max	Glyma.20G070400	III	GPAT6	71.1	ER
Glycine max	Glyma.10G119900	III	GPAT6	71.7	ER
Glycine max	Glyma.02G249300	III	GPAT5	81.1	ER
Glycine max	Glyma.03G008300	III	GPAT8	86.2	ER
Glycine max	Glyma.07G069700	III	GPAT8	87.4	ER
Glycine max	Glyma.14G067200	III	GPAT5	78.4	ER
Glycine max	Glyma.06G255600	III	GPAT6	79.5	ER
Glycine max	Glyma.09G119200	I	GPAT9	92.3	ER
Glycine max	Glyma.08G085800	I	GPAT9	92.0	ER
Glycine max	Glyma.05G131100	I	GPAT9	91.7	ER
Glycine max	Glyma.01G014200	II	Soluble GPAT	69.0	Chloroplast
Glycine max	Glyma.09G207900	II	Soluble GPAT	65.7	Chloroplast
Medicago truncatula	Medtr4g127910	I	GPAT9	91.0	ER
Medicago truncatula	Medtr2g438210	I	GPAT9	88.7	ER
Medicago truncatula	Medtr5g091660	III	GPAT1	66.6	Mitochondria
Medicago truncatula	Medtr3g448430	III	GPAT1	66.3	Mitochondria
Medicago truncatula	Medtr5g061520	III	GPAT2	67.5	Mitochondria
Medicago truncatula	Medtr4g415290	III	GPAT6	89.5	ER
Medicago truncatula	Medtr7g067380	III	GPAT6	87.6	ER
Medicago truncatula	Medtr1g040500	III	GPAT6	62.3	ER
Medicago truncatula	Medtr8g030620	III	GPAT8	87.3	ER
Medicago truncatula	Medtr1g086650	III	GPAT2	70.1	Mitochondria
Medicago truncatula	Medtr5g080360	III	GPAT5	77.1	ER
Medicago truncatula	Medtr5g029230	II	Soluble GPAT	68.7	Chloroplast
Phaseolus vulgaris	Phvul.006G033100	III	GPAT1	77.6	Mitochondria
Phaseolus vulgaris	Phvul.008G191600	III	GPAT1	79.4	Mitochondria
Phaseolus vulgaris	Phvul.008G169500	III	GPAT2	71.7	Mitochondria
Phaseolus vulgaris	Phvul.008G106700	III	GPAT6	88.9	ER
Phaseolus vulgaris	Phvul.008G169400	III	GPAT2	68.3	Mitochondria
Phaseolus vulgaris	Phvul.007G233600	III	GPAT6	71.9	ER
Phaseolus vulgaris	Phvul.007G212600	III	GPAT2	75.7	Mitochondria
Phaseolus vulgaris	Phvul.010G099700	III	GPAT4	89.4	ER
Phaseolus vulgaris	Phvul.L005200	III	GPAT6	87.2	ER
Phaseolus vulgaris	Phvul.003G022900	I	GPAT9	92.6	ER
Phaseolus vulgaris	Phvul.002G191600	I	GPAT9	92.3	ER
Phaseolus vulgaris	Phvul.002G136600	II	Soluble GPAT	67.0	Chloroplast
Chlamydomonas reinhardtii	Cre06.g273250	I	GPAT9	57.0	ER
Chlamydomonas reinhardtii	Cre02.g143000	II	Soluble GPAT	53.2	Chloroplast
Volvox carteri	Vocar.0002s0353	I	GPAT9	57.9	ER
Volvox carteri	Vocar.0054s0035	II	Soluble GPAT	54.9	Chloroplast
Coccomyxa subellipsoidea	4945	I	GPAT9	71.2	ER
Coccomyxa subellipsoidea	31435	II	Soluble GPAT	51.7	Chloroplast
Micromonas pusilla	36845	I	GPAT9	72.0	ER
Micromonas pusilla	158788	II	Soluble GPAT	39.6	Chloroplast
Micromonas sp.	94289	I	GPAT9	64.3	ER
Micromonas sp.	106290	II	Soluble GPAT	46.7	Chloroplast

**Species**	**Protein Accession Number**	**Clade**	**The more similar Arabidopsis gene**	**Similarity (%)**	**Cellular localization**
Ostreococcus lucimarinus	51690	I	GPAT9	63.7	ER
Otreococcus lucimarinus	30035	II	Soluble GPAT	48.5	Chloroplast
Otreococcus lucimarinus	119500	II	Soluble GPAT	48.5	Chloroplast

ER: Endoplasmic reticulum
