# Supplementary material for: Genome-wide analysis of the Glycerol-3-Phosphate Acyltransferase (GPAT) gene family reveals the evolution and diversification of plant GPATs
Source: Genet Mol Biol. 2018 Mar 19;41(1 Suppl 1):355–70. doi: 10.1590/1678-4685-GMB-2017-0076 (PMC5913721; doi:10.1590/1678-4685-GMB-2017-0076)
Supplement: Supplementary file 3 [file 1415-4757-GMB-41-01-2017-0076-s003.pdf]

Supplementary Material to “Genome-wide analysis of the Glycerol-3-Phosphate Acyltransferase (GPAT) gene family reveals the evolution and diversification of plant GPATs“

Table S3 - Predictions of transmembrane domains of plant GPAT proteins. Predicted transmembrane structures of representative species were obtained using the transmembrane prediction server TMHMM-2.0 and the PROTTER tool with the complete protein sequences.

Species	Gene	TMHMM	Protter
Arabidopsis thaliana	GPAT1	2	3
	GPAT2	1	4
	GPAT3	2	4
	GPAT4	3	3
	GPAT5	2	2
	GPAT6	3	3
	GPAT7	2	2
	GPAT8	2	3
	GPAT9	3	3
	Soluble GPAT	0	0
Oryza sativa	LOC_Os01g44069	2	3
	LOC_Os10g27330	1	3
	LOC_Os03g52570	1	2
	LOC_Os01g63580	3	3
	LOC_Os05g38350	1	2
	LOC_Os11g45400	1	2
	LOC_Os02g02340	3	3
	LOC_Os05g20100	1	3
	LOC_Os08g03700	3	4
	LOC_Os01g19390	1	3
	LOC_Os12g37600	0	3
	LOC_Os03g61720	3	3
	LOC_Os01g14900	0	1
	LOC_Os05g37600	0	1
	LOC_Os10g41070	1	2
	LOC_Os01g22560	1	1
	LOC_Os07g34730	2	3
	LOC_Os10g42720	0	0
Selaginella moellendorffii	80075	0	2
	118155	2	3
	90219	2	4
	405228	2	4

Species	Gene	TMHMM	Protter
	80614	2	2
	170163	2	3
	164779	3	4
	63752	2	3
	233008	3	4
	405007	0	3
	152980	3	3
	132845	0	0
Physcomitrella patens	Pp3c6_29200	4	4
	Pp3c2_18040	2	3
	Pp3c7_7840	3	3
	Pp3c5_1510	4	3
	Pp3c20_9340	1	4
	Pp3c6_29290	3	3
	Pp3c8_21680	2	2
	Pp3c11_26030	3	3
	Pp3c14_5980	0	0
	Pp3c7_2970	0	0
Volvox carteri	Vocar_0002s0353	2	4
	Vocar_0054s0035	0	0
