# Supplementary material for: Genome-wide analysis of the Glycerol-3-Phosphate Acyltransferase (GPAT) gene family reveals the evolution and diversification of plant GPATs
Source: Genet Mol Biol. 2018 Mar 19;41(1 Suppl 1):355–70. doi: 10.1590/1678-4685-GMB-2017-0076 (PMC5913721; doi:10.1590/1678-4685-GMB-2017-0076)
Supplement: Supplementary file 4 [file 1415-4757-GMB-41-01-2017-0076-s004.pdf]

Supplementary Material to "Genome-wide analysis of the Glycerol-3-Phosphate Acyltransferase (GPAT) gene family reveals the evolution and diversification of plant GPATs"

GPAT1_Ath_AT1G06520

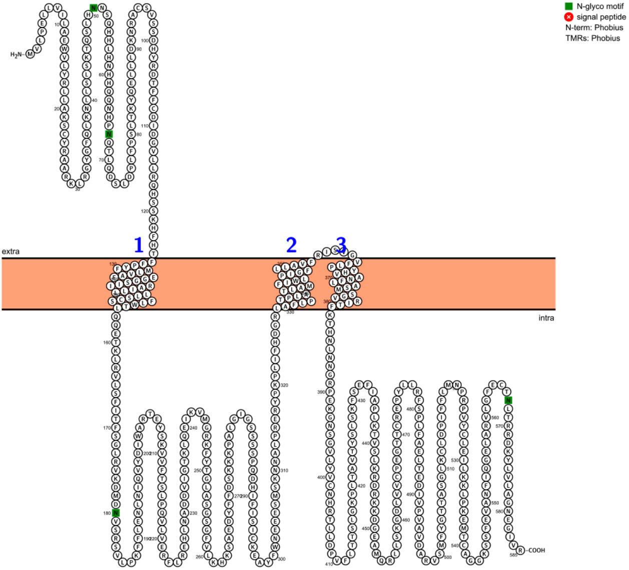

GPAT2_Ath_AT1G02390

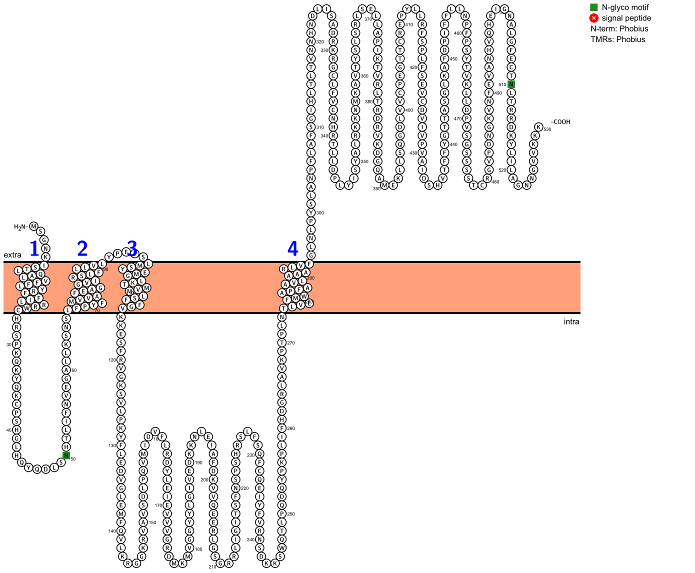

GPAT3_Ath_AT4G01950

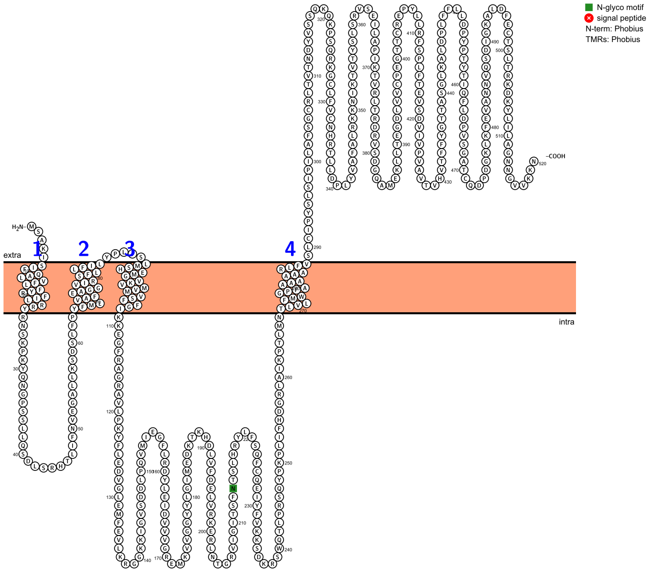

GPAT4_Ath_AT1G01610

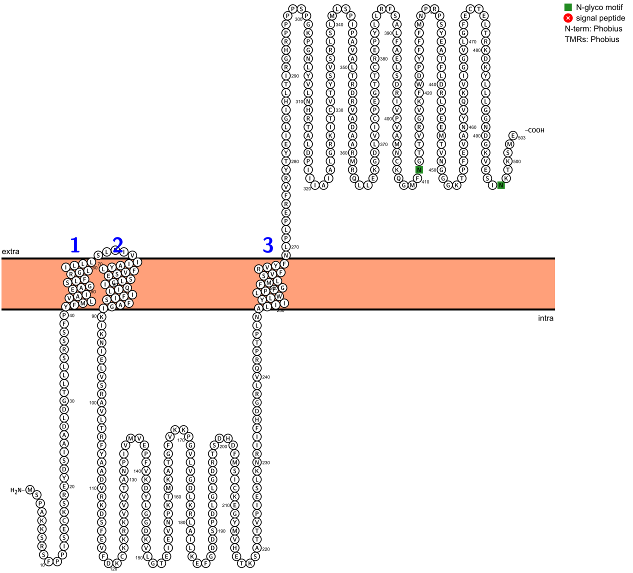

GPAT5_Ath_AT3G11430

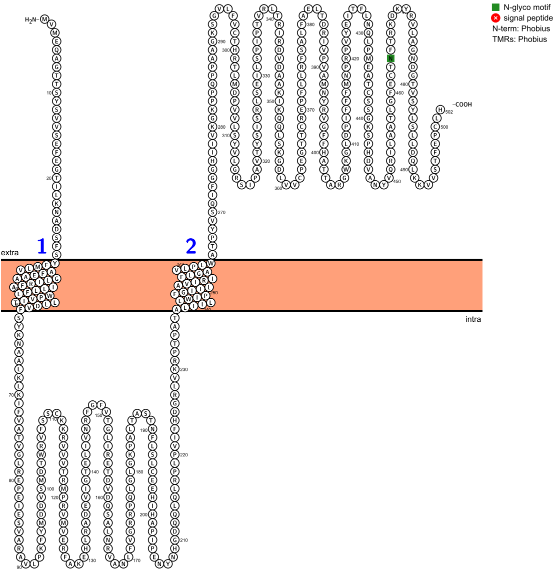

GPAt6_Ath_AT2G38110

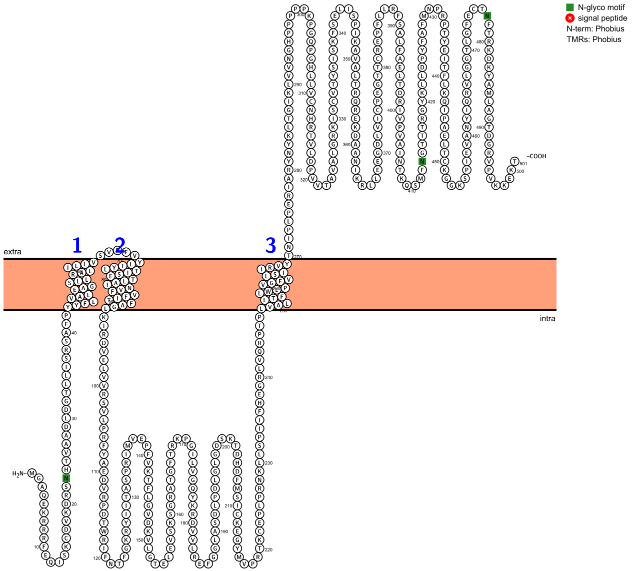

GPAT7_Ath_AT5G06090

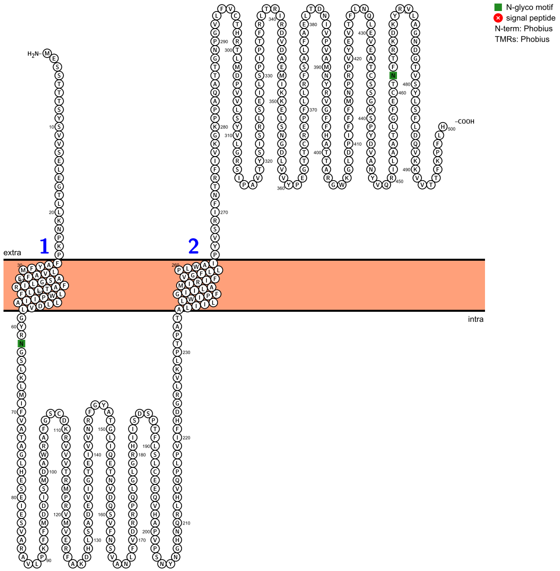

GPAT8_Ath_AT4G00400

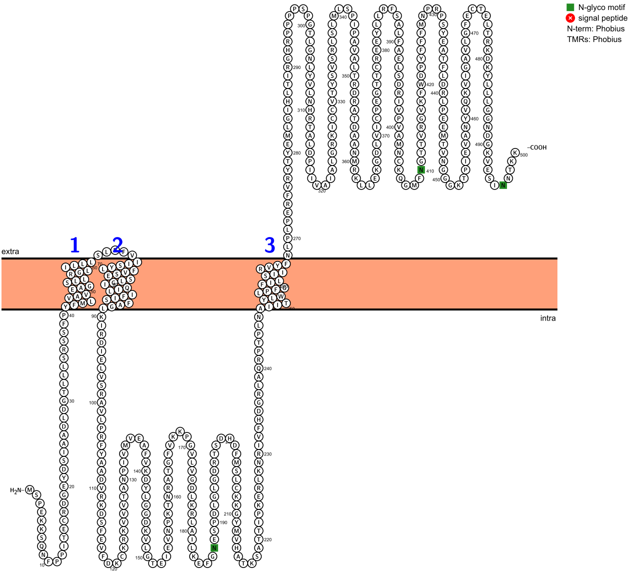

GPAT9_Ath_AT5G60620

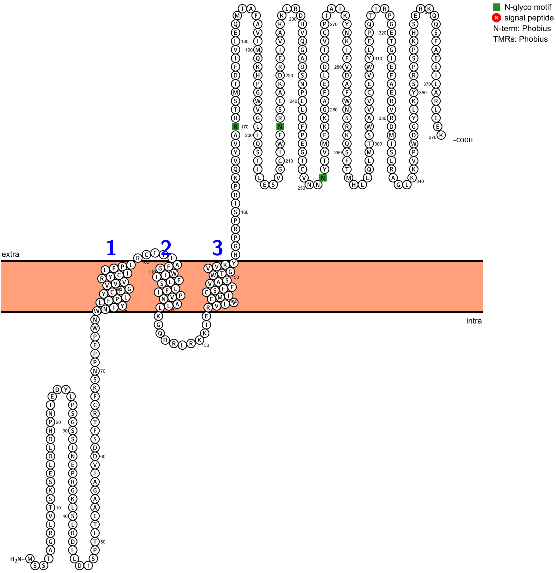

Ath_AT3G11325

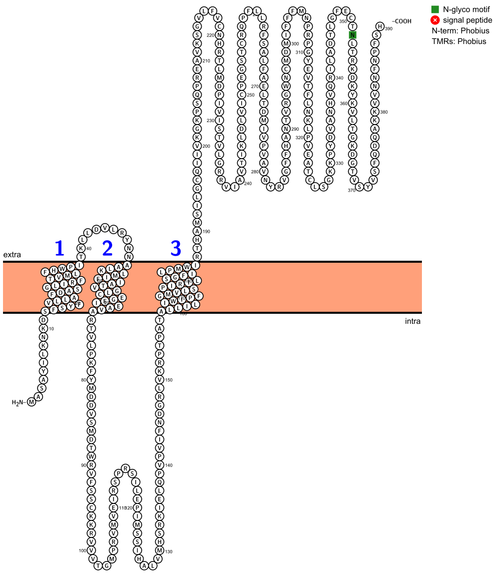

Ath_AT1G32200

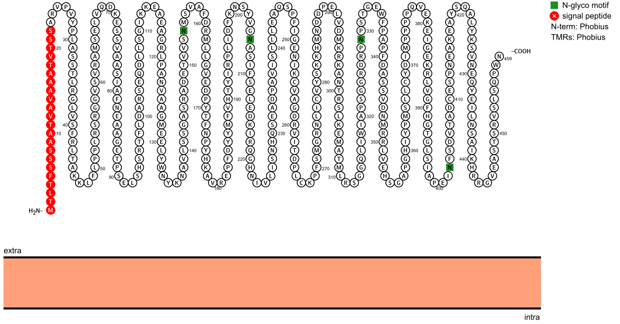

Osa_LOC_Os01g44069

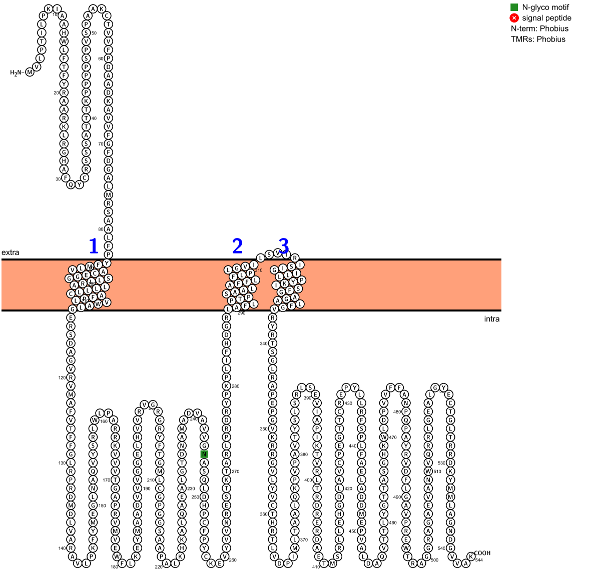

Osa_LOC_Os10g27330

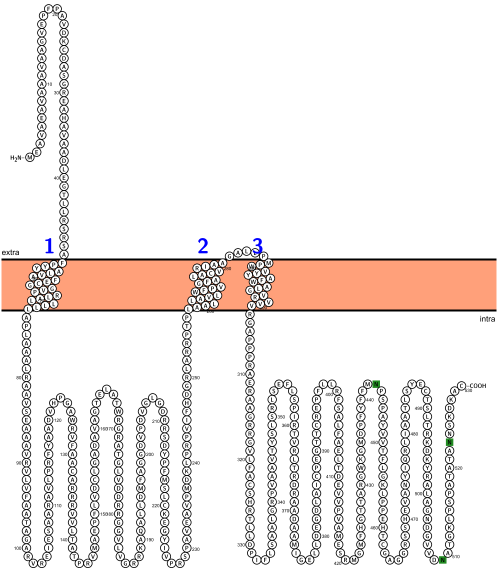

Osa_LOC_Os03g52570

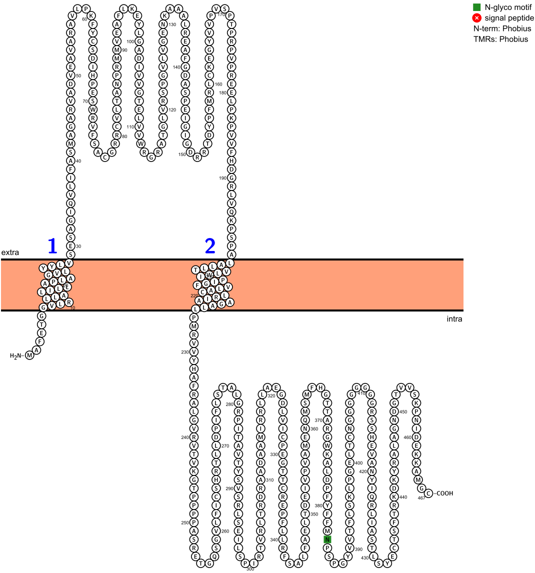

Osa_LOC_Os01g63580

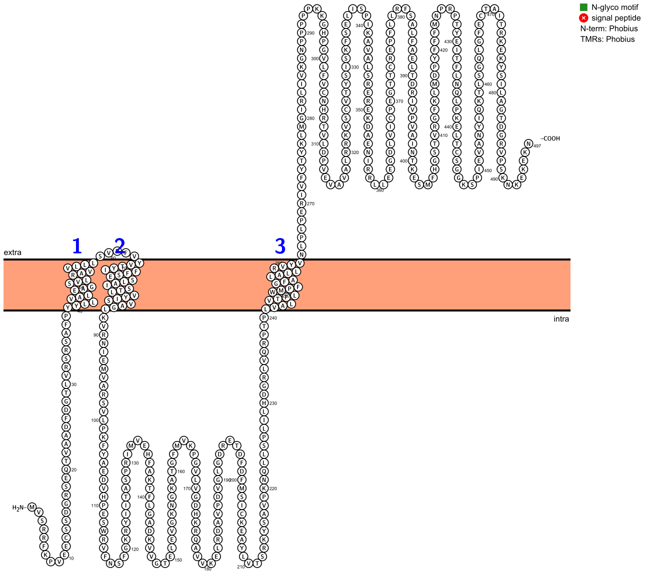

Osa_LOC_Os05g38350

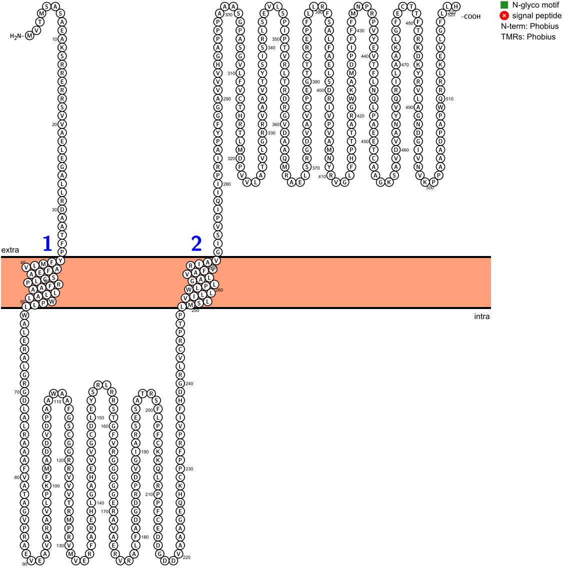

Osa_LOC_Os11g45400

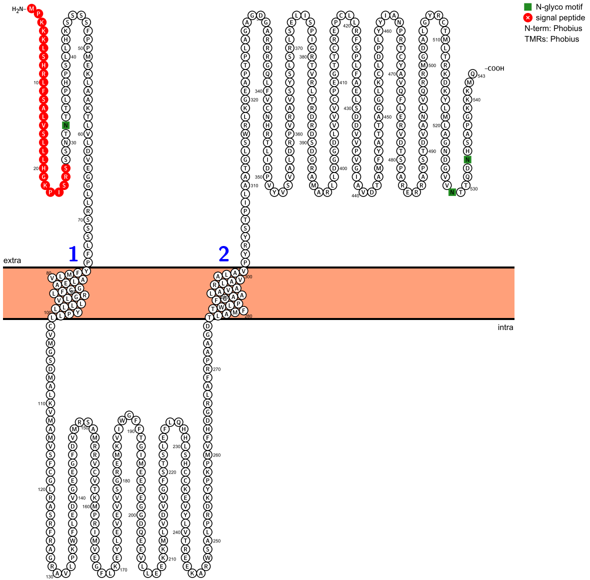

Osa_LOC_Os02g02340

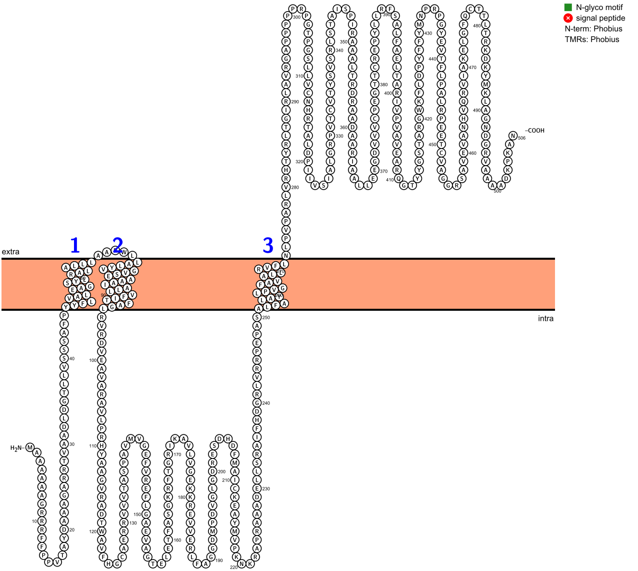

Osa_LOC_Os05g20100

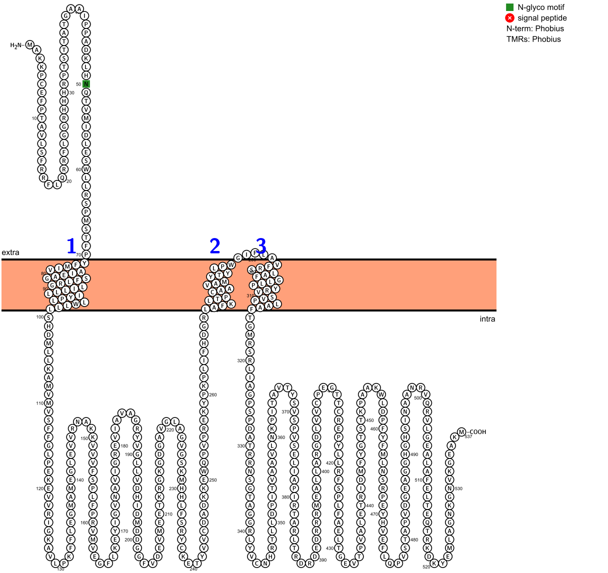

Osa_LOC_Os08g03700

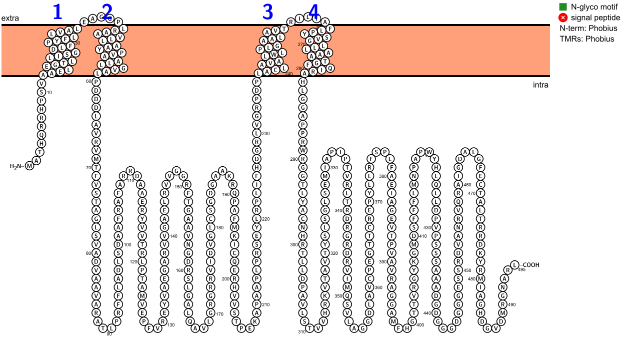

Osa_LOC_Os01g19390

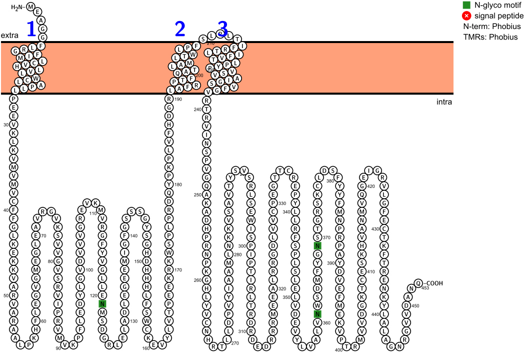

Osa_LOC_Os12g37600

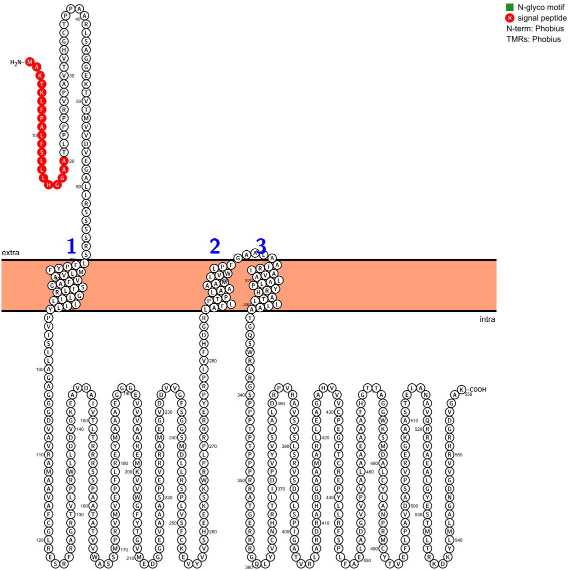

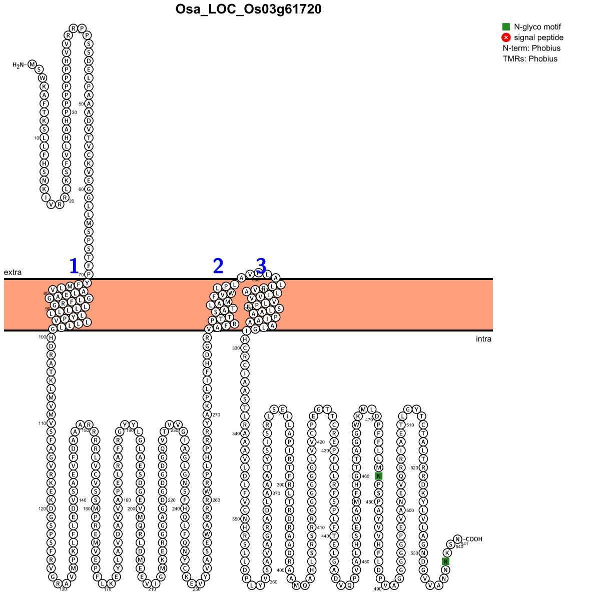

Osa_LOC_Os03g61720
N-glyco motif
signal peptide
N-term: Phobius
TMRs: Phobius
extra
intra
1
2
3

Osa_LOC_Os01g14900

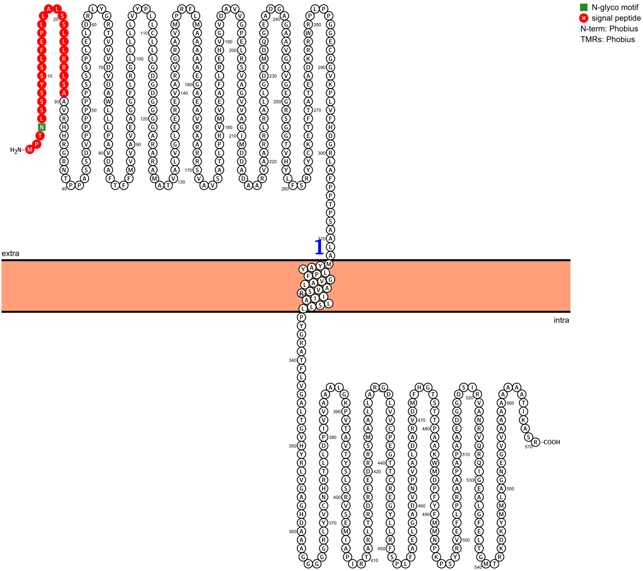

Osa_LOC_Os05g37600

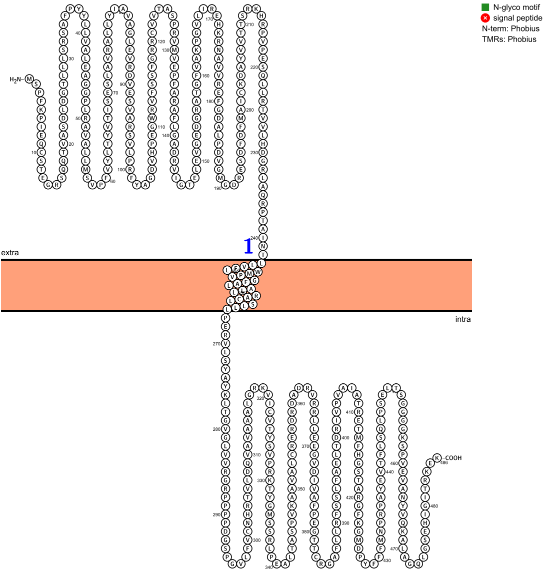

Osa_LOC_Os10g41070

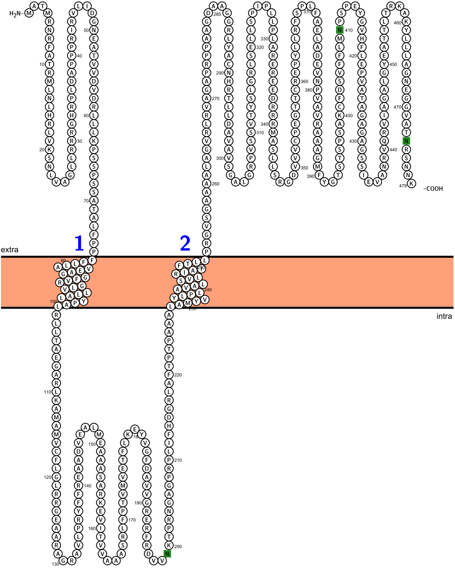

Osa_LOC_Os01g22560

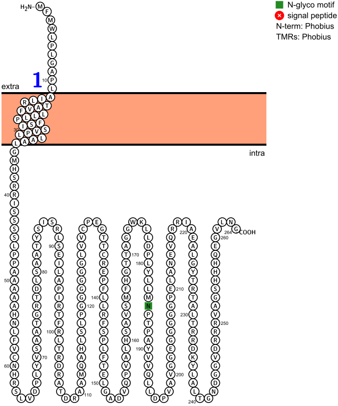

Osa_LOC_Os07g34730

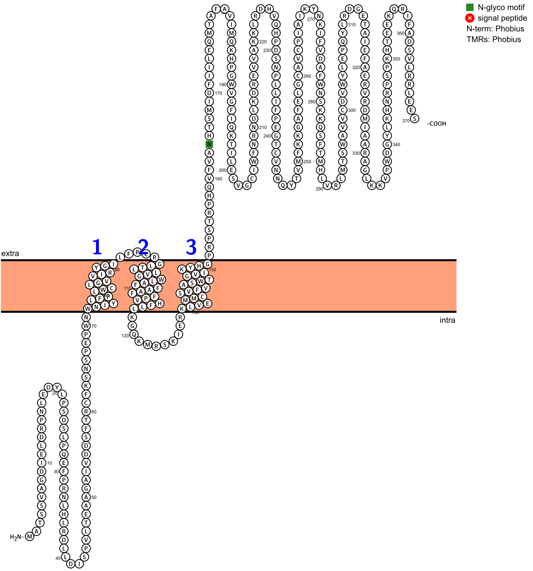

Osa_LOC_Os10g42720

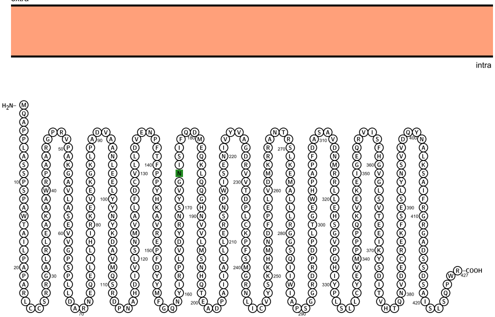

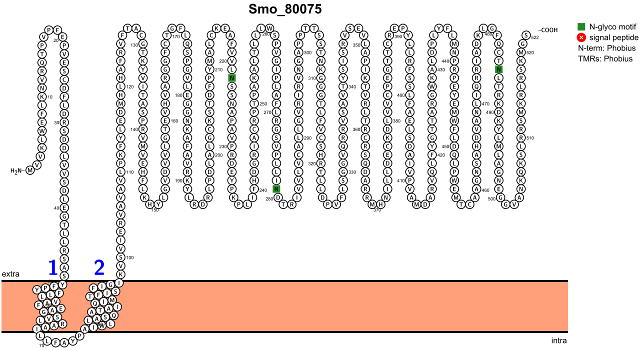
Smo_80075
N-glyco motif
signal peptide
N-term: Phobius
TMRs: Phobius
extra
intra
1
2
H2N-
-COOH

Smo_118155

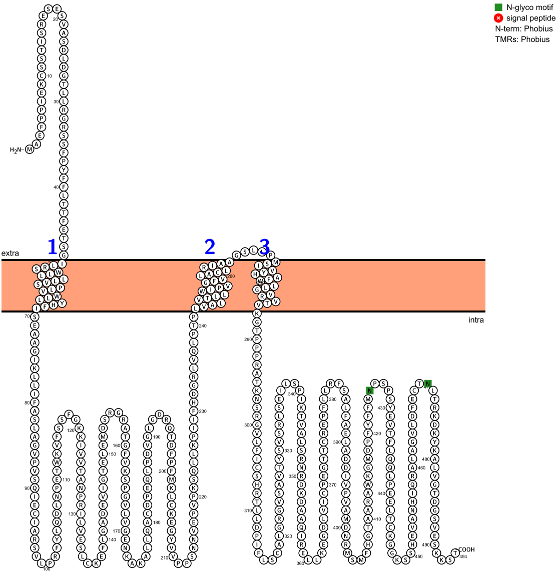
N-glyco motif
signal peptide
N-term: Phobius
TMRs: Phobius
extra
intra
1
2
3
H2N
COOH

Smo_90219

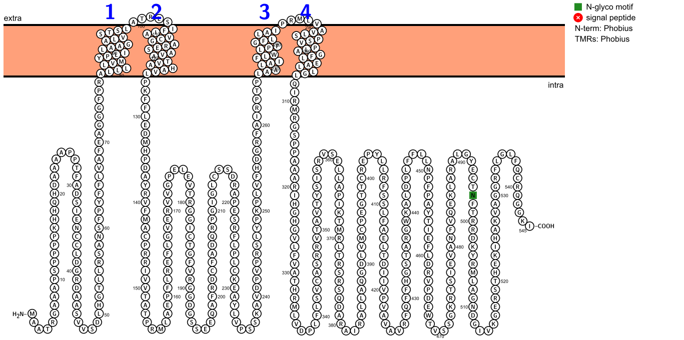

1
2
3
4
extra
intra
N-glyco motif
signal peptide
N-term: Phobius
TMRs: Phobius
H2N-
-COOH

Smo_405228

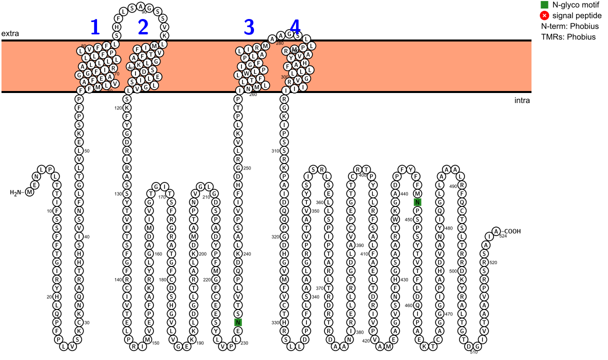
N-glyco motif
signal peptide
N-term: Phobius
TMRs: Phobius
extra
intra
1
2
3
4
H2N-
-COOH

Smo_80614

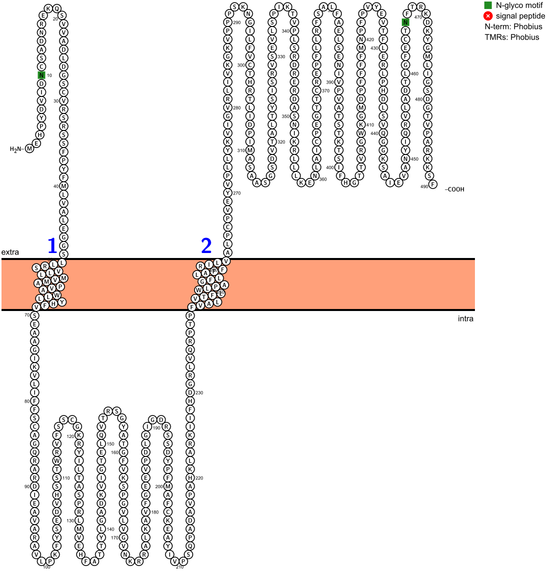

N-glyco motif
signal peptide
N-term: Phobius
TMRs: Phobius
extra
intra
1
2
H2N
COOH

Smo_170163

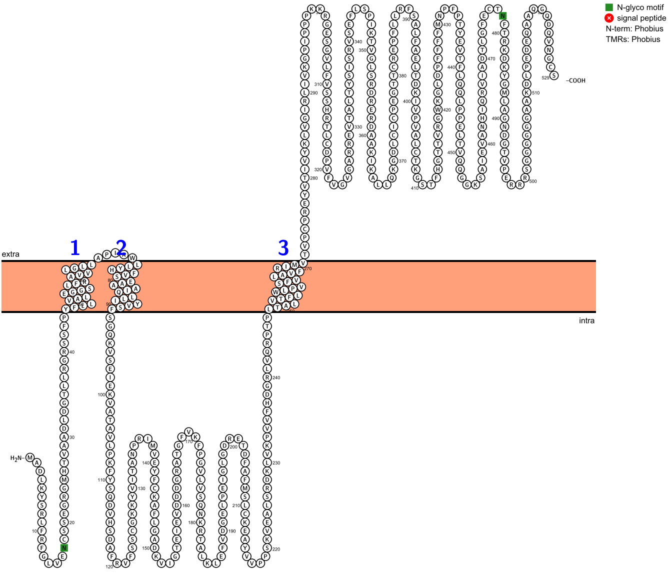
N-glyco motif
signal peptide
N-term: Phobius
TMRs: Phobius
extra
intra
1
2
3
H2N-
-COOH

Smo_164779

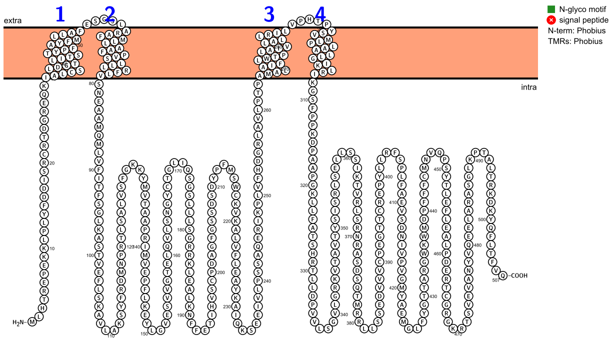

N-glyco motif
signal peptide
N-term: Phobius
TMRs: Phobius
extra
intra
1
2
3
4
H2N
COOH

Smo_63752

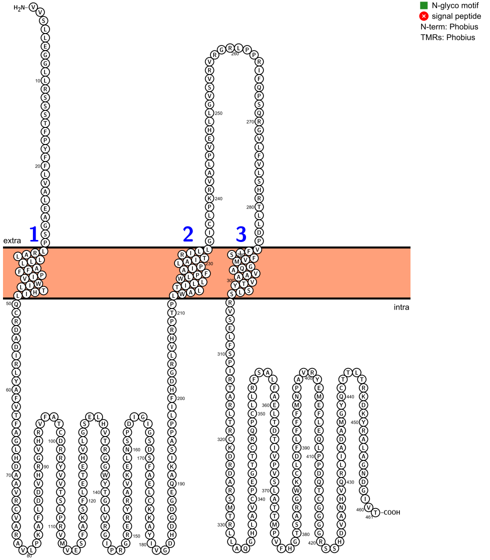
N-glyco motif
signal peptide
N-term: Phobius
TMRs: Phobius
extra
intra
1
2
3
H2N-
-COOH

Smo_233008

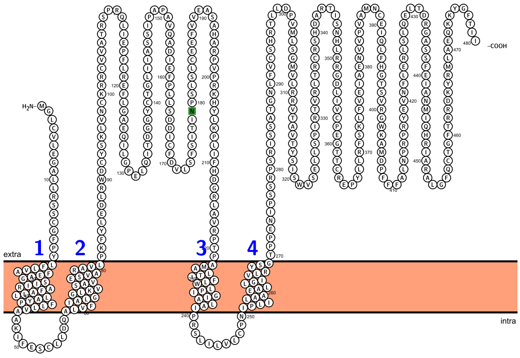

N-glyco motif
signal peptide
N-term: Phobius
TMRs: Phobius
H2N–
–COOH
extra
intra
1
2
3
4

Smo_405007

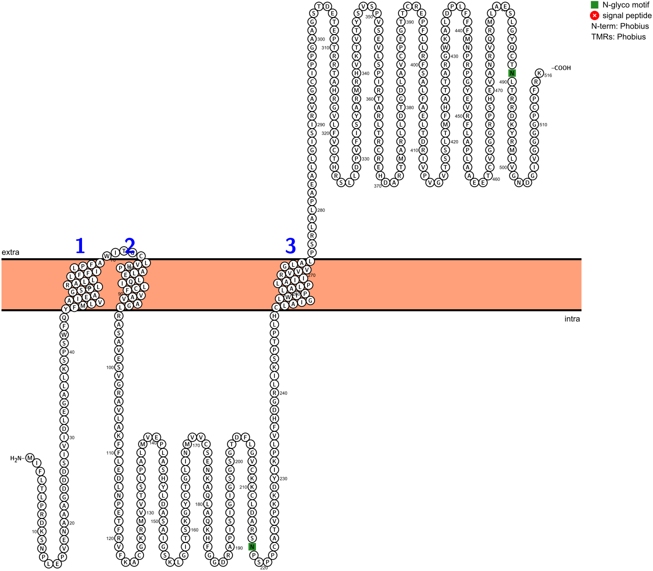

N-glyco motif
signal peptide
N-term: Phobius
TMRs: Phobius
extra
intra
1
2
3
H2N-
-COOH

Smo_152980

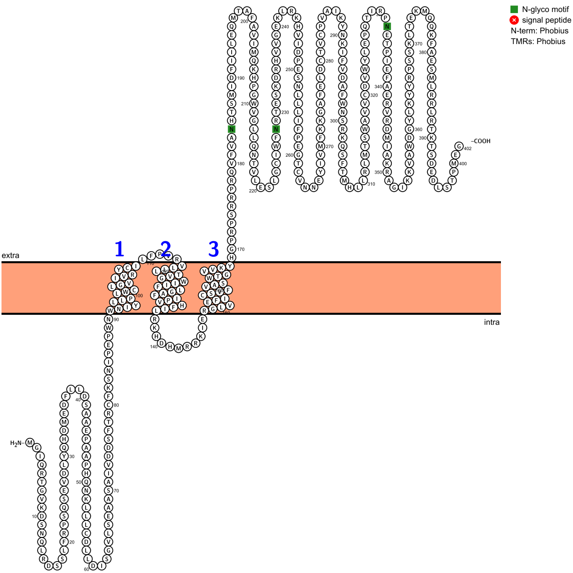
N-glyco motif
signal peptide
N-term: Phobius
TMRs: Phobius
extra
intra
1
2
3
H2N-
-COOH

Smo_132845

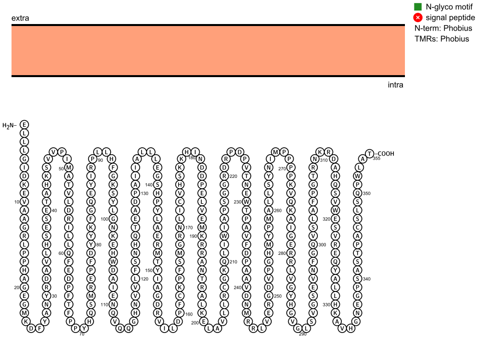

N-glyco motif
signal peptide
N-term: Phobius
TMRs: Phobius
extra
intra
H2N–
–COOH

Ppa_Pp3c6_29200

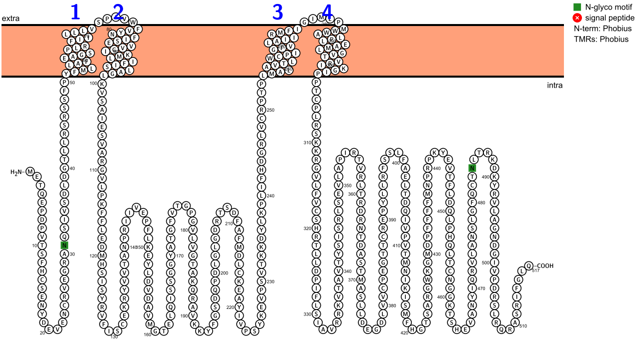

Ppa_Pp3c2_18040

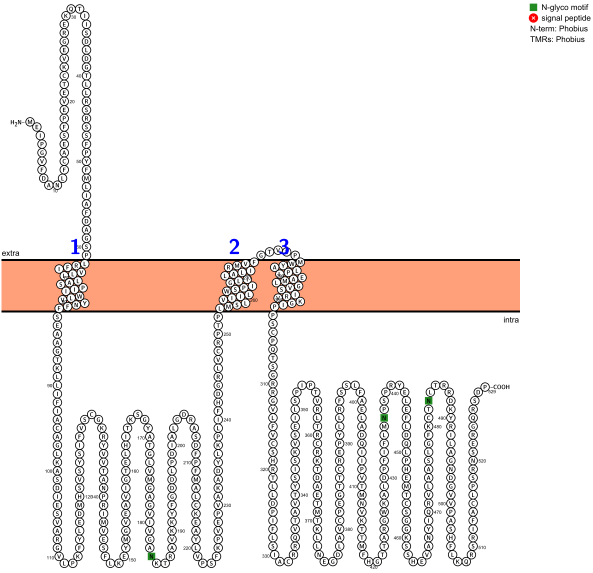

Ppa_Pp3c7_7840

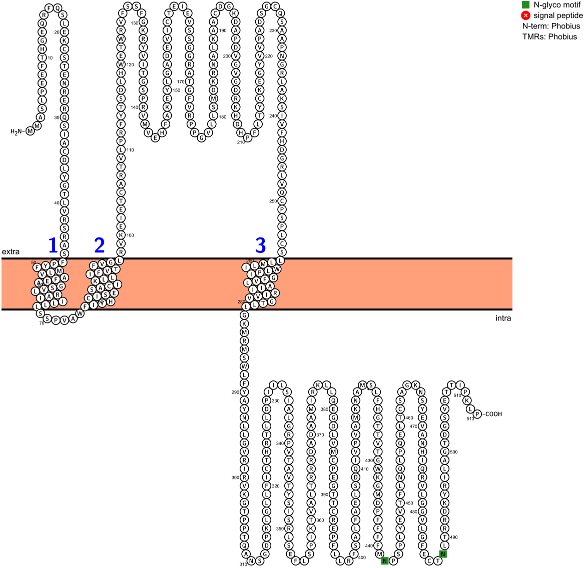

Ppa_Pp3c5_1510

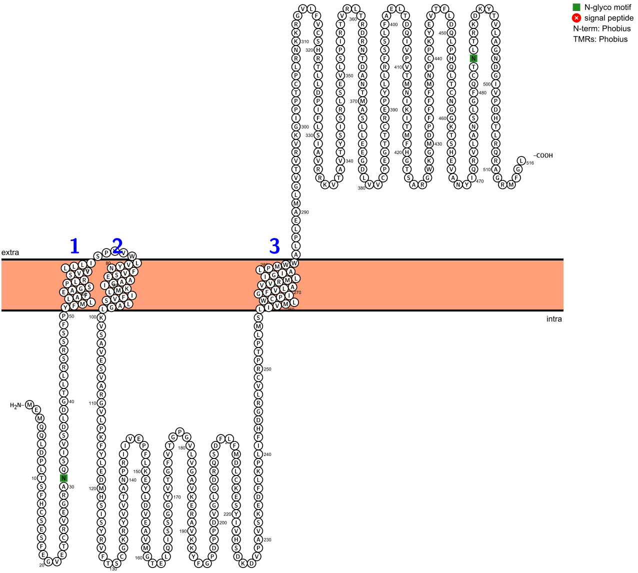

Ppa_Pp3c20_9340

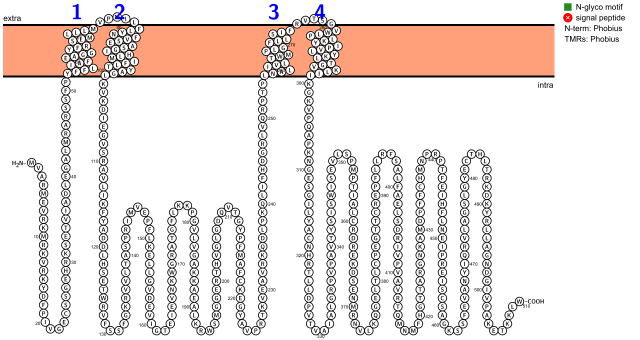

Ppa_Pp3c6_29290

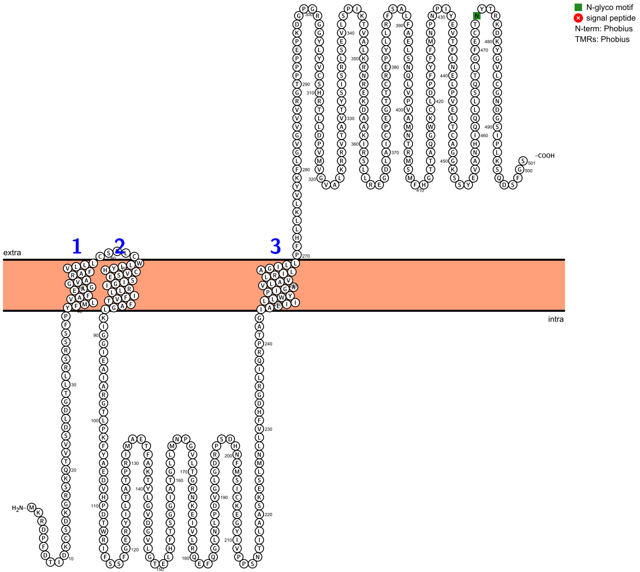

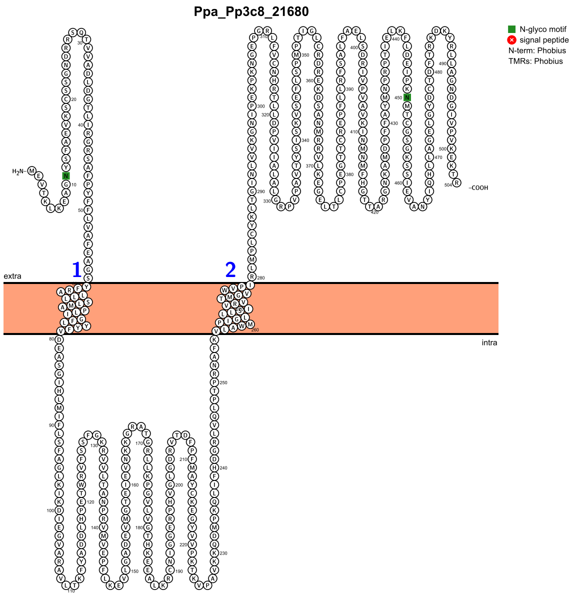

Ppa_Pp3c8_21680
N-glyco motif
signal peptide
N-term: Phobius
TMRs: Phobius
extra
intra
1
2
H_2N-
-COOH

Ppa_Pp3c11_26030

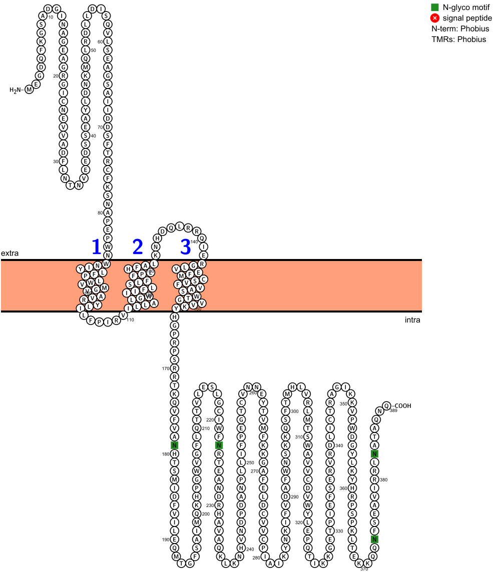

Ppa_Pp3c14_5980

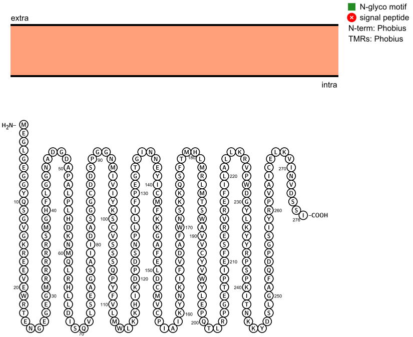

Ppa_Pp3c7_2970

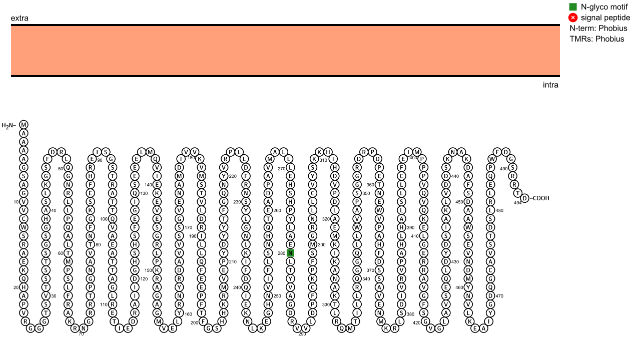

Vca_Vocar_0002s0353

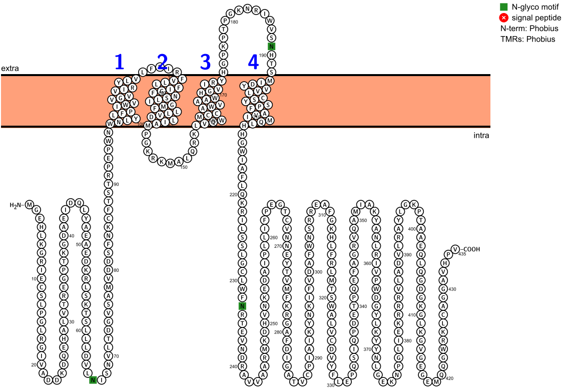

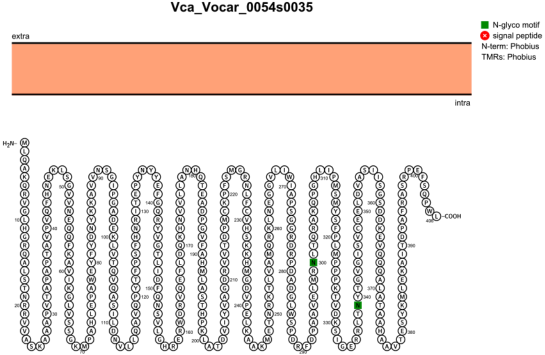

Figure S1 - Properties of GPAT protein sequences of representative species.
