# Supplementary material for: Genome-wide analysis of the Glycerol-3-Phosphate Acyltransferase (GPAT) gene family reveals the evolution and diversification of plant GPATs
Source: Genet Mol Biol. 2018 Mar 19;41(1 Suppl 1):355–70. doi: 10.1590/1678-4685-GMB-2017-0076 (PMC5913721; doi:10.1590/1678-4685-GMB-2017-0076)
Supplement: Supplementary file 5 [file 1415-4757-GMB-41-01-2017-0076-s005.pdf]

Supplementary Material to "Genome-wide analysis of the Glycerol-3-Phosphate Acyltransferase (GPAT) gene family reveals the evolution and diversification of plant GPATs"

Dataset: 105 anatomical parts from data selection: AT_AFFY_ATH1-0

Showing 9 measure(s) of 9 gene(s) on selection: AT-0

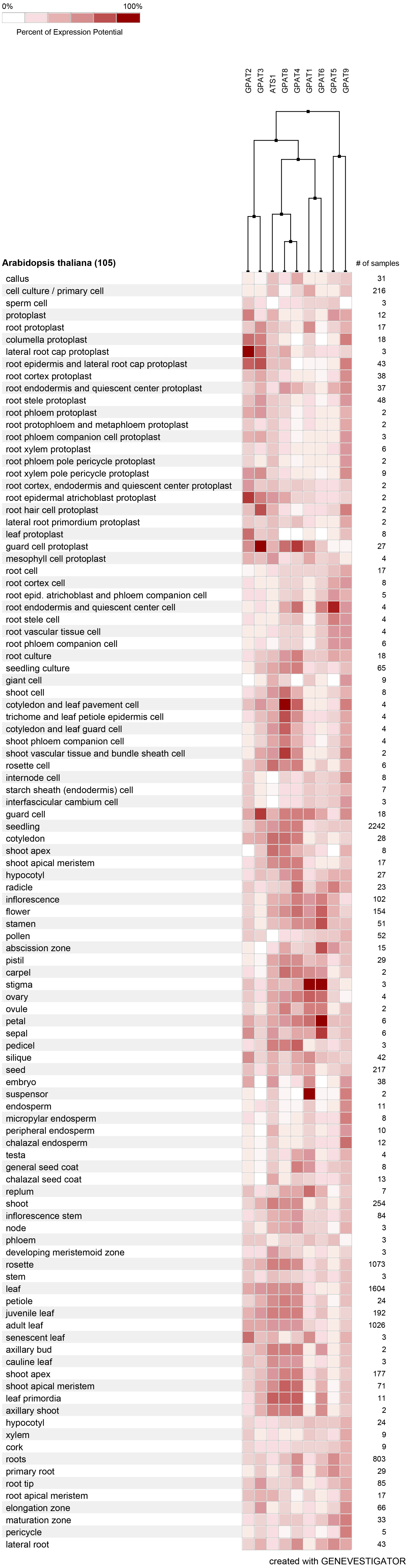

Figure S2 - Microarray data analysis from Genevestigator showing expression pattern of GPATs in anatomical parts of *Arabidopsis thaliana*.
