# Supplementary material for: Genome-wide analysis of the Glycerol-3-Phosphate Acyltransferase (GPAT) gene family reveals the evolution and diversification of plant GPATs
Source: Genet Mol Biol. 2018 Mar 19;41(1 Suppl 1):355–70. doi: 10.1590/1678-4685-GMB-2017-0076 (PMC5913721; doi:10.1590/1678-4685-GMB-2017-0076)
Supplement: Supplementary file 10 [file 1415-4757-GMB-41-01-2017-0076-s011.pdf]

# Supplementary Material to "Genome-wide analysis of the Glycerol-3-Phosphate Acyltransferase (GPAT) gene family reveals the evolution and diversification of plant GPATs"

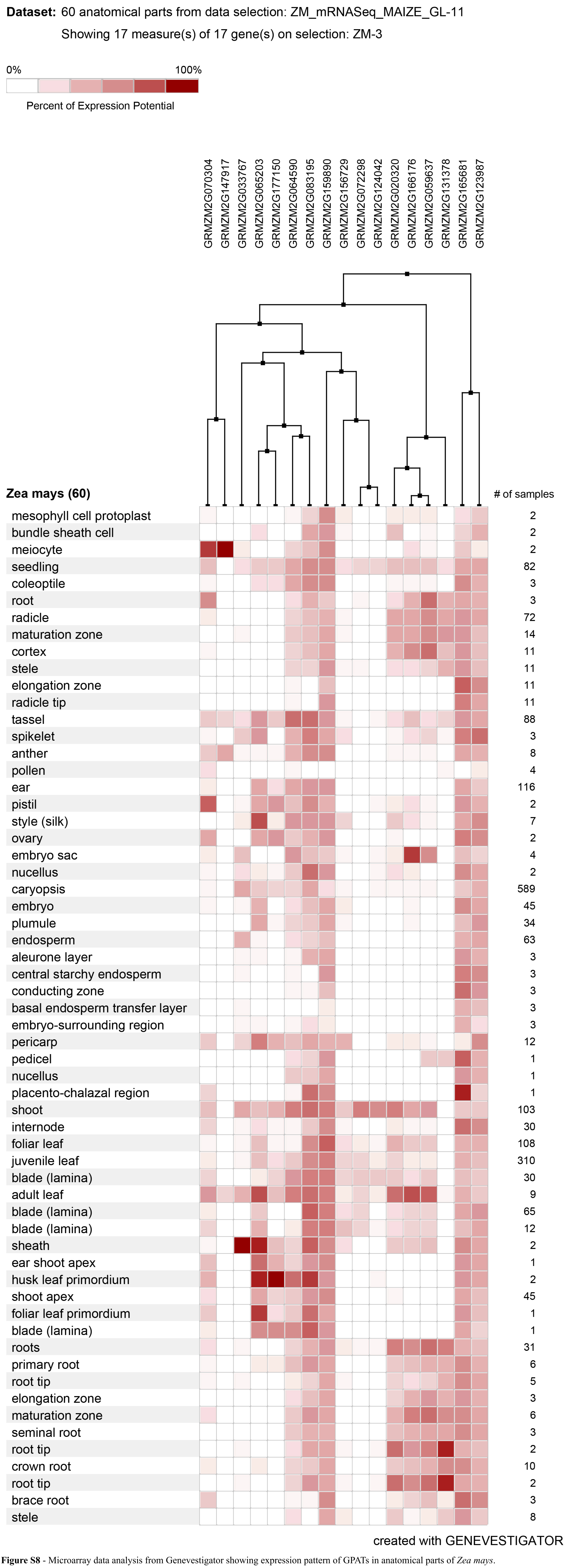


**Figure S8** - Microarray data analysis from Genevestigator showing expression pattern of GPATs in anatomical parts of *Zea mays*.
